# The vascular flora of the Comino archipelago (Maltese Islands)

**DOI:** 10.3897/phytokeys.272.184198

**Published:** 2026-04-08

**Authors:** Stephen Mifsud, Daniel Pavon, Frédéric Médail

**Affiliations:** 1 Environmental Innovation and Greening Directorate, Ministry for Gozo and Planning, St. Francis Square, Victoria, Gozo, Malta Aix Marseille Université, Avignon Université Aix-en-Provence France; 2 Institut méditerranéen de biodiversité et d’écologie (IMBE), Aix Marseille Université, Avignon Université, CNRS, IRD. Campus Aix, Technopôle de l’Environnement Arbois-Méditerranée, 13545 Aix-en-Provence, cedex 4, France Environmental Innovation and Greening Directorate, Ministry for Gozo and Planning Victoria Malta

**Keywords:** Checklist of plants, endemic plants, Mediterranean Islands, vascular flora

## Abstract

A comprehensive analysis of published records of terrestrial vascular plants reported from the Comino archipelago (Maltese Islands) since the mid-1800s and of plants recorded by us between 2008 and 2025 was combined to synthesise the first annotated and comprehensive checklist of vascular plants of Comino. A total of 328 observations were made, of which 78 are new records for Comino and two are recorded for the first time in the Maltese Islands. The flora consists of 490 vascular plant species. This study identifies and examines ecologically sensitive species on the Comino archipelago, including 58 strictly and legally protected species, 21 endemics, and several other plants that are red-listed as threatened species or are very rare in the Maltese Islands (e.g., *Euphorbia peplis*, *Hornungia procumbens*, and *Malva setigera*). Alien species are also reviewed, and 12 out of the 61 recorded species are declared invasive for Malta, but only a few are effectively invasive on Comino (e.g., *Acacia saligna*, *Ailanthus altissima*, and *Oxalis pes-caprae*). In addition, this account offers a concise yet comprehensive overview of Comino’s geology, natural habitats, climate, and anthropogenic history. More critically, it identifies the principal threats and pressures affecting the island, which have contributed, at least in part, to the disappearance of approximately 160 species previously recorded but not observed for several decades. As highlighted in previous studies, the persistent issue of over-tourism on Comino over the past 15–20 years warrants urgent attention, as the most recent reports indicate that some 10,000 visitors flock to the small beaches of the Blue Lagoon and Santa Marija Bay, which, as also demonstrated in this report, overwhelm and disturb this ecologically sensitive Natura 2000 archipelago.

*Dedicated to Salvu Vella, known affectionately as il-Venew, one of the two lifelong inhabitants of Comino. His commitment to the island’s natural environment through the careful tending of his fields, his guardianship of its landscapes, and his quiet, daily work to safeguard its biodiversity has helped preserve the character and charm of Comino for generations. This study is offered in recognition of his enduring presence, his invaluable contribution to the island’s natural heritage, and his helping hand and hospitality when we met him during our field visits*.

## Introduction

With about 11,100 islands and islets, of which about 250 are regularly inhabited by humans, the Mediterranean Basin represents one of the regions of the world with the most islands and archipelagos (Médail 2017, 2022). Most of them are small islands and islets, since only ca. 2220 islands are larger than 0.01 km^2^ (Santi et al. 2024). Small Mediterranean islands play a pivotal role in preserving coastal plant biodiversity, acting as refugia for rare, endemic, and threatened species. Their geographic isolation, combined with varied microclimates and geological substrates, has fostered the evolution of unique plant communities that are often absent from larger landmasses. These islands host a rich mosaic of coastal habitats, including dunes, cliffs, salt marshes, coastal garigue, and phrygana, that support specialised flora adapted to extreme conditions, such as drought, salinity, high temperatures, and wind exposure. Traditional land-use practices, such as low-intensity grazing and terracing, have historically maintained ecological balance, allowing native vegetation to thrive (Médail and Pasta 2024).

However, these fragile insular ecosystems face mounting pressures from tourism, invasive species, land reclamation, and climate change, which threaten the integrity of plant assemblages and the ecological services they provide (Médail 2017). Conservation of small island flora is essential not only for maintaining regional biodiversity but also for preserving unique genetic resources, for their ecological resilience in the face of environmental change, and for their role in regional biodiversity. Targeted research, habitat protection, and sustainable management strategies are urgently needed to safeguard these ecological treasures and ensure their continued contribution to the Mediterranean’s natural heritage.

These aspects are particularly significant within the Maltese archipelago, which comprises five main islands located about 90 km from the south coast of Sicily (Southern Italy) and about 290 km from Tunisia (35°55'4.7028"N, 14°24'35.7948"E): Malta (Maltese: Malta), Gozo (mt: Għawdex), Comino (mt: Kemmuna), St. Paul’s Island (mt: Selmunett), and Filfla (mt: Filfla). The first three are inhabited and are the largest in surface area; the other two are very small and uninhabited (Camilleri et al. 2012). Other smaller islets and rocks are scattered in close vicinity to the shore, the most significant being Ġebla tal-Ġeneral, Ġebla tal-Ħalfa, and Ħaġra taċ-Ċawl, all about 100 m off the coast of Gozo. Maltese ecosystems were impacted by humans early on, stretching back at least ~7500 years BCE (Marriner et al. 2012), and the two main islands (Malta and Gozo) are among the most modified by humans in the Mediterranean Basin (Camilleri et al. 2012).

The small island of Comino and its satellite islets have, fortunately, suffered far reduced destructive human impacts to this day. Thus, the conservation of Comino’s terrestrial biodiversity is a priority for the Maltese archipelago, as Comino and its islets constitute an important refuge for species and unique coastal ecosystems. The Maltese flora is a composition of Sicilian, Aegean/Eastern Mediterranean, and Tunisian/North African flora that are not found anywhere else, and much of it has been degraded on the main islands but conserved on the Comino archipelago. Despite several botanical surveys since the 19^th^ century and a preliminary checklist that was published over 100 years ago (Sommier and Caruana Gatto 1915) (see History of botanical explorations), there has not been an exhaustive collaboration to document thoroughly the floristic studies devoted to the whole Comino archipelago.

Hence, this work aims to fill a gap by providing a comprehensive checklist of the vascular flora of Comino Island and its satellite islets, which form a small archipelago including six island entities with terrestrial vascular plant species. This paper aims to provide a detailed synthesis of all floristic references available in the literature, supplemented by intensive field research (23 sessions between 2008 and 2025), in order to obtain the most accurate possible knowledge of the vascular plant richness and composition of Comino Island and its satellite island (Cominotto) and islets.

## Presentation of the Comino archipelago

### Physiography

**Figure 1.**
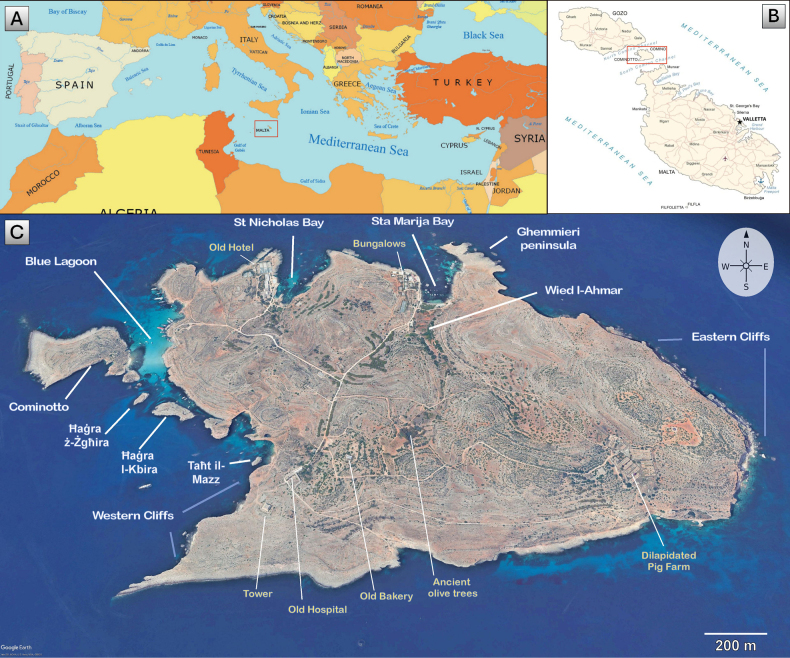
An annotated map of the Comino archipelago situated in the Maltese Islands (**A**), between Malta and Gozo islands (**B**), with the main Comino Island surrounded by the islets of Cominotto, Ħaġra ta’ bejn il-Kmiemen iż-Żgħira (Ħaġra ż-Żgħira), Ħaġra ta’ bejn il-Kmiemen il-Kbira (Ħaġra l-Kbira), Ħaġra ta’ taħt il-Mazz (Taħt il-Mazz), and other known landmarks, including the three main beaches (Blue Lagoon, Saint Nicholas Bay, and Santa Marija Bay) (**C**).

Comino itself is a small archipelago situated between Malta and Gozo (1.4 km to Gozo – 1.7 km to mainland Malta), consisting of the largest island of Comino (275 ha); a smaller island called Cominotto (mt: Kemmunett) (10.3 ha); and some islets and shallow rocks, of which the most significant are the following: Large Blue Lagoon Rock (mt: Ħaġra ta’ Bejn il-Kmiemen il-Kbira), Small Blue Lagoon Rock (mt: Ħaġra ta’ Bejn il-Kmiemen iż-Żgħira), Comino Cliff-face Rock (mt: Ħaġra ta’ Taħt il-Mazz), and Għemmieri Rocks (mt: L-iskolli tal-Għemmieri), all within 50–120 m away from the shores of Comino Island. The highest point of Comino Island is about 75 m above sea level (Boffa 1966; Pedley et al. 1978; Camilleri 2004), which is remarkably lower than that of Gozo (ca. 190 m a.s.l.) and mainland Malta (252 m a.s.l.) (Schembri 1997). Sizes and environmental characteristics of the small islands and islets of the Comino archipelago are given in Table 1 and their location in an annotated map (Fig. 1).

**Table 1.** Names, dimensions, and geological data, including an indication of vegetative cover for each island/islet of the Comino archipelago.

Names for the Comino archipelago		Distance from Comino	Peak height (asl)	Approx. surface area (m^2^)	Topo-graphical inclination	Vegetative cover
Maltese	English					
Kemmuna	Comino	n/a	75 m	2,750,000 (275 ha)	S to N	High
Kemmunett (Fig. 2A)	Cominotto	125 m	34 m	103,000 (10.3 ha)	S to N	Moderate
Ħaġra l-Kbira ta’ bejn il-Kmiemen (Fig. 2B)	Large Blue Lagoon Islet	90 m	18 m	9,220 (0.92 ha)	S to N	Moderate
Ħaġra ż-Żgħira ta’ bejn il-Kmiemen (Fig. 2C)	Small Blue Lagoon Islet	110 m	14 m	1,940 (0.19 ha)	W to E	Low
Ħaġra ta’ taħt il-Mazz (Fig. 2D)	Pigeon Rock	20 m	22 m	990	E to W	Low
Ġebla Tal-Ponta Rqiqa	Lantern Rock	30 m	12 m	140	None	Negligible
Ġebla ta’ taħt il-Batterija	Battery Rock	4 m	4 m	120	E to W	None
Skoll tal-Għemmieri	Għemmieri rocks	10–82 m	1–3 m	700	None	None

The larger islands (Comino and Cominotto) are well vegetated and include endemic species, but the minor islets are too small (typically less than 15 × 15 m) and shallow for harbouring any significant vegetation. These rocks are located along the eastern coastline of Comino and beneath the west-facing cliffs near the Wignacourt Tower (Camilleri 2004). Among the islands and islets of this archipelago, six of them harbour significant vegetation, and they are considered as “Small Mediterranean Islands” according to the definition of the PIM Initiative (https://initiative-pim.org).

The population density on Comino Island has always been very low when compared to its larger sister islands, i.e. Malta (population density ca. 2160 persons/km^2^ in 2024) and Gozo (600 persons/km^2^) (NSO 2025). Comino Island, though covering only 2.75 km^2^ and now inhabited by just two elderly individuals, experiences a substantial seasonal surge in population density. During the late spring, summer, and early autumn, the island receives an estimated 4000 to 5000 daily visitors (Fig. 6C, D), significantly altering its landscape for an extended period (Ganado 2016; Debono 2022). This influx transforms Comino into a densely populated environment, despite its minimal permanent residency (Sansone 2022).

### Geology and geomorphology

**Figure 2.**
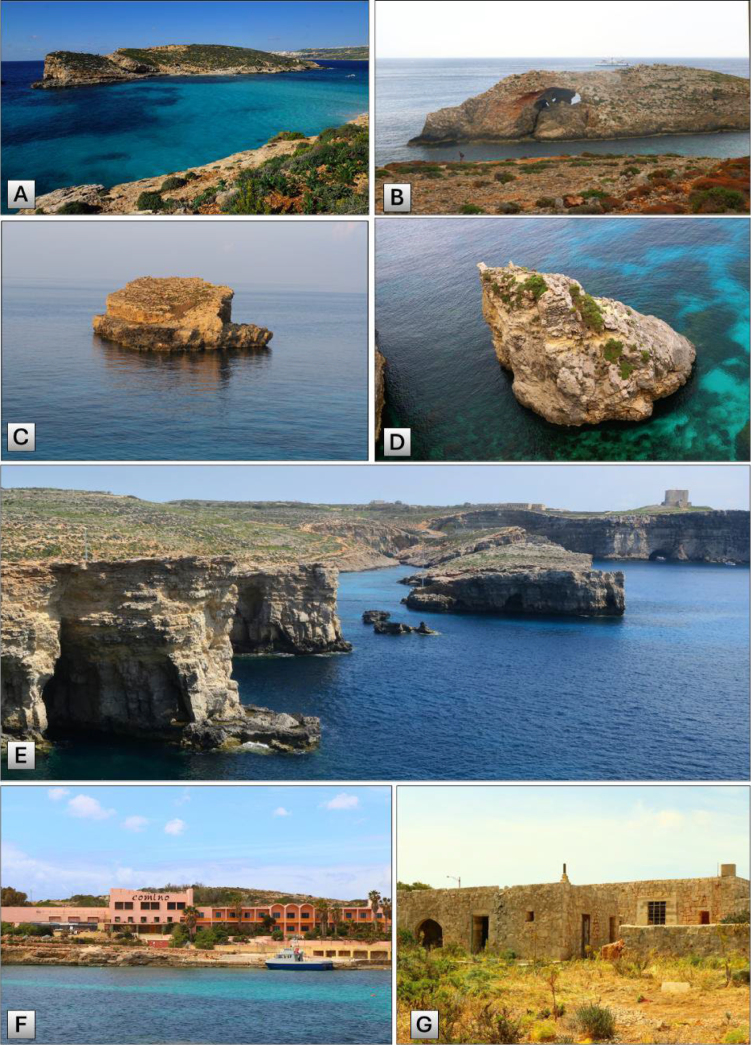
Cominotto and the three satellite islets and some iconic locations at Comino: **A**. Cominotto (104,000 m^2^); **B**. Ħaġra l-Kbira ta’ bejn il-Kmiemen (9,220 m^2^); **C**. Ħaġra ż-Żgħira ta’ bejn il-Kmiemen (1,940 m^2^); **D**. Ħaġret ta Taħt il Mazz (990 m^2^); **E**. Southern coast of Comino known as Bejn il-Kmiemen and representing a partially submerged valley; **F**. The iconic pink-coloured Comino Hotel operated between the late 1960s and summer 2019; **G**. Old bakery of Comino from the late 19^th^ C.

From the five sedimentary layers that characterise the Maltese Islands [Lower Coralline Limestone, Globigerina Limestone, Greensand, Blue Clay, and Upper Coralline Limestone listed from the lowest to the uppermost layer (Pedley et al. 1976; Schembri 1997)], Comino is predominantly composed of Upper Coralline Limestone, with traces of exposed Blue Clay present at sea level along the eastern cliffs of the island (Camilleri 2004). This is because, during seismic activities in the late Miocene epoch, approximately five to seven million years ago, most of the island’s landmass submerged, leaving only the uppermost layer of Coralline Limestone exposed (Pedley et al. 1978; Camilleri 2004; Camilleri et al. 2012). Interestingly, Comino features one of the thickest layers of Upper Coralline Limestone in the Maltese Islands (ca. 75 m deep), located in a hilly area at the eastern cliffs (Pedley et al. 1978; Camilleri 2004), and is almost entirely composed of the tal-Pitkal member of this limestone (Camilleri et al. 2012). Quaternary deposits, characterised by a thin layer of hardened rust-coloured soil or friable rock, are found in the south of the island close to the tower, such as in areas known as Bejn il-Kmiemen (Fig. 2E), Bejtet il-Fenek, Wied Skalanova, as well as in the northern part of Cominotto (Camilleri 2004).

The surface of Comino is rather flat compared to the hilly topography of Gozo and the faulting/valley-bearing terrain of mainland Malta. However, its gentle tilting towards the north and a few small, gently sloping hills to the east provide run-off water, which has gradually eroded a shallow valley with a few tributaries called Wied l-Aħmar (the red valley, for the presence of reddish Quaternary soil deposits), sometimes also referred to as Wied tal-Kola. The watercourse is short-lived and runs in a northerly direction, flushing into the sea off Santa Marija Bay. This is the largest valley system in Comino.

### Climate

The Maltese Islands are characterised by a Mediterranean climate, with prolonged, hot, and arid summers, contrasted with relatively mild and moist winters (Lanfranco 1995; Schembri 1997; Galdies 2011). Annual precipitation averages approximately 540 mm, with the majority (around 85%) occurring between October and March (Lanfranco 1995; NSO 2022; MIA 2024), thereby accentuating the pronounced seasonality of rainfall events in the region, which features distinct wet (approximately five months) and arid (approximately seven months) seasons. Monthly mean temperatures typically range from 12 °C in winter to 26 °C in summer (averaged over day and night). Extreme temperatures are uncommon, but they seldom drop below 5 °C during winter nights or exceed 37 °C in summer. Yet, high extremes of > 40 °C during short periods of heatwaves have been frequently reported over the last 30 years (Galdies 2011; Times of Malta 2025b).

The islands are further characterised by considerable wind exposure and abundant sunshine throughout the year, resulting in notably high evapotranspiration rates that may reach up to 942 mm annually. Average wind speeds range from about 6.5 knots (summer) to 10 knots (Jan–Apr) annually, with gusts reaching 45–48 knots in winter. Wind direction is predominantly (57%) from the northwest (NSO 2022). These elevated figures have significant implications for both soil moisture retention and the physiological stress experienced by native flora (Schembri 1997). The proximity of the Maltese Islands to the surrounding Mediterranean Sea not only modulates the thermal regime, reducing temperature extremes, but also elevates soil salinity, particularly in coastal and exposed areas. Consequently, vegetation communities are predominantly composed of halophytic and xerophytic species, which have evolved specific adaptations to cope with both saline substrates and persistent mechanical stress from high winds (Lanfranco 1995). These climatic parameters play a critical role in shaping the archipelago’s ecological characteristics and biodiversity, influencing the distribution and resilience of native plant and animal communities.

Comino has a lower water retention capacity compared to the larger Maltese islands due to its flat terrain, lacking hills or deep valleys. Its surface is predominantly composed of Upper Coralline Limestone with minimal clay content, and it offers scarce shade from tree canopies. The island is also more vulnerable to wind and salinity, particularly from the northeast and southwest winds, which strike it directly from the open sea. In contrast, the northwest winds are partially shielded by Gozo, while mainland Malta buffers the southeast winds. As a result, Comino is noticeably more arid and slightly warmer than Malta and Gozo. Although there is no meteorological station on Comino Island, we can estimate that it is noticeably more arid and somewhat warmer than Malta and Gozo.

### Toponomy and historical overview

Comino Island has been known by various names throughout Antiquity, reflecting its cultural and geographic significance. Around 490 BCE, navigator Scillace labelled it *Lampas* on his Mediterranean map, possibly a Phoenician name, though it may have referred to Lampedusa (Boffa 1966; Farrugia Randon and Farrugia Randon 1995). Later, it was called *Hephaestia*, a Greek term linked to volcanic origins. In the second century BCE, during the Roman period, it was known as *Chemmona*, likely derived from the Greek *kineni*, meaning “adjacent”, referencing its proximity to Malta and Gozo (Boffa 1966). By the 9^th^–10^th^ centuries CE, Arab sources referred to it as *Kemmuna*, the name still used in Maltese today; it is possibly a linguistic evolution from Greek or a reference to cumin cultivation on the island (Boffa 1966; Farrugia Randon and Farrugia Randon 1995). Yet its origin remains a subject of debate and mystery.

Despite being isolated, small, and lacking natural resources, Comino Island was inhabited for more than 4000 years, with evidence dating back to the Bronze Age (2500–800 BCE), as indicated by typical pottery found in the northern bays of Comino (Bugeja 2004). In addition, the Phoenician name the island had in the remote past and a sarcophagus found at Wied Ernu (south of Comino) indicate an established Phoenician presence on Malta, which reached Malta about 700–800 years BCE. Moreover, further evidence and artefacts found on Comino, which belong to the Romans who colonised Malta in 218 BCE and remained for some 700 years, include a skeleton buried beneath two amphora fragments, lead pipes, and pottery sherds (Bugeja 2004).

In the early Middle Ages, Comino was known for the cultivation of cumin, which was likely introduced, at least on a large scale, by the Arabs who arrived in Malta in the late 9^th^ century CE (Farrugia Randon and Farrugia Randon 1995). Some historians even suggest that the Arabs gave the island the name Comino because of its large cumin production (Boffa 1966). In later times of the Mediaeval Period until the beginning of the 17^th^ C., Comino was inhabited by pirates who used to hide in caves or secluded creeks, caverns, and inlets and organise corsair attacks on merchant ships travelling close by, often crossing between Malta and Gozo with food supplies (Boffa 1966; Farrugia Randon and Farrugia Randon 1995). One of the caves on Comino is indeed named Għar il-Pirati, meaning “Pirate’s Cave”, according to Boffa (1966). The persistent corsair activity originating from Comino, which had long resulted in the loss of ships belonging to the Knights of St. John, was effectively curtailed in 1618 with the construction of the Santa Marija Tower. Commissioned by Grandmaster Alof de Wignacourt, the tower was fortified with cannons and a garrison of soldiers, serving as a strategic deterrent against piracy in the region.

Historical accounts mention that between 1799 and the 1970s, Comino was much more populated. When Malta revolted against the French in September 1798 and was then taken under British Rule in September 1800, many French supporters were exiled from Malta and Gozo to Comino, turning the island into a concentration camp with some 150 persons kept there (Falzon 2004) and possibly marking the largest population for Comino. The tower then served as a prison until some years after World War II. The isolation of Comino made it an excellent prison, as it was very difficult for anyone to escape to the mainland at that time; there was nowhere to hide and live, and anyone who attempted an escape from the tower was shot (Farrugia Randon and Farrugia Randon 1995).

According to a few census figures recorded by the British between the late 19^th^ and early 20^th^ centuries, the population of Comino fluctuated between 30 and 70. The opening of an isolation hospital in 1912 to treat patients with cholera and plague likely contributed to the increase in Comino’s population. Then, in 1926, Comino was leased to Captain Arthur Zammit Cutajar, who made great efforts to turn the island into an agricultural business, attracting some 65 farmers to transfer to the island and earn their living (Farrugia Randon and Farrugia Randon 1995). Although not officially documented, the community of farmers, prisoners, patients, and hospital staff may have supported approximately 100 people living on Comino in the 1930s and 1940s. The number of people in Comino dwindled decade after decade from the late 1960s (approximately 60 people lived there then), when the farming business run by Zammit Cutajar was stopped. In 1960, the government leased Comino to a new company branded as the Comino Development Co. Ltd., whose target was to turn Comino into a tourist destination (Farrugia Randon and Farrugia Randon 1995), but this project was shortly abandoned. By the early 1990s, only one farming family had survived and still lived on Comino, now comprising just two elderly people (Salvu Vella, one of the residents on Comino pers. comm., May 2023).

### Human activity and pressures

Boffa (1966) reports ancient documents from 1797 that demonstrate that honey was produced on Comino and sold to the Knights stationed at the Santa Marija Tower. He also alludes to the fact that, apart from cumin, as already mentioned, cotton was also cultivated on Comino, and both yielded a lot of profit, which persisted until the early 20^th^ century. This indicated that Comino was inhabited by farmers more than 1000 years ago. Nevertheless, as stated above, the population of Comino was always small.

Due to limited interference and disturbance from human activity, Comino’s natural habitats and biodiversity remained well conserved compared to those on Gozo and the mainland of Malta. This is one of the reasons why the entire archipelago of Comino was designated as a terrestrial Natura 2000 protected site (MT0000017 – Kemmuna u l-Gżejjer ta’ Madwarha (ADI 2014a, 2014b)) within the European Habitat Directive in 1992 and is one of the largest protected areas in the Maltese Islands (Camilleri et al. 2012).

In fact, the number of main buildings on the island is currently limited to only five, namely, a coastal defence tower known as Santa Marija or Wignacourt Tower (built in 1618); an abandoned isolation hospital (1912); a hotel (Fig. 2F) and some bungalows (1964–1967), which stopped operating in 2019; an abandoned bakery of the late 19^th^ C. (Fig. 2G); and a dilapidated pig farm (1979–80). There are also minor buildings, such as a chapel, a police station, an old battery, a water pumping station, and some electricity substations. Interestingly, the island has no asphalt roads, and vehicles are focused on the Blue Lagoon area and, when it was in operation, the hotel area, hence between St. Nicholas and Santa Marija Bay. Nevertheless, the magnitude of human presence and economic activities on Comino has never compared with those of mainland Malta and Gozo until recently.

However, in the last three decades, the Maltese Islands have given top priority to mass tourism. The natural immunity that Comino has been blessed with for so long (see examples by Bonello 2024) is now in jeopardy from barely controlled tourism and new accommodation. Mass tourism gradually began to affect the island (Camilleri et al. 2012), not only during the three summer months but also in autumn and spring. For instance, the owners of the old hotel are about to demolish it and rebuild a large 6-star hotel. They also showed their intention to sell the bungalows to the public sector, which can then lead to the formation of a small hamlet near the coast of Santa Marija Bay and further urbanisation (Croce 2024). These huge and deleterious projects have sparked significant controversy and protests from environmental NGOs and the public (Croce 2023, 2024; Times of Malta 2024, 2025a).

### Natural habitats

Comino Island features a predominantly exposed, arid, rocky terrain, which gives rise to many associated habitats, namely vegetated rocky shores, rupestral communities at the cliffs, phrygana, garigue, low pre-desert scrub, and large steppic areas (once fields with shallow soil), which are sometimes manifested as mosaics and hybrid ecotones of each.

One of the most noticeable characteristics of the archipelago is the absence of clay and clayey soil, which gives Comino a distinctly different natural character compared to Malta and Gozo. Some cultivated areas farmed by the two inhabitants of Comino are still in use, as are some fields with introduced trees, which were managed by BirdLife (Camilleri et al. 2012).

A main factor leading to Comino being more arid than its sister islands is the absence of significant perched aquifers that escape over the water-impermeable Blue Clay layers to form long-lasting water springs and enduring valleys. In addition, there are barely any shaded areas from hills, deep valley banks, boulder scree, and similar features; hence, the island is prone to full exposure to the sun, strong wind, and sea spray. One must remember that the distance from the seashore to the central regions of Comino is only 700 m, so most of the island is affected by salt spray on windy days. Hence, the vegetation of Comino is predominantly xerophilic, sclerophyllous, and halophilic, which is typical of small islands in the Mediterranean region (Camilleri et al. 2012).

Wetland habitats are indeed rare on Comino and absent on Cominotto and the other islets. Wied l-Aħmar is the largest valley system on Comino, whose water supply supports slightly different habitats from the predominantly rocky surroundings. Nowadays, only a remnant semi-wetland and hygrophyte community can be observed, with surviving patches of *Arundo donax* L., *Juncus hybridus* Brot., and *Vitex agnus-castus* L.

A small marsh existed near the sandy bay of Santa Marija (Boffa 1966; Camilleri et al. 2012), with a presumed richer biodiversity, including species characteristic of wetlands and semi-wetlands. For example, according to Camilleri et al. (2012), some surviving stands of *Phragmites australis* (Cav.) Steud. growing in this marsh were transplanted to another site in Comino, known as L-Art Ħażina (an artificial pond), before the marshland was bulldozed and turned into a camping site in 1991. In addition, a small maquis, composed of olive, almond, and carob trees, is found along one side of this valley. Another small marshland was located near San Niklaw Bay, which was also completely removed to create space for a tourist recreational area adjacent to the Comino Hotel (Camilleri et al. 2012). This valley played a crucial role in providing fresh water to farming communities, where many of their fields and agricultural activities were situated (Farrugia Randon and Farrugia Randon 1995). Evidence is left by the numerous terraced fields surrounded by dry rubble walls located in the vicinity of this valley. Moreover, when the British invested in agriculture on Comino, water from this valley was harvested by building a water reservoir slightly below ground level with pumping facilities located close to the mouth of the valley, about 100 m behind the chapel (Farrugia Randon and Farrugia Randon 1995). Some wells and two boreholes are also present near the valley (Camilleri et al. 2012), and Farrugia Randon and Farrugia Randon (1995) mention a total of nine boreholes on the island.

**Figure 3.**
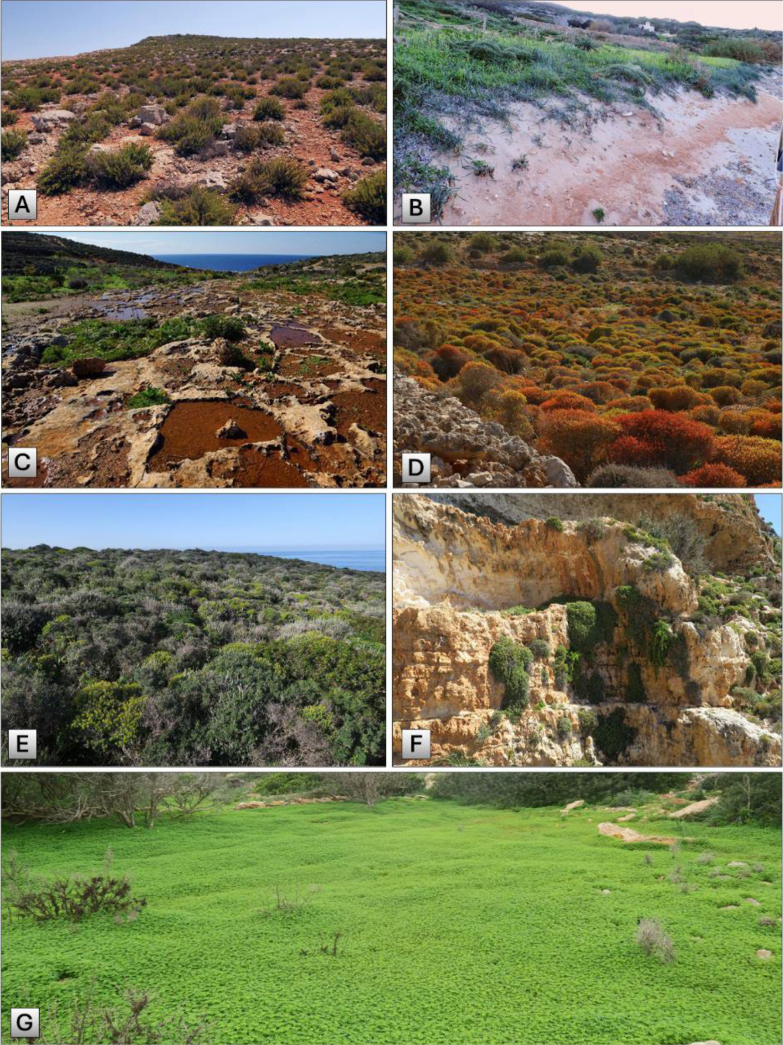
Principal natural habitats from the Comino archipelago: **A**. Vegetated sea cliffs of the Mediterranean coasts; **B**. Embryonic shifting dunes; **C**. Mediterranean temporary ponds; **D**. Thermo-Mediterranean and pre-desert scrub; **E**. West Mediterranean clifftop phrygana; **F**. Calcareous rocky slopes with chasmophytic vegetation; **G**. Steppic areas dominated by ruderals or alien species such as *Oxalis pes-caprae*.

A notable habitat on Comino is the small sand dune in Santa Marija Bay, which spans approximately 120 m in width. The importance of Malta’s natural sand dunes is reflected in the fact that they are all protected as Natura 2000 sites. A major part of this bay is now overrun by trees of *Tamarix africana* Poir., which grow peculiarly into the mobile and embryonic parts of the sand dune (Camilleri et al. 2012). Some photos of Santa Marija Bay from 1983 showed that these trees reached the shore, and this population existed at the beginning of the 1960s (Carmelina Borg Cuschieri pers. comm., July 2024). However, a photo from the 1930s shows instead a population of *Vitex agnus-castus*, suggesting that the *Tamarix* population may not be autochthonous to the Santa Marija Bay shore. The remaining unwooded part of the sand dune – a stretch of ca. 40 × 10 m – is exposed vegetated sand, which once had a rich diversity of psammophilous species (known thanks to historic records) until aggressive touristic activities ruined this small dune persistently during the last 15–20 years, and currently it is heavily degraded by mass tourism (Fig. 6B, C, D). Its degradation is manifested by a low diversity of sand-dune species and the increasing presence of non-psammophytes, such as *Pistacia lentiscus* L., *Cynara cardunculus* L., *Oxalis pes-caprae* L., and *Dittrichia viscosa* (L.) Greuter subsp. viscosa. Comino also comprises a few small temporary ponds, notably on rock slabs, which are particularly flooded after heavy precipitation, typically at the end of October (F. Médail pers. obs. Nov 2018) (Fig. 3C).

The habitat types found on Comino can be categorised as follows:

1. 1240 - Vegetated sea cliffs of the Mediterranean coasts with endemic *Limonium* spp. (Fig. 3A).
2. 1420 - Mediterranean and thermo-Atlantic halophilous scrubs (*Sarcocornietea fruticosi*).
3. 2210 - Embryonic shifting dunes (Fig. 3B).
4. 3140 - Hard oligo-mesotrophic waters with benthic vegetation of *Chara* spp..
5. 3170 - Mediterranean temporary ponds (Fig. 3C).
6. 5330 - Thermo-Mediterranean and pre-desert scrub (Fig. 3D).
7. 5410 - West Mediterranean clifftop phrygana (Fig. 3E).
8. 6220 - Pseudo-steppe with grasses and annuals of the *Thero*-*Brachypodietea*.
9. 8210 - Calcareous rocky slopes with chasmophytic vegetation (Fig. 3F).
10. 92D0 - Southern riparian galleries and thickets (*Nerio*-*Tamaricetea* and *Securinegion tinctoriae*).

Upon visiting Comino, one gains the impression that its vegetation differs from that of Malta and Gozo; indeed, as demonstrated below, Comino features a distinct palette of species and vegetation types. The relatively reduced disturbance from anthropogenic activities and from alien species is striking on Comino. For example, aside from some relict fields and old farms, there is a very minimal presence of the top invasive species, *Oxalis pes-caprae*, which is omnipresent in both Malta and Gozo. Additionally, there are a few species that are infrequent or even rare in Malta and Gozo but are very common on Comino, which will be discussed further below.

These factors, together with the lack of trees; aridity; shallow soil lacking clay and hence poor water retention; and a predominant topography of Upper Coralline Limestone, contribute to the unique landscape of Comino compared to Malta and Gozo.

### History of botanical explorations

Botanical studies in the Comino archipelago are limited and were primarily conducted by pioneering botanists over 150 years ago. The earliest records from Comino and Cominotto are available from the British visitor John Firminger Duthie, who visited Comino once in 1874. He enumerated all his records from Comino and Cominotto in a subsequent checklist, gradually published in three works (Duthie 1874, 1875a, 1875b). Duthie (op. cit.) recorded 76 species for the archipelago – 68 from Comino and 23 from Cominotto.

The Flora of Sommier and Caruana Gatto (1915) provides very valuable and comprehensive records for Comino and Cominotto by combining their records with previously published records or collections from this archipelago. This work includes three sections: (i) the main flora section, Avvertenze alla Flora Melitensis Nova *–*Dicotyledoneae and Monocotyledoneae (pp. 65–331); (ii) a summarised checklist named Prospetto delle Piante Vascolari Maltesi…. (pp. 437–477); and (iii) a checklist of plants they observed during a visit to Cominotto: “Elenco delle piante osservate nell’ isolotto di Cominotto” (pp. 478–479). In the *Prospetto*, the authors declared by footnote (n°2) that at Comino, various plants have not been indicated in the previous section (Avvertenze alla Flora) because they have seen them at various herbaria or private collections after sending the flora to print. It is hence assumed that they have prepared this *prospetto* later and included other records they studied *ex situ* after their visit. Sommier and Caruana Gatto provided 273 records from Comino and 71 from Cominotto. Five records were reported only from Cominotto, bringing the total to 278 species in the archipelago, representing 200 additions over Duthie’s work (1874, 1875a). Of these, 40 species have been retrieved from exsiccata and voucher specimens collected from Comino by other visitors/explorers, which were verified and reported by Sommier and Caruana Gatto (1915).

Twelve years later, in his descriptive flora of the Maltese Islands, Borg (1927) included many records from Comino, but there is no indication of whether they refer to the author’s personal observations or to citations from previous works. Nevertheless, the total number of records from the Comino archipelago increased to 319 species. Borg (1927) provided a significant number of 41 new records for the archipelago, including some agricultural trees and crops, but his nomenclature of taxa was not accurate, as Sommier and Caruana Gatto (1915) had noted.

The flora of Borg (1927) mentions 316 species from Comino and 59 from Cominotto (three of which are only from Cominotto), but it seems that he did not rigorously make a distinction between the two islands in his records; that is, when reporting a species from Comino, he may have also seen it in Cominotto without specifying that he saw it there as well. Examples corroborating this inaccuracy include *Lysimachia arvensis* (L.) U.Manns & Anderb. (= *Anagallis arvensis* L.), *Arisarum vulgare* O.Targ.Tozz., and *Centaurium pulchellum* (Sw.) Hayek ex Hand.-Mazz., Stadlm., Janch. & Faltis, which are all frequently found in Cominotto and already reported from there by previous authors. Moreover, he reported 12 fewer records from Cominotto than the preceding flora by Sommier and Caruana Gatto (1915).

Over the following five decades, there was no significant systematic floristic work until Haslam et al. (1977) published an updated flora of the Maltese Islands, including locations for most species. Haslam et al. (1977) cited most records previously cited from Comino by other authors but added 23 new records, totalling 320 species from the archipelago: 318 from Comino and 56 from Cominotto. However, a limited distinction is made between previously reported records and their own and new observations. Moreover, the peculiar numbers of records they provide (at least for mainland Comino) pose questions and doubt on the accuracy and comprehensiveness of their work because the total number of records over Borg (1927) is only one more species, indicating that they omitted some previously reported records such as *Phragmites australis*, *Tamarix africana*, and *Vitex agnus-castus*. Moreover, the 56 records attributed to Cominotto are slightly fewer than those reported by Borg (1927).

After these major works, there have been sporadic additions by a few authors published after the year 2000. From their work on Maltese orchids, Bartolo et al. (2001) mentioned some new records from Comino and Cominotto. In a scholarly article on the plants of Comino, Lanfranco (2004) focused only on the rare or interesting plants found on this island. He mentions 35 species, four of which are new: *Asphodelus aestivus* Brot. (= *A. ramosus* L.), *Linum bienne* Mill., Polycarpon tetraphyllum subsp. diphyllum (Cav.) O.Bolòs & Font Quer., and *Scilla sicula* Tineo ex Guss. Tabone (2008) adds *Orobanche cernua* Vell., new for Comino. Sciberras and Sciberras (2010) published checklists of species from the island of Cominotto and the smaller islets, but their records seem to be based only on spring flora and are rather incomplete. They reported 43 species from Cominotto, 14 from Taħt il-Mazz, 12 from Ħaġra ta’ Bejn il-Kmiemen il-Kbira, and nine from Ħaġra ta’ Bejn il-Kmiemen iż-Żgħira. Their new records comprise *Salsola melitensis* Botsch. [= *Darniella melitensis* (Botsch.) Brullo] from all islets; *Limonium melitense* Brullo from three small islets; *Pistacia lentiscus* from two islets; Matthiola incana subsp. melitensis Brullo, Lanfr., Pavone & Ronsisv. from Taħt il-Mazz islet; *Malva arborea* (L.) Webb & Berthel. from Ħaġra ta’ Bejn il-Kmiemen iż-Żgħira; and *Cynomorium coccineum* L. from Ħaġra ta’ bejn il-Kmiemen il-Kbira.

## Materials and methods

The approach taken in this study was to synthesise published records of vascular plants from Comino and its major islets and then combine and compare them with field surveys.

The study commenced with two pilot surveys: one was conducted on 14–15 May 2008 by two of us [DP and SM] and another on 17 November 2018 by [FM]. These preliminaries were instrumental in better understanding the natural habitats on Comino, the vegetative assemblages present, the current common species, and, finally, the current threats the island faces. When compared with published records, these preliminary studies revealed several discrepancies in plant composition and, in some cases, in species taxonomy, highlighting the need for an updated and comprehensive checklist of plants currently occurring on Comino. Before the onset of this study, official checklists of the vascular plants occurring in Comino and Cominotto were published over a hundred years ago by Duthie (1875b) and Sommier and Caruana Gatto (1915). Moreover, several taxa cited in historical records were found to be misapplied or misidentified, and, consequently, this study also undertook a taxonomic reassessment to correct these records, as further detailed in the Results, Discussion, and Suppl. material 1. Finally, there was a significant insufficiency of data on the autumn and winter flora because most previous surveys were conducted in spring. Surveying across all seasons of the year was thus another important component of this work, particularly on the sister islets of Comino, which had been visited only in spring.

### Published records

Published scientific work or reliable floristic accounts that were consulted to create this checklist of plants recorded from Comino prior to our surveys commencing in February 2019 consisted of: Duthie (1874, 1875a, 1875b); Sommier and Caruana Gatto (1915); Borg (1922, 1927); Haslam et al. (1977); Lanfranco (2004); Tabone (2007, 2008); Sciberras and Sciberras (2009, 2010); Casha (2017); and Mifsud (2018). We are aware of the Local Management Plans and Natura 2000 Standard Data Forms (ADI 2014a, 2014b), but these rely heavily on the published data mentioned above.

General-audience floristic works were also consulted, including Lanfranco (1969, 2003); Weber and Kendzior (2006); Lanfranco and Bonett (2018); and Casha (2017). Additional records on Comino’s ecology from sources such as Farrugia Randon and Farrugia Randon (1995) and Camilleri et al. (2012) have also been considered. However, not all relevant publications could be included, as some are not readily available to the public (such as graduate theses), while others lack scientific credibility, including those found in magazines, newspapers, social media, or non-peer-reviewed journals. On the other hand, a few personal communications from reliable authorities have been considered and indicated in this work accordingly.

The taxonomic treatment (accepted taxa) for most species follows the systematic classifications provided by World Flora Online (https://www.worldfloraonline.org/). However, when our expertise or research indicates a more appropriate taxonomy, or in cases involving recently revised or contentious taxa, we may cite the traditional taxon commonly used in Maltese literature. Nevertheless, obsolete or synonymous taxa cited by the pioneers in their historical literature (pre-1927) are also provided to provide more comprehensive coverage of what was reported in the Maltese Islands, with reference to Comino.

### Personal communications and unpublished records

This report considered personal communications, such as emails, social media interactions, and private discussions; however, no formal or specific interviews were conducted. Typically, photographic evidence was requested for verification, except when the individual involved was a qualified or experienced botanist (e.g. Edwin Lanfranco). Notably, some valuable records emerged from these personal communications and related articles. Additionally, unpublished records collected by us between 2005 and 2018, substantiated with photographs, are included herein. Records or communications originating from social media have been excluded due to the significant challenges and time required to authenticate such data at a research standard. Records reported on the online digital database iNaturalist (www.inaturalist.org) from Comino were checked on 22 August 2025. Any records that were new, rare, or not seen for a long time were included in the report.

### Site visits and surveys on Comino

A total of 21 site visits were conducted between 2019 and 2025 by one of us (SM) on the archipelago of Comino, and two other surveys were conducted on Comino Island by some of us (DP and SM in 2008, FM in 2018). More than one survey was conducted during some site visits; for example, on 15 April 2021, a survey on Ħaġra taħt il-Mazz, Ħaġra ż-Żgħira, and Cominotto was carried out. The dates of the 21 site visits and the corresponding islets surveyed are given in Table 2. The smaller rocks, which, according to Sciberras and Sciberras (2010), as well as direct observation from the mainland, exhibited no or negligible vegetation (refer to Table 1), were not visited. In addition, these rocks are difficult to access.

**Table 2.** Dates of the 21 botanical site visits conducted on the Comino archipelago between 2019 and 2025, including the specific islands where research occurred.

No.	Date	Island	No.	Date	Island
1	3/Feb/2019	Comino	13	25/Apr/2021	Comino
2	30–31/Mar/2019	Comino	14	04/May/2021	Ħaġra l-Kbira
3	4–7/May/2019	Comino	15	21/Nov/2021	Comino
4	31/Aug/2019	Comino	16	15/Mar/2022	Comino
5	19/Oct/2019	Comino	17	19/Apr/2022	Comino
6 a	04/Jun/2020	Ħaġra ta’ taħt il-Mazz	18	27/May/2022	Comino
b	04/Jun/2020	Cominotto	19	01/May/2023	Comino
7	11/Oct/2020	Cominotto	20a	26/Nov/2024	Ħaġra l-Kbira
8	01/Nov/2020	Comino	b	26/Nov/2024	Ħaġra ż-Żgħira
9	20/Mar/2021	Comino	c	26/Nov/2024	Cominotto
10	26/Mar/2021	Cominotto	21	04/Apr/2025	Cominotto
11	11/Apr/2021	Comino			
12a	15/Apr/2021	Ħaġra taħt il-Mazz			
b	15/Apr/2021	Ħaġra ż-Żgħira			
c	15/Apr/2021	Cominotto			

Thus, the scope of the study includes five islands or islets: Comino, Cominotto, Ħaġra l-Kbira ta bejn il-Kmiemen (here referred to as Ħaġra l-Kbira), Ħaġra ż-Żgħira ta bejn il-Kmiemen (referred to as Ħaġra ż-Żgħira), and Ħaġra ta’ taħt il-Mazz. These visits encompassed surveys conducted during the autumn and spring seasons, thereby ensuring comprehensive observation. Only the islet of Ħaġra ta’ taħt il-Mazz could not be visited in autumn due to unsafe and challenging access by boat.

During the first site visit, all species encountered were recorded, and a provisional working checklist was drafted with the records from the preliminary studies (see above). To facilitate on-site reporting, during successive visits, only records not encountered in previous visits were noted. These were progressively added to and combined with the checklist at every site visit. In our opinion, a comprehensive and complete checklist was assembled after 21 site visits encompassing 26 surveys, since during some visits more than one survey in different islets or islands was conducted (e.g. three surveys at the site visits of 15 April 2021 and 26 November 2024).

There have been some instances where introductions did not survive for one to a few years. For example, following attempts to restore the marshland at the back of Santa Marija Bay, some wetland and hygrophyte plants introduced in 2021–2022 died soon after. Similarly, several trees planted by Ambjent Malta since 2018–2019 (ERA 2019), which are still irrigated by a network of irrigation pipes, have died, despite several trees, such as *Vitex agnus-castus*, *Quercus ilex* L., and *Ceratonia siliqua* L., still being alive. This is due to the obvious incompatibility of the rocky, arid habitat of Comino with these trees. These species are not included as part of Comino’s flora. Hence, in this study, plants introduced less than five years ago are not considered fully naturalised or mature and are therefore not included unless, in this short period, they have truly established themselves into a reproductive population. This period is the same length as our survey duration; in other words, plants that were observed to be cultivated or irrigated during our study are excluded. These can become part of Comino’s flora when they establish themselves without intervention from humans.

### Elaboration of the final checklist

All valid records from published sources were combined with new records from our visits to synthesise a final, updated checklist for the Comino archipelago. The results are presented in various tables, including an inventory of plants for each of the major islets of Comino. The classification is primarily based on World Flora Online (WFO 2026) and Plants of the World Online (POWO 2026), with a few exceptions for which, in our experience, a different taxon should be used. Other work which was consulted with regard to the taxonomy of debatable groups and nomenclature includes Brullo and Pavone (1985); Iamonico and Alessandrini (2008); Iamonico and Del Guacchio (2017); Del Guacchio et al. (2018); Koutroumpa et al. (2018); Iamonico (2019); and Iamonico et al. (2017, 2022, 2024). The synonymous (or erroneous) taxa, as originally cited in past records, are also presented in our work. Most of the material observed on Comino was thoroughly analysed and photographed to ensure a valid taxonomic approach, including corrections or novelties. Some historical misidentifications that cannot now be verified were unavoidable in this synthesis and, giving the benefit of the doubt, are retained on the checklist. Likewise, older records not confirmed during our surveys were kept in the dataset rather than excluded on the assumption of extinction. Nevertheless, interpreting such taxa as extinct may be reasonable when a species documented more than a century ago has not been observed since. A pertinent example is *Valantia hispida* L., last reported from Comino by Duthie (1875b) and long considered absent, yet ultimately rediscovered on Gozo (Mifsud 2009).

The recorded and valid taxa, excluding those identified as erroneous, taxonomically incorrect, synonyms, or unreliable records (see Discussion), together with new records from the surveys in this study, have been combined to create the first comprehensive checklist of vascular plants occurring in the Comino archipelago (Table 5). It lists the species and their author citation, family, status in the Maltese Islands, and Raunkiaer’s life-form category (Raunkiaer 1934): Therophytes (Th), Chamaephytes (Ch), Hemicryptophytes (H), Phanerophytes (Ph and nPh), Geophytes (G), and Hydrophytes (Hy). Species are organised and sorted by their family name. The table also remarks which species are New Records and which were previously recorded but were not observed during these surveys, referred to as Lost Records. Additionally, Table 5 specifies the islands or islets on which each species was found during the surveys. Endemic species refer to those occurring only on the Maltese Islands, whereas subendemic refers to those found in southern Sicily and the pelagic islands of Lampedusa, Linosa, and Lampione.

**Table 3.** Checklist of published species from the archipelago of Comino between 1874 and 2018 by various authors [DUT: Duthie (1874, 1875a^2^); SCG: Sommier and Caruana Gatto (1915) – those they observed from herbarium material collected by others from Comino are denoted by SCG^2^; BRG^1^: Borg (1922); BRG: (1927); HAS: Haslam et al. (1977); BAR: Bartolo et al. (2001); LNF: Lanfranco (2004); S&S: Sciberras and Sciberras (2009, 2010); CAM: Camilleri et al. (2012); CSH: Casha (2017) and others (not abbreviated). Most species are given to the currently accepted taxon sensu World Flora Online (WFO 2026) and Plants of the World Online (POWO 2026).

No.	Species	Family	First citation
1	*Acacia saligna* (Labill.) H.L.Wendl.	Fabaceae	CAM
2	*Adiantum capillus-veneris* L.	Pteridaceae	SCG
3	*Adonis microcarpa* DC.	Ranunculaceae	SCG
4	*Aegilops ovata* L.	Poaceae	SCG
5	*Aetheorhiza bulbosa* (L.) Cass.	Asteraceae	DUT
6	Agave americana subsp. americana L.	Asparagaceae	HAS
7	*Ailanthus altissima* (Mill.) Swingle	Simaroubaceae	SCG
8	*Ajuga iva* (L.) Schreb.	Lamiaceae	SCG
9	*Allium cepa* L.	Amaryllidaceae	BRG
10	*Allium lojaconoi* Brullo, Lanfr. & Pavone	Amaryllidaceae	SCG
11	*Allium polyanthum* Schult. & Schult.f.	Amaryllidaceae	SCG
12	*Allium roseum* Krock.	Amaryllidaceae	BRG
13	*Allium sativum* L.	Amaryllidaceae	BRG
14	*Allium subvillosum* Salzm. ex Schult. & Schult.f.	Amaryllidaceae	BRG
15	*Allium trifoliatum* Cirillo	Amaryllidaceae	SCG
16	*Aloe vera* (L.) Burm.f.	Asphodelaceae	SCG
17	*Ambrosia maritima* L.	Asteraceae	HAS
18	*Ammi majus* L.	Apiaceae	SCG ^2^
19	*Ammoides pusilla* (Brot.) Breistr.	Apiaceae	SCG ^2^
20	Anacamptis coriophora subsp. fragrans (Pollini) R.M.Bateman, Pridgeon & M.W.Chase	Orchidaceae	DUT
21	Anacamptis pyramidalis subsp. pyramidalis (L.) Rich.	Orchidaceae	SCG
22	Anacamptis pyramidalis subsp. urvilleana (Sommier and Caruana) Landwehr	Orchidaceae	Stevens (2000)
23	*Anacamptis collina* (Banks & Sol. ex Russell) R.M.Bateman, Pridgeon & M.W.Chase	Orchidaceae	Mallia and Schembri (1991)
24	*Anchusa italica* Retz.	Boraginaceae	SCG ^2^
25	*Anemone coronaria* L.	Ranunculaceae	SCG
26	*Anthemis arvensis* L.	Asteraceae	BRG
27	*Anthemis tomentosa* Gouan ex Boiss.	Asteraceae	HAS
28	*Anthemis urvilleana* Sommier and Caruana	Asteraceae	SCG
29	*Anthoxanthum gracile* Bivon.	Poaceae	BRG
30	Anthyllis hermanniae subsp. melitensis Brullo & Giusso	Fabaceae	DUT
31	Anthyllis vulneraria subsp. maura (Beck) Maire	Fabaceae	SCG
32	*Antirrhinum siculum* Mill.	Plantaginaceae	BRG
33	*Antirrhinum tortuosum* Bosc ex Lam.	Plantaginaceae	BRG
34	*Arisarum vulgare* O.Targ.Tozz.	Araceae	SCG
35	*Arthrocnemum macrostachyum* (Moric.) K.Koch	Amaranthaceae	SCG
36	*Arum italicum* Mill.	Araceae	SCG
37	*Arundo donax* L.	Poaceae	SCG
38	*Asparagus aphyllus* L.	Asparagaceae	DUT
39	*Asperula aristata* L.f.	Rubiaceae	SCG ^2^
40	*Asphodelus ramosus* L.	Asphodelaceae	SCG
41	*Asteriscus aquaticus* (L.) Less.	Asteraceae	SCG
42	*Astragalus boeticus* L.	Fabaceae	DUT ^2^
43	*Astragalus hamosus* L.	Fabaceae	DUT
44	*Astragalus sesameus* L.	Fabaceae	DUT
45	*Atractylis gummifera* L.	Asteraceae	SCG
46	*Avena barbata* Pott ex Link	Poaceae	SCG
47	*Avena sterilis* L.	Poaceae	BRG
48	*Bellardia trixago* (L.) All.	Orobanchaceae	DUT ^2^
49	*Bellis annua* L.	Asteraceae	SCG
50	*Bellis sylvestris* Cirillo	Asteraceae	SCG ^2^
51	Beta vulgaris subsp. maritima (L.) Thell.	Amaranthaceae	SCG
52	*Biscutella didyma* L.	Brassicaceae	BRG
53	*Biscutella lyrata* L.	Brassicaceae	HAS
54	*Bituminaria bituminosa* (L.) C.H.Stirt.	Fabaceae	SCG
55	*Blackstonia perfoliata* (L.) Huds.	Clusiaceae	DUT
56	*Borago officinalis* L.	Boraginaceae	SCG
57	*Brachypodium distachyon* (L.) P.Beauv.	Poaceae	SCG
58	*Brachypodium pinnatum* (L.) P.Beauv.	Poaceae	BRG
59	*Brachypodium retusum* (Pers.) P.Beauv.	Poaceae	DUT
60	*Brassica oleracea* s.l. L.	Brassicaceae	HAS
61	*Brassica rupestris* s.l. Raf.	Brassicaceae	HAS
62	*Briza maxima* L.	Poaceae	SCG
63	*Bromus diandrus* Roth.	Poaceae	HAS
64	*Bromus fasciculatus* C.Presl	Poaceae	SCG
65	*Bromus hordeaceus* s.l. L.	Poaceae	SCG
66	*Bromus madritensis* L.	Poaceae	SCG
67	*Bromus rigidus* Roth	Poaceae	BRG
68	*Bromus tectorum* L.	Poaceae	HAS
69	*Bupleurum lancifolium* Hornem.	Apiaceae	SCG
70	*Cakile maritima* Scop.	Brassicaceae	SCG
71	*Calendula arvensis* L.	Asteraceae	BRG
72	*Calendula suffruticosa* Vahl.	Asteraceae	HAS
73	*Campanula erinus* L.	Campanulaceae	SCG
74	*Capparis orientalis* Duhamel	Capparidaceae	SCG
75	*Capsella bursa-pastoris* Medik.	Brassicaceae	SCG ^2^
76	*Carduus marmoratus* (Boiss. & Heldr.) P.H.Davis	Asteraceae	SCG
77	*Carduus pycnocephalus* L.	Asteraceae	SCG
78	*Carex divisa* Huds.	Cyperaceae	DUT
79	*Carlina involucrata* Poir.	Asteraceae	SCG
80	*Carpobrotus acinaciformis* (L.) L.Bolus	Aizoaceae	CAM
81	*Carthamus tinctorius* L.	Asteraceae	BRG
82	*Catapodium hemipoa* (Delile ex Spreng.) Laínz	Poaceae	HAS
83	*Catapodium marinum* (L.) C.E.Hubb.	Poaceae	SCG
84	*Catapodium rigidum* (L.) C.E.Hubb.	Poaceae	DUT
85	*Catapodium zwierleinii* (Lojac.) Brullo	Poaceae	HAS
86	*Centaurea melitensis* L.	Asteraceae	SCG
87	*Centaurea nicaeensis* All.	Asteraceae	BRG
88	*Centaurium erythraea* Rafn	Gentianaceae	SCG
89	*Centaurium pulchellum* (Sw.) Hayek ex Hand.-Mazz., Stadlm., Janch. & Faltis	Gentianaceae	DUT
90	*Cerastium glomeratum* Thuill.	Caryophyllaceae	SCG ^2^
91	*Ceratonia siliqua* L.	Fabaceae	SCG
92	*Chenopodium murale* L.	Amaranthaceae	SCG
93	*Chiliadenus bocconei* Brullo	Asteraceae	BRG
94	*Chrozophora tinctoria* (L.) A.Juss.	Euphorbiaceae	BRG
95	*Cichorium spinosum* L.	Asteraceae	SCG
96	*Clinopodium nepeta* (L.) Kuntze	Lamiaceae	SCG ^2^
97	*Colchicum cupanii* s.l. (L.) DC.	Colchicaceae	SCG
98	*Convolvulus althaeoides* L.	Convolvulaceae	DUT
99	*Convolvulus arvensis* L.	Convolvulaceae	SCG
100	*Convolvulus elegantissimus* Mill.	Convolvulaceae	BRG
101	*Convolvulus lineatus* L.	Convolvulaceae	SCG
102	*Convolvulus oleifolius* Desr.	Convolvulaceae	DUT
103	*Convolvulus pentapetaloides* L.	Convolvulaceae	SCG
104	*Coronilla scorpioides* (L.) Koch	Fabaceae	DUT
105	*Coix lacryma-jobi* L.	Poaceae	BRG
106	*Crassula vaillantii* (Willd.) Schoenl.	Crassulaceae	BRG
107	*Crithmum maritimum* L.	Apiaceae	SCG ^2^
108	*Crucianella maritima* L.	Rubiaceae	SCG
109	*Cuminum cyminum* L.	Apiaceae	BRG
110	*Cuscuta epithymum* L.	Convolvulaceae	SCG
111	*Cynara cardunculus* L.	Asteraceae	SCG
112	*Cynodon dactylon* (L.) Pers.	Poaceae	BRG
113	*Cynoglossum creticum* Mill.	Boraginaceae	BRG
114	*Cynomorium coccineum* L.	Cynomoriaceae	S&S
115	Dactylis glomerata subsp. hispanica (Roth) Nyman	Poaceae	BRG
116	*Daucus carota* s.str. L.	Apiaceae	S&S
117	*Daucus gingidium* L. ex DC.	Apiaceae	SCG
118	*Daucus lopadusanus* Tineo	Apiaceae	SCG
119	*Daucus rupestris* Guss.	Apiaceae	DUT
120	*Desmazeria pignatti* Brullo & Pavone	Poaceae	DUT
121	*Diplotaxis erucoides* (L.) DC.	Brassicaceae	SCG ^2^
122	*Diplotaxis viminea* DC.	Brassicaceae	SCG ^2^
123	*Dittrichia graveolens* (L.) Greuter	Asteraceae	SCG
124	*Dittrichia viscosa* (L.) Greuter	Asteraceae	SCG
125	*Draba verna* L.	Brassicaceae	DUT ^2^
126	*Drimia pancration* (Steinh.) J.C.Manning & Goldblatt	Asparagaceae	DUT
127	*Ecballium elaterium* (L.) A.Rich.	Cucurbitaceae	BRG
128	*Echium arenarium* Guss.	Boraginaceae	SCG
129	*Echium parviflorum* Moench	Boraginaceae	SCG
130	*Emex spinosa* (L.) Campd.	Polygonaceae	SCG
131	*Erigeron bonariensis* L.	Asteraceae	DUT
132	*Erodium cicutarium* (L.) L’Hér.	Geraniaceae	SCG
133	*Erodium malacoides* (L.) L’Hér.	Geraniaceae	DUT
134	*Erodium moschatum* (Burm.f.) L’Hér.	Geraniaceae	SCG
135	*Eruca vesicaria* (L.) Cav.	Apiaceae	BRG
136	*Ervilia sativa* Link	Brassicaceae	BRG
137	*Eryngium maritimum* L.	Asteraceae	SCG
138	*Eucalyptus gomphocephala* A.Cunn. ex DC.	Myrtaceae	CAM
139	*Euphorbia dendroides* L.	Euphorbiaceae	SCG
140	*Euphorbia exigua* L.	Euphorbiaceae	DUT
141	*Euphorbia melitensis* Parl.	Euphorbiaceae	DUT
142	*Euphorbia paralias* L.	Euphorbiaceae	SCG ^2^
143	*Euphorbia peplis* L.	Euphorbiaceae	BRG
144	*Euphorbia peplus* L.	Euphorbiaceae	DUT
145	*Euphorbia pinea* L.	Euphorbiaceae	DUT
146	*Euphorbia terracina* L.	Euphorbiaceae	SCG ^2^
147	*Filago pygmaea* Cav.	Asteraceae	DUT
148	*Fedia graciliflora* Fisch. & C.A.Mey.	Caprifoliaceae	SCG
149	*Ferula melitensis* Brullo, C.Brullo, Cambria, Giusso, Salmeri & Bacch.	Apiaceae	SCG
150	*Ficus carica* L.	Moraceae	SCG ^2^
151	*Filago pyramidata* s.l. C.A.May	Asteraceae	SCG
152	*Foeniculum vulgare* Mill.	Apiaceae	SCG
153	*Frankenia hirsuta* L.	Apiaceae	DUT
154	*Frankenia pulverulenta* L.	Apiaceae	DUT
155	*Fumana thymifolia* Spach	Cistaceae	BRG
156	*Fumaria agraria* Lag.	Fabaceae	SCG
157	*Fumaria flabellata* Gasp.	Papaveraceae	BRG
158	*Fumaria officinalis* L.	Papaveraceae	SCG
159	*Galactites tomentosus* Moench	Asteraceae	SCG
160	*Galium aparine* L.	Rubiaceae	BRG
161	*Galium murale* M.Bieb.	Rubiaceae	SCG
162	*Galium verrucosum* Huds.	Rubiaceae	SCG ^2^
163	*Gastridium ventricosum* (Gouan) Schinz & Thell.	Poaceae	SCG
164	*Geranium molle* L.	Geraniaceae	SCG
165	*Geranium robertianum* L.	Geraniaceae	BRG
166	*Geranium rotundifolium* L.	Geraniaceae	BRG
167	*Gladiolus italicus* Mill.	Iridaceae	SCG
168	*Glaucium flavum* Crantz	Papaveraceae	SCG
169	*Glebionis coronarium* (L.) Cass. ex Spach	Asteraceae	SCG
170	*Hedera helix* L.	Asteraceae	BRG
171	*Hedypnois rhagadioloides* (L.) F.W.Schmidt	Asteraceae	DUT
172	*Hedysarum coronarium* L.	Fabaceae	HAS
173	*Hedysarum spinosissimum* L.	Fabaceae	SCG
174	*Heliotropium europaeum* L.	Boraginaceae	SCG
175	*Hippocrepis ciliata* Willd.	Fabaceae	DUT
176	*Hippocrepis multisiliquosa* L.	Fabaceae	SCG
177	*Hippocrepis unisiliquosa* L.	Fabaceae	SCG
178	*Hirschfeldia incana* (L.) Lagr.-Foss.	Brassicaceae	SCG
179	Hordeum murinum subsp. leporinum (Link) Arcang.	Poaceae	SCG
180	*Hordeum vulgare* L.	Poaceae	BRG
181	Hornungia procumbens subsp. revelierei (Jord.) Giardina & Raimondo	Brassicaceae	DUT ^2^
182	*Hyoscyamus albus* L.	Solanaceae	SCG
183	*Hyoseris frutescens* L.	Asteraceae	BRG
184	*Hyoseris radiata* L.	Asteraceae	HAS
185	*Hyoseris scabra* L.	Asteraceae	DUT
186	*Hyparrhenia hirta* (L.) Stapf	Poaceae	SCG ^2^
187	*Hypericum aegypticum* L.	Hypericaceae	SCG
188	*Hypericum pubescens* Boiss.	Hypericaceae	BRG
189	*Hypericum triquetrifolium* Turra	Hypericaceae	BRG
190	*Hypochaeris achyrophorus* L.	Asteraceae	DUT
191	*Iris sicula* Tod.	Iridaceae	CAS
192	*Juncus acutus* L.	Juncaceae	SCG
193	*Juncus hybridus* Brot.	Juncaceae	SCG
194	*Juncus maritimus* Lam.	Juncaceae	SCG
195	*Kickxia spuria* (L.) Dumort.	Plantaginaceae	BRG
196	*Kundmannia sicula* DC.	Apiaceae	DUT ^2^
197	*Lactuca serriola* L.	Asteraceae	DUT
198	*Lagurus ovatus* L.	Poaceae	DUT
199	*Lamium amplexicaule* L.	Lamiaceae	BRG
200	*Lathyrus articulatus* L.	Fabaceae	BRG
201	*Lathyrus clymenum* L.	Fabaceae	SCG
202	*Lathyrus ochrus* (L.) DC.	Fabaceae	BRG
203	*Leontodon tuberosus* L.	Lamiaceae	SCG
204	*Limbarda crithmoides* (L.) Dumort.	Asteraceae	DUT
206	*Limonium melitense* Brullo	Plumbaginaceae	S&S
207	*Limonium virgatum* (Willd.) Fourr.	Plumbaginaceae	SCG
208	*Limonium zeraphae* Brullo	Plumbaginaceae	SCG
209	*Linaria pseudolaxiflora* Lojac.	Plantaginaceae	DUT
210	*Linum bienne* Mill.	Linaceae	LNF
211	*Linum strictum* L.	Linaceae	DUT
212	*Linum trigynum* L.	Linaceae	DUT
213	*Lobularia maritima* (L.) Desv.	Brassicaceae	SCG
214	*Lolium rigidum* Gaudin	Poaceae	SCG
215	*Lolium temulentum* L.	Poaceae	BRG
216	*Lotus cytisoides* L.	Fabaceae	DUT
217	*Lotus edulis* L.	Fabaceae	SCG
218	*Lotus halophilus* Boiss. & Spruner	Fabaceae	DUT
220	*Lotus ornithopodioides* L.	Fabaceae	SCG
219	*Lygeum spartum* Loefl. ex L.	Poaceae	SCG ^2^
221	*Lysimachia arvensis* (L.) U.Manns & Anderb.	Primulaceae	DUT
222	*Malva arborea* (L.) Webb & Berthel.	Malvaceae	S&S
223	*Malva cretica* Cav.	Malvaceae	DUT
224	*Malva multiflora* (Cav.) Soldano, Banfi & Galasso	Malvaceae	HAS
225	*Malva parviflora* L.	Malvaceae	SCG
226	*Malva setigera* K.F.Schimp. & Spenn.	Malvaceae	DUT
227	Matthiola incana subsp. melitensis Brullo, Lanfr., Pavone & Ronsisv.	Brassicaceae	S&S
228	*Medicago littoralis* Rohde ex Loisel.	Fabaceae	SCG
229	*Medicago marina* L.	Fabaceae	SCG
230	*Medicago minima* (L.) L.	Fabaceae	DUT
231	*Medicago orbicularis* (L.) Bartal.	Fabaceae	SCG
232	*Medicago polymorpha* L.	Fabaceae	SCG
233	*Medicago scutellata* (L.) Mill.	Fabaceae	SCG
234	*Medicago truncatula* Gaertn.	Fabaceae	SCG
235	*Melica ciliata* L.	Poaceae	BRG
236	*Melilotus indicus* (L.) All.	Fabaceae	SCG
237	*Melilotus messanensis* (L.) All.	Fabaceae	SCG
238	*Melilotus segetalis* (Brot.) Ser.	Fabaceae	DUT
239	*Melilotus sulcatus* Desf.	Fabaceae	SCG
240	*Mentha pulegium* L.	Lamiaceae	SCG
241	*Mercurialis annua* L.	Euphorbiaceae	SCG
242	*Mesembryanthemum crystallinum* L.	Aizoaceae	DUT
243	*Mesembryanthemum nodiflorum* L.	Aizoaceae	SCG - Cominotto
244	*Micromeria microphylla* Benth.	Lamiaceae	DUT
245	*Misopates orontium* (L.) Raf.	Plantaginaceae	SCG ^2^
246	*Moraea sisyrinchium* Ker Gawl.	Iridaceae	SCG
247	*Muscari comosum* (L.) Mill.	Asparagaceae	SCG ^2^
248	*Narcissus serotinus* L.	Amaryllidaceae	BRG
249	*Narcissus tazetta* L.	Amaryllidaceae	SCG
250	*Neatostema apulum* (L.) I.M.Johnst.	Boraginaceae	SCG
251	*Neotinea lactea* (Poir.) R.M.Bateman, Pridgeon & M.W.Chase	Orchidaceae	SCG ^2^
252	*Nerium oleander* L.	Apocynaceae	CAM
253	*Nicotiana glauca* Graham	Solanaceae	BRG
254	*Nigella damascena* L.	Ranunculaceae	SCG
255	*Olea europaea* L. (as olive trees)	Oleaceae	CAM
256	*Ononis mitissima* L.	Fabaceae	SCG
257	*Ononis ramosissima* Dsf.	Fabaceae	DUT
258	*Ononis ornithopodioides* L.	Fabaceae	DUT
259	*Ononis reclinata* L.	Fabaceae	DUT
260	*Ononis sieberi* Besser ex DC.	Fabaceae	SCG
261	*Onopordum argolicum* Boiss.	Asteraceae	SCG
262	*Ophrys bombyliflora* Link	Orchidaceae	SCG
263	*Ophrys caesiella* P.Delforge	Orchidaceae	BAR
264	*Ophrys fusca* Link	Orchidaceae	SCG ^2^
265	*Ophrys melitensis* (Salk.) Devillers-Tersch. & Devillers	Orchidaceae	BAR
266	*Ophrys mesaritica* Paulus, C.Alibertis & A.Alibertis	Orchidaceae	BAR
267	*Ophrys speculum* Link	Orchidaceae	BRG
268	*Opuntia ficus-indica* (L.) Mill.	Cactaceae	BRG
269	*Ornithogalum arabicum* L.	Asparagaceae	SCG
270	*Ornithogalum narbonense* L.	Asparagaceae	SCG ^2^
271	*Orobanche cernua* Loefl.	Orobanchaceae	SCG ^2^
272	*Orobanche crenata* Forssk.	Orobanchaceae	BRG
273	*Orobanche mutelii* s.l. F.W.Schultz	Orobanchaceae	SCG
274	*Orobanche minor* Sm.	Orobanchaceae	BRG
275	*Orobanche picridis-hieracioides* Holandre	Orobanchaceae	SCG
276	*Orobanche pubescens* d’Urv.	Orobanchaceae	SCG ^2^
277	*Oxalis pes-caprae* L.	Oxalidaceae	SCG
278	*Pallenis spinosa* (L.) Cass.	Asteraceae	DUT
279	*Pancratium maritimum* L.	Amaryllidaceae	SCG
280	*Papaver rhoeas* L.	Papaveraceae	SCG
281	Papaver somniferum subsp. setigerum (DC.) Arcang.	Papaveraceae	BRG
282	*Parapholis incurva* (L.) C.E.Hubb.	Poaceae	DUT
283	*Parietaria judaica* L.	Urticaceae	SCG
284	*Periploca angustifolia* Labill.	Apocynaceae	LNF
285	Phagnalon graecum subsp. ginzbergeri Pignatti	Asteraceae	SCG
286	*Phagnalon rupestre* (L.) DC.	Asteraceae	HAS
287	*Phalaris minor* Retz.	Poaceae	SCG
288	*Phalaris paradoxa* L.	Poaceae	BRG
289	*Phoenix dactylifera* L.	Arecaceae	HAS
290	*Phragmites australis* (Cav.) Trin. ex Steud.	Poaceae	SCG
291	*Picris echioides* L.	Asteraceae	SCG
292	*Pinus halepensis* Mill.	Pinaceae	CAM
293	*Piptatherum miliaceum* Coss.	Poaceae	BRG
294	*Pistacia lentiscus* L.	Anacardiaceae	S&S
295	*Plantago afra* L.	Plantaginaceae	BRG
296	*Plantago coronopus* s.l. L.	Plantaginaceae	DUT
297	*Plantago lagopus* L.	Plantaginaceae	SCG
298	*Plantago serraria* L.	Plantaginaceae	SCG
299	*Plantago weldenii* Rchb.	Plantaginaceae	SCG
300	*Poa annua* L.	Poaceae	BRG
301	*Poa bulbosa* L.	Poaceae	BRG
302	Polycarpon tetraphyllum subsp. alsinifolium (Biv.) Arcang.	Caryophyllaceae	DUT
303	Polycarpon tetraphyllum subsp. diphyllum (Cav.) O.Bolòs & Font Quer	Caryophyllaceae	LNF
304	*Polycarpon tetraphyllum* (L.) L.	Caryophyllaceae	SCG
305	*Polygonum aviculare* L.	Polygonaceae	SCG ^2^
306	*Polygonum maritimum* L.	Polygonaceae	SCG
307	*Polypogon maritimus* Willd.	Poaceae	SCG
308	*Portulaca oleracea* L.	Portulacaceae	BRG
309	*Prasium majus* L.	Lamiaceae	SCG ^2^
310	*Prospero autumnale* complex (L.) Speta	Asparagaceae	SCG ^2^
311	*Prunus domestica* L.	Rosaceae	BRG
312	*Prunus dulcis* D.A.Webb	Rosaceae	BRG
313	*Prunus persica* (L.) Batsch	Rosaceae	BRG
314	*Pseudorlaya pumila* Grande	Apiaceae	BRG
315	*Plocama calabrica* (L.f.) M.Backlund & Thulin	Rubiaceae	HAS
316	*Ranunculus baudotii* Godr.	Ranunculaceae	BRG
317	*Ranunculus bullatus* L.	Ranunculaceae	SCG
318	*Ranunculus muricatus* L.	Ranunculaceae	BRG
319	*Raphanus raphanistrum* L.	Brassicaceae	SCG ^2^
320	*Rapistrum rugosum* (L.) All.	Brassicaceae	BRG
321	*Reichardia picroides* (L.) Roth	Asteraceae	SCG
322	*Reseda alba* L.	Resedaceae	SCG
323	*Reseda lutea* L.	Resedaceae	SCG
324	*Rhamnus oleoides* L.	Rhamnaceae	BRG
325	*Rhodalsine geniculata* Williams	Caryophyllaceae	DUT ^2^
326	*Ricinus communis* L.	Euphorbiaceae	CAM
327	*Ridolfia segetum* Moris	Apiaceae	BRG
328	*Romulea columnae* Sebast. & Mauri	Iridaceae	SCG
329	*Romulea variicolor* Mifsud	Iridaceae	SCG ^2^
330	*Rostraria cristata* (L.) Tzvelev	Poaceae	DUT
331	*Rubus ulmifolius* Schott	Rosaceae	BRG
332	*Rumex bucephalophorus* L.	Polygonaceae	SCG
333	*Rumex conglomeratus* Murray	Polygonaceae	SCG
334	*Ruta chalepensis* L.	Rutaceae	DUT
335	*Sagina apetala* Ard.	Caryophyllaceae	BRG
336	*Sagina maritima* Don	Caryophyllaceae	SCG
337	*Sagina procumbens* L.	Caryophyllaceae	HAS
338	*Salsola kali* L.	Amaranthaceae	SCG ^2^
339	*Salsola melitensis* Botsch.	Amaranthaceae	S&S
340	*Salsola soda* L.	Amaranthaceae	SCG ^2^
341	*Salvia verbenaca* L.	Lamiaceae	SCG
342	*Samolus valerandi* L.	Primulaceae	SCG
343	Sanguisorba minor subsp. verrucosa (Ehrenb. ex Decne.) Holmboe	Rosaceae	SCG
344	*Scabiosa atropurpurea* L.	Caprifoliaceae	SCG ^2^
345	*Scandix pecten-veneris* L.	Apiaceae	SCG ^2^
346	*Scilla sicula* Tineo ex Guss.	Asparagaceae	LNF
347	*Scolymus grandiflorus* Desf.	Asteraceae	SCG
348	*Scolymus hispanicus* L.	Asteraceae	SCG
349	*Scolymus maculatus* L.	Asteraceae	BRG
350	*Scorpiurus muricatus* L.	Fabaceae	DUT
351	*Scrophularia peregrina* L.	Scrophulariaceae	SCG
352	*Sedum caeruleum* L.	Crassulaceae	SCG
353	*Sedum litoreum* Guss.	Crassulaceae	DUT
354	*Sedum rubens* L.	Crassulaceae	SCG
355	*Sedum sediforme* (Jacq.) Pau	Crassulaceae	BRG
356	*Senecio bicolor* (Willd.) B.Nord. & Greuter	Asteraceae	DUT
357	*Senecio leucanthemifolius* Poir.	Asteraceae	DUT ^2^
358	*Senecio pygmaeus* DC.	Asteraceae	DUT
359	*Senecio vulgaris* L.	Asteraceae	BRG
360	*Serapias parviflora* Parl.	Orchidaceae	SCG
361	*Setaria verticillata* (L.) P.Beauv.	Poaceae	HAS
362	*Sherardia arvensis* L.	Rubiaceae	SCG
363	*Sideritis romana* L.	Lamiaceae	SCG
364	*Sinapis arvensis* L.	Brassicaceae	SCG
365	*Silene colorata* Poir.	Caryophyllaceae	SCG ^2^
366	*Silene nocturna* L.	Caryophyllaceae	BRG
367	*Silene sedoides* Poir.	Caryophyllaceae	DUT
368	*Silene vulgaris* (Moench) Garcke	Caryophyllaceae	SCG
369	*Sisymbrium officinale* (L.) Scop.	Brassicaceae	SCG ^2^
370	*Smyrnium olusatrum* L.	Apiaceae	BRG
371	*Solanum nigrum* L.	Solanaceae	SCG
372	*Sonchus asper* (L.) Hill	Asteraceae	SCG
373	*Sonchus oleraceus* L.	Asteraceae	DUT
374	*Sonchus tenerrimus* L.	Asteraceae	BRG
375	*Spergularia rubra* J.Presl & C.Presl	Caryophyllaceae	SCG
376	*Sporobolus pungens* (Schreb.) Kunth	Poaceae	BRG
377	*Stachys ocymastrum* Briq.	Lamiaceae	SCG ^2^
378	*Stellaria media* (L.) Vill.	Caryophyllaceae	HAS
379	*Stellaria neglecta* (Lej.) Weihe	Caryophyllaceae	BRG
380	*Stellaria pallida* (Dumort.) Crép.	Caryophyllaceae	BRG
381	*Stipa capensis* Thunb.	Poaceae	DUT
382	*Suaeda vera* Forssk. ex J.F.Gmel.	Amaranthaceae	SCG ^2^
383	*Tamarix africana* Poir.	Tamaricaceae	DUT ^2^
384	*Tamarix gallica* L.	Tamaricaceae	HAS
385	*Tetragonolobus purpureus* L.	Fabaceae	SCG
386	*Teucrium flavum* L.	Lamiaceae	BRG
387	*Teucrium fruticans* L.	Lamiaceae	DUT
388	*Theligonum cynocrambe* L.	Rubiaceae	SCG
389	*Thesium humile* Vahl	Santalaceae	BRG
390	*Thymbra capitata* (L.) Cav.	Lamiaceae	SCG
391	*Tordylium apulum* L.	Apiaceae	SCG
392	*Trifolium campestre* Schreb.	Fabaceae	SCG
393	*Trifolium nigrescens* Viv.	Fabaceae	SCG
394	*Trifolium repens* L.	Fabaceae	SCG
395	*Trifolium resupinatum* L.	Fabaceae	DUT
396	*Trifolium scabrum* L.	Fabaceae	DUT
397	*Trifolium stellatum* L.	Fabaceae	DUT
398	*Trifolium tomentosum* L.	Fabaceae	SCG
399	*Trigonella monspeliaca* L.	Fabaceae	DUT
400	*Tripodion tetraphyllum* (L.) Fourr.	Fabaceae	SCG
401	*Trisetaria aurea* (Ten.) Pignatti ex Kerguélen	Poaceae	BRG
402	*Triticum aestivum* L.	Poaceae	BRG
403	*Triticum durum* (Desf.) Husn.	Poaceae	BRG
404	*Urospermum picroides* (L.) Scop. ex F.W.Schmidt	Asteraceae	SCG
405	*Urtica membranacea* Poir. ex Savigny	Urticaceae	SCG
406	*Urtica pilulifera* L.	Urticaceae	BRG
407	*Urtica urens* L.	Urticaceae	BRG
408	*Valantia hispida* L.	Rubiaceae	DUT
409	*Valantia muralis* L.	Rubiaceae	DUT
410	*Verbascum sinuatum* L.	Scrophulariaceae	SCG
411	*Verbena officinalis* L.	Verbenaceae	SCG ^2^
412	Vicia sativa subsp. nigra Ehrh.	Fabaceae	BRG
413	*Vicia faba* L.	Fabaceae	HAS
414	Vicia sativa subsp. sativa L.	Fabaceae	SCG ^2^
415	*Vitis vinifera* L.	Vitaceae	BRG ^1^
416	*Vitex agnus-castus* L.	Lamiaceae	SCG
417	*Vulpia ciliata* Dumort.	Poaceae	SCG
418	*Vulpia fasciculata* Forssk.	Poaceae	SCG
419	*Vulpia membranacea* (L.) Dumort.	Poaceae	BRG
420	*Zannichellia melitensis* Brullo, Giusso & Lanfr.	Potamogetonaceae	SCG

**Table 4.** Plants recorded from the Comino archipelago during the 26 surveys conducted in 21 visits between 2019 and 2025 – specifically on 3 February 2019 (survey S1), 30–31 March 2019 (S2), 4–7 May 2019 (S3), 31 August 2019 (S4), 19 October 2019 (S5), 4 June 2020 (S6a, 6b), 11 October 2020 (S7), 1 November 2020 (S8), 20 March 2021 (S9), 26 March 2021 (S10), 11 April 2021 (S11), 15 April 2021 (S12a–12c), 25 April 2021 (S13), 4 May 2021 (S14), 21 November 2021 (S15), 15 March 2022 (S16), 19 April 2022 (S17), 27 May 2022 (S18), 1 May 2023 (S19), 26 November 2024 (S20a–20c), and 4 April 2025 (S21), and the estimated frequencies on the Comino archipelago and specifically on Cominotto Island and the four main islets of il-Ħaġra ta’ Taħt il-Mazz [Ħ.Mazz], il-Ħaġra l-Kbira [Ħ.Kbira] and il-Ħaġra ż-Żgħira [Ħ.Żgħira], using the following legend: (RR) very rare; (R) rare, (I) infrequent/scarce, fragmented or frequent in a few places, (C) frequent-common, (CC) very common throughout. The table incorporates observations from preliminary surveys conducted by Pavon and Mifsud in 2008 and Médail in 2018 (unpublished material). The table also includes the species in bold type that are new records for Comino. See methodology for further details.

	Species recorded between 2019 and 2025	Pre-liminary study: Pavon and Mifsud 2008 (unpbl.)	Pre-liminary study: Médail 2018 (unpbl.)	Survey no. when first observed on Comino archipelago and its [frequency]	Survey no. when first observed on Cominotto and its [frequency	Survey no. when first observed on Ħ. Mazz and its [frequency	Survey no. when first observed on Ħ. Kbira and its [frequency	Survey no. when first observed on Ħ. Żgħira and its [frequency	Present on Cominotto or the islets but absent or very rare on Comino Is.
1	*Acacia saligna* s.l. (incl. *A. cyanophylla* & *A. pycnantha)*	x	x	S1 [R]	.	.	.	.	
2	*Adiantum capillus-veneris*			S2 [RR]	.	.	.	.	
3	*Aeonium arboreum*			S2 [RR]	.	.	.	.	
4	*Aetheorhiza bulbosa* (*= Sonchus bulbosus*)			S2 [R]	S10 [R]	.	.	.	
5	Agave americana var. americana		x	S1 [R]	S6a [RR]	.	.	.	
6	*Agave sisalana*			S6 [RR]	S6a [RR]	.	.	.	✓
7	*Agave attenuata*			S9 [RR]	.	.	.	.	
8	*Ailanthus altissima*	x	x	S1 [R]	.	.	.	.	
9	*Ajuga iva*	x	x	S5 [I]	S7 [R]	.	.	.	
10	*Allium lojaconoi*	x		S1 [R]	S6a [RR]	.	S14 [I]	.	
11	*Allium polyanthum*			S2 [RR]	S10 [R]	.	S20a [RR]	.	
12	*Allium roseum*			S3 [RR]	.	.	.	.	
13	*Aloe ciliaris*			S9 [RR]	.	.	.	.	
14	*Althea hirsuta* (= *Malva setigera*)			S3 [RR]	S21 [RR]	.	.	.	
15	Anacamptis coriophora subsp. fragrans			S2 [RR]	S21 [RR]	.	.	.	
16	Anacamptis pyramidalis subsp. pyramidalis	x		S2 [R]	S12a [RR]	.	.	.	
17	*Anchusa italica*			S18 [RR]	.	.	.	.	
18	*Anredera cordifolia*			S9 [RR]	.	.	.	.	
19	*Anthemis urvilleana*			S7 [R]	S7 [R]	.	S14 [CC]	S12c [I]	✓
20	Anthyllis hermanniae subsp. melitensis	x	x	S1 [CC]	S6a [CC]	S6b [RR]	.	.	
21	Anthyllis vulneraria subsp. maura			S2 [R]	S12a [R]	.	S14 [R]	.	
22	*Aptenia lancifolia*			S2 [RR]	.	.	.	.	
23	*Arisarum vulgare*		x	S1 [I]	S7 [I]	.	S20a [I]	.	
24	*Arthrocaulon macrostachyum*			S1 [R]	.	.	S14 [RR]	S12c [CC]	
25	*Arum italicum*	x		S1 [R]	.	.	.	.	
26	*Arundo donax*	x	x	S1 [R]	.	.	.	.	
27	*Asparagus aphyllus*	x	x	S1 [R]	S6a [R]	S6b [RR]	S14 [R]	S6b [RR]	
28	Asperula aristata subsp. scabra	x		S19 [RR]	.	.	.	.	
29	*Asphodelus fistulosus*			S18 [RR]	.	.	.	.	
30	*Asphodelus ramosus*	x	x	S1 [C]	.	.	.	.	
31	*Asteriscus aquaticus*	x	x	S1 [CC]	S6a [I]	.	.	.	
32	*Astragalus boeticus*			S1 [RR]	.	.	.	.	
33	*Astragalus hamosus*			S9 [RR]	.	.	.	.	
34	*Atractylis gummifera*	x		S1 [I]	S6a [I]	.	.	.	
35	*Atriplex halimus*			S1 [RR]	.	.	.	.	
36	*Atriplex prostrata*			S8 [RR]	.	.	.	.	
37	*Avena barbata*			S17 [RR]	.	.	.	.	
38	*Bellardia trixago*			S2 [I]	.	.	.	.	
39	*Bellis annua*		x	S1 [R]	.	.	.	.	
40	Beta vulgaris subsp. maritima		x	S1 [RR]	.	.	.	.	
41	*Bituminaria bituminosa*			S3 [RR]	S12a [R]	.	.	.	
42	*Blackstonia acuminata*	x		S2 [I]	S6a [R]	S12b [RR]	S14 [R]	.	
43	*Borago officinalis*	x		S1 [R]	.	.	.	.	
44	*Bougainvillae spectabilis*			S2 [RR]	.	.	.	.	
45	*Brachypodium hybridum*			S2 [R]	S10 [R]	S6b [R]	.	.	
46	*Brachypodium retusum*	x	x	S1 [R]	S6a [I]	.	.	.	
47	Brassica rapa subsp. sylvestris			S2 [RR]	.	.	.	.	
48	*Briza minor*			S3 [RR]	.	.	.	.	
49	*Bromus alopecuros*			S17 [RR]	.	.	.	.	
50	*Bromus fasciculatus*			S1 [I]	S21 [R]	S12b [R]	S14 [RR]	.	
51	*Bromus madritensis*			S3 [I]	S12a [RR]	.	.	.	
52	*Bromus rigidus*			S12 [RR]	S12a [RR]	.	.	.	✓
53	*Cakile maritima*			S1 [RR]	.	.	.	.	
54	*Campanula erinus*			S2 [RR]	.	.	.	.	
55	*Capparis orientalis* (= C. spinosa subsp. rupestris)	x	x	S1 [I]	S6a [R]	S6b [RR]	S14 [R]	S6b [R]	
56	*Carlina involucrata*		x	S1 [I]	S7 [CC]	.	.	.	
57	*Carpobrotus aff. acinaciformis*	x		S1 [R]	.	.	.	.	
58	*Carthamus lanatus*			S7 [RR]	S7 [RR]	.	.	.	✓
59	*Chasmanthe floribunda*			S18 [RR]	.	.	.	.	
60	*Catapodium marinum*		x	S1 [RR]	S21 [R]	S6b [RR]	.	.	
61	*Catapodium pauciflorum*			S11 [R]	S12a [R]	S12b [R]	S14 [R]	.	
62	*Catapodium rigidum*			S2 [I]	S10 [RR]	S12b [RR]	S14 [I]	.	
63	Catapodium rigidum subsp. major			S13 [RR]	.	.	.	.	
64	*Catapodium zwierleinii*			S2 [RR]	.	.	.	.	
65	*Centaurea diluta*			S18 [RR]	.	.	.	.	
66	*Centaurea melitensis*			S1 [R]	.	.	.	.	
67	*Centaurium erythraea*	x	x	S1 [I]	S6a [R]	S12b [R]	S14 [R]	.	
68	*Centaurium pulchellum*			S2 [C]	S12a [RR]	.	S14 [R]	.	
69	*Centaurium spicatum*			S8 [RR]	.	.	.	.	
70	*Centaurium tenuiflorum*			S3 [I]	S6a [RR]	S12b [RR]	.	.	
71	*Centranthus ruber*			S17 [RR]	.	.	.	.	
72	*Ceratonia siliqua*	x	x	S1 [R]	.	.	.	.	
73	*Chamaerops humilis*			S9 [RR]	.	.	.	.	
74	*Chenopodium murale*			S2 [R]	S7 [R]	.	.	.	
75	*Chiliadenus bocconei*			S2 [R]	.	.	.	.	
76	*Chrozophora tinctoria*			S4 [R]	.	.	.	.	
77	*Cichorium spinosum*			S7 [RR]	S7 [RR]	.	.	.	✓
78	*Citrus limon*			S13 [RR]	.	.	.	.	
79	*Colchicum cupanii*		x	S1 [R]	.	.	.	.	
80	*Convolvulus althaeoides*		x	S3 [R]	S7 [RR]	.	.	.	
81	*Convolvulus arvensis*			S3 [R]	.	.	.	.	
82	*Convolvulus elegantissimus*			S19 [RR]	.	.	.	.	
83	*Convolvulus lineatus*			S3 [RR]	.	.	.	.	
84	*Convolvulus oleifolius*		x	S1 [I]	S10 [RR]	.	S14 [CC]	.	
85	*Convolvulus siculus*			S2 [RR]	.	.	.	.	
86	*Coronilla scorpioides*			S10 [R]	S10 [R]	.	.	.	✓
87	*Crithmum maritimum*		x	S1 [R]	S6a [R]	.	.	.	
88	*Crucianella maritima*			S6 [R]	S6a [R]	S6b [R]	S14 [C]	.	✓
89	*Cupressus sempervirens*			S1 [I]	.	.	.	.	
90	*Cuscuta epithymum*			S1 [R]	S10 [R]	.	S14 [R]	.	
91	*Cydonia oblonga*			S3 [RR]	.	.	.	.	
92	*Cynara cardunculus*	x	x	S1 [I]	S6a [R]	.	.	.	
93	*Cynodon dactylon*	x		S1 [RR]	.	.	.	.	
94	*Daucus carota*			S13 [RR]	.	.	.	.	
95	Daucus carota subsp. commutatus var. tenuisectus			S7 [R]	S7 [R]	.	.	.	✓
96	*Daucus lopadusanus*			S2 [R]	.	.	.	.	
97	*Daucus rupestris* s.l.			S1 [I]	S6a [R]	S6b [CC]	S14 [C]	S6b [I]	
98	*Desmazeria pignatti*			S1 [I]	S10 [I]	S6b [R]	.	S6b [RR]	
99	*Diplotaxis erucoides*		x	S9 [RR]	.	.	.	.	
100	*Diplotaxis viminea*			S13 [RR]	.	.	.	.	
101	*Dittrichia graveolens*		x	S4 [RR]	.	.	.	.	
102	*Dittrichia viscosa*	x	x	S1 [I]	S6a [R]	.	.	.	
103	*Drimia pancration*	x	x	S1 [R]	S6a [C]	S6b [R]	S14 [I]	S6b [I]	
104	*Ecballium elaterium*	x	x	S1 [R]	.	.	.	.	
105	*Echium arenarium*			S2 [R]	S10 [R]	.	.	.	
106	*Echium parviflorum*			S1 [RR]	S7 [RR]	.	.	.	
107	*Emex spinosa*			S11 [RR]	.	.	.	.	
108	*Erica multiflora*	x		S1 [I]	.	.	.	.	
109	*Eriobotrya japonica*			S2 [RR]	.	.	.	.	
110	*Erodium cicutarium*	x	x	S1 [R]	.	.	.	.	
111	Erodium cicutarium subsp. salzmannii			S3 [RR]	.	.	.	.	
112	*Erodium malacoides*			S1 [I]	S10 [RR]	.	.	.	
113	*Eucalyptus gomphocephala*			S1 [R]	.	.	.	.	
114	*Euphorbia dendroides*		x	S17 [RR]	.	.	.	.	
115	*Euphorbia exigua*			S2 [I]	S10 [C]	.	.	.	
116	*Euphorbia hypericifolia*			S5 [RR]	.	.	.	.	
117	*Euphorbia melitensis*	x	x	S1 [CC]	S6a [I]	.	.	.	
118	*Euphorbia peplus*		x	S1 [I]	S7 [I]	.	.	.	
119	*Euphorbia pinea*	x	x	S1 [C]	S6a [C]	.	S14 [R]	.	
120	*Evax pygmaea*			S1 [R]	S21 [R]	.	.	.	
121	*Ferula melitensis*	x	x	S1 [C]	S6a [I]	.	.	.	
122	*Ficus carica*	x	x	S1 [R]	S6a [RR]	.	.	.	
123	*Filago pyramidata* s.l.			S10 [R]	S10 [RR]	.	.	.	✓
124	*Foeniculum vulgare* s.l.			S3 [RR]	.	.	.	.	
125	*Frankenia hirsuta*			S3 [R]	S7 [RR]	.	.	S12c [R]	
126	*Frankenia pulverulenta*	x		S1 [R]	S21 [RR]	.	.	.	
127	*Fumaria parviflora*			S13 [RR]	.	.	.	.	
128	*Galactites tomentosus*	x		S1 [R]	S10 [RR]	.	.	.	
129	*Galium murale*			S1 [I]	S7 [R]	.	.	.	
130	*Gladiolus communis* s.l.			S17 [RR]	.	.	.	.	
131	*Glaucium flavum*			S2 [RR]	.	.	.	.	
132	*Glebionis coronarium*			S11 [RR]	.	.	.	.	
133	*Hedypnois cretica*			S2 [RR]	.	.	.	.	
134	*Hedypnois rhagadioloides*			S3 [RR]	S12a [RR]	.	.	.	
135	*Hedysarum coronarium*			S3 [RR]	.	.	.	.	
136	*Hedysarum spinosissimum*			S2 [R]	S10 [RR]	.	.	.	
137	*Heliotropium europaeum*			S4 [R]	.	.	.	.	
138	*Hippocrepis ciliata*			S2 [RR]	.	.	.	.	
139	*Hippocrepis multisiliquosa*			S2 [R]	S10 [RR]	.	.	.	
140	*Hippocrepis unisiliquosa*			S2 [RR]	S10 [I]	.	.	.	
141	*Hirschfeldia incana*			S2 [RR]	.	.	.	.	
142	Hornungia procumbens subsp. revelierei			S21	S21 [RR]	.	.	.	
143	*Hylocereus undatus*			S11 [RR]	.	.	.	.	
144	*Hyoscyamus albus*			S1 [R]	.	.	.	.	
145	*Hyoseris scabra*			S1 [R]	.	.	.	.	
146	*Hyparrhenia hirta*	x	x	S3 [RR]	.	.	.	.	
147	*Hypericum aegypticum*	x	x	S1 [I]	S6a [RR]	.	S14 [CC]	.	
148	*Hypericum triquetrifolium*			S13 [R]	.	.	.	.	
149	*Hypochaeris achyrophorus*			S3 [RR]	.	.	S14 [R]	.	
150	Jacobaea maritima subsp. sicula	x	x	S1 [I]	S6a [I]	.	S14 [RR]	.	
151	*Juncus acutus*			S18 [RR]	.	.	.	.	
152	*Juncus hybridus*			S2 [R]	.	.	S14 [RR]	.	
153	*Lactuca serriola*			S16 [RR]	.	.	.	.	
154	*Lagurus ovatus*	x		S2 [I]	.	.	.	.	
155	*Lantana camara*			S2 [RR]	.	.	.	.	
156	*Laurus nobilis*			S2 [RR]	.	.	.	.	
157	*Lavandula dentata*			S15 [RR]	.	.	.	.	
158	*Leontodon tuberosus*			S1 [R]	S20c [RR]	.	.	.	
159	*Limbarda crithmoides*	x	x	S1 [I]	S6a [I]	S6b [CC]	S14 [I]	S6b [I]	
160	*Limonium melitense*	x		S1 [I]	S6a [R]	S6b [R]	S14 [R]	S6b [I]	
161	*Limonium virgatum*		x	S1 [I]	S6a [I]	S6b [C]	S14 [C]	S6b [C]	
162	*Limonium zeraphae*			S3 [RR]	.	.	.	.	
163	*Linaria pseudolaxiflora*			S1 [R]	S10 [RR]	.	.	.	
164	*Linum strictum*			S2 [R]	S12a [R]	.	S14 [R]	.	
165	*Linum trigynum*	x		S2 [I]	S10 [RR]	.	S14 [I]	.	
166	*Lobularia maritima*		x	S5 [RR]	S10 [RR]	.	.	.	
167	*Lolium rigidum*			S3 [RR]	.	.	.	.	
168	*Lonicera implexa*	x	x	S1 [R]	.	.	.	.	
169	*Lotus cytisoides*		x	S3 [R]	S10 [RR]	.	.	.	
170	*Lotus edulis*			S3 [RR]	S12a [R]	S12b [RR]	S20a [RR]	.	
171	*Lotus halophilus*			S11 [RR]	.	.	.	.	
172	*Lotus ornithopodioides*	x		S3 [R]	.	.	.	.	
173	*Lygeum spartum*			S12 [RR]	.	.	.	S12c [R]	✓
174	*Lysimachia arvensis*		x	S1 [C]	S6a [R]	.	S20a [RR]	.	
175	*Lysimachia loeflingii*	x	x	S1 [RR]	S6a [C]	.	.	.	
176	*Lythrum hyssopifolia*	x		S17 [R]	.	.	.	.	
177	*Malus pumila*			S2 [RR]	.	.	.	.	
178	*Malva cretica*			S1 [RR]	.	.	.	.	
179	*Malva multiflora*			S3 [RR]	.	.	.	.	
180	*Malva nicaeensis*			S18 [RR]	.	.	.	.	
181	*Malva parviflora*			S1 [R]	.	.	.	.	
182	Matthiola incana subsp. melitensis			S6 [RR]	.	S6b [I]	.	.	✓
183	*Medicago littoralis*			S10 [RR]	S10 [RR]	.	.	.	✓
184	*Medicago polymorpha*			S3 [RR]	.	S12b [RR]	.	.	
185	*Melilotus indicus*			S10 [RR]	S10 [I]	.	S14 [R]	S12c [I]	✓
186	*Melilotus segetalis*			S3 [RR]	.	.	.	.	
187	*Mentha pulegium*		x	S3 [R]	.	.	.	.	
188	*Mercurialis annua*	x	x	S1 [R]	.	.	.	.	
189	*Mesembryanthemum nodiflorum*			S1 [I]	S6a [R]	S12b [RR]	S14 [C]	S12c [C]	
190	*Micromeria microphylla*			S1 [R]	S20c [RR]	.	.	.	
191	*Mirabilis jalapa*			S15 [RR]	.	.	.	.	
192	*Moraea sisyrinchium*			S2 [R]	.	.	.	.	
193	*Morus nigra*			S18 [RR]	.	.	.	.	
194	*Musa × paradisiaca*			S15 [RR]	.	.	.	.	
195	*Muscari comosum*			S9 [RR]	S10 [RR]	.	.	.	
196	*Narcissus deficiens*			S5 [RR]	S7 [RR]	.	S20a [RR]	.	
197	*Narcissus tazetta* s.l.		x	S1 [C]	S10 [R]	.	S14 [I]	.	
198	*Nerium oleander*			S1 [R]	.	.	.	.	
199	*Nicotiana glauca*	x	x	S1 [RR]	.	.	.	.	
200	*Nigella damascena*			S2 [R]	.	.	.	.	
201	Olea europaea var. europaea	x	x	S1 [R]	.	.	.	.	
202	Olea europaea var. silvestris			S3 [R]	S20c [RR]	.	.	.	
203	*Ononis mitissima*			S17 [RR]	.	.	.	.	
204	Ononis natrix subsp. ramosissima	x	x	S1 [I]	.	.	.	.	
205	*Ononis ornithopodioides*			S2 [RR]	.	.	.	.	
206	*Ononis reclinata*			S3 [RR]	S21 [RR]	.	S14 [R]	.	
207	Ononis viscosa subsp. sieberi			S10 [RR]	S10 [R]	.	.	.	✓
208	*Ophrys bombyliflora*			S2 [R]	.	.	.	.	
209	*Ophrys caesiella (incl. O. × tumentia)*			S16 [RR]	.	.	.	.	
210	Ophrys iricolor subsp. mesaritica			S17 [RR]	.	.	.	.	
211	*Ophrys melitensis*			S21 [RR]	S21 [RR]	.	.	.	✓
212	*Ophrys speculum*			S21 [RR]	S21 [RR]	.	.	.	✓
213	*Opuntia ficus-indica*			S1 [R]	.	.	.	.	
214	*Opuntia microdasys*			S11 [RR]	.	.	.	.	
215	*Opuntia vulgaris* s.l.			S13 [RR]	.	.	.	.	
216	*Ornithogalum arabicum*			S3 [RR]	.	.	.	.	
217	*Ornithogalum narbonense*			S1 [R]	.	.	.	.	
218	*Orobanche cernua*			S11 [R]	S12a [RR]	S12b [RR]	S14 [RR]	.	
219	*Orobanche crenata*			S3 [RR]	.	.	.	.	
220	Orobanche gr. ramosa			S1 [I]	.	.	.	.	
221	*Orobanche minor* ?			S12 [RR]	S12a [RR]	.	.	.	✓
222	*Orobanche pubescens*			S3 [RR]	.	.	.	.	
223	*Oxalis pes-caprae*		x	S1 [I]	.	.	.	.	
224	Oxalis pes-caprae var. pleniflora		x	S1 [R]	S10 [RR]	.	.	.	
225	*Pallenis spinosa*			S3 [RR]	S12a [RR]	.	.	.	
226	*Pancratium maritimum*	x		S1 [RR]	.	.	.	.	
227	*Papaver rhoeas*			S13 [RR]	.	.	.	.	
228	Papaver somniferum subsp. setigerum			S9 [RR]	.	.	.	.	
229	*Parapholis incurva*			S6 [R]	S21 [RR]	S12b [RR]	S14 [RR]	S12c [I]	✓
230	*Parietaria judaica*			S13 [R]	.	.	.	.	
231	*Pelargonium cucullatum*			S2 [RR]	.	.	.	.	
232	*Pelargonium × hybridum*			S1 [RR]	.	.	.	.	
233	*Periploca angustifolia*	x	x	S1 [I]	S6a [R]	.	.	.	
234	Phagnalon graecum subsp. ginzbergeri			S13 [RR]	.	.	S14 [RR]	.	
235	*Phagnalon rupestre*			S3 [R]	S10 [RR]	.	.	.	
236	*Phalaris minor*			S3 [RR]	.	.	.	.	
237	*Phoenix canariensis*			S1 [R]	.	.	.	.	
238	*Phragmites australis*	x		S1 [RR]	.	.	.	.	
239	*Pinus halepensis*	x	x	S1 [I]	.	.	.	.	
240	*Pistacia lentiscus*	x	x	S1 [CC]	S6a [I]	S6b [RR]	S20a [RR]	.	
241	*Pittosporum tobira*			S2 [R]	.	.	.	.	
242	*Plantago coronopus*			S1 [R]	.	.	.	.	
243	*Plantago lagopus*			S1 [RR]	.	.	.	.	
244	*Plantago weldenii*			S19 [R]	.	.	.	.	
245	*Polycarpon tetraphyllum*			S2 [RR]	.	.	.	.	
246	*Polygonum maritimum*			S1 [RR]	.	.	.	.	
247	*Polypogon maritimus*	x		S2 [C]	S21 [R]	S12b [R]	S14 [R]	.	
248	*Polypogon monspeliensis*			S21	S21 [RR]	.	.	.	✓
249	*Polypogon subspathaceus*	x		S2 [C]	.	S12b [I]	S14 [R]	.	
250	*Prasium majus*			S2 [R]	S10 [RR]	.	.	.	
251	Prospero aggr. autumnalis		x	S5 [I]	S7 [I]	.	S20a [R]	.	
252	*Prunus dulcis*			S1 [R]	.	.	.	.	
253	*Prunus insititia*			S2 [RR]	.	.	.	.	
254	*Prunus persica*			S15 [RR]	.	.	.	.	
255	*Punica granatum*			S1 [R]	.	.	.	.	
256	*Pyrus communis*			S3 [RR]	.	.	.	.	
257	*Ranunculus bullatus*			S15 [RR]	.	.	.	.	
258	*Reichardia picroides*		x	S1 [R]	S10 [I]	.	S20a [RR]	.	
259	*Reseda alba*			S2 [RR]	.	.	.	.	
260	*Rhamnus oleoides*			S3 [RR]	.	.	.	.	
261	*Ricinus communis*			S3 [RR]	.	.	.	.	
262	*Romulea variicolor*			S1 [R]	S21 [RR]	.	S14 [I]	.	
263	*Rosmarinus officinalis*			S2 [RR]	.	.	.	.	
264	*Rostraria cristata*	x		S2 [R]	.	S6b [RR]	S14 [RR]	.	
265	*Rumex bucephalophorus*			S1 [R]	.	.	.	.	
266	*Rumex conglomeratus*			S1 [RR]	.	.	.	.	
267	*Ruppia maritima*			S18 [RR]	.	.	.	.	
268	*Ruta chalepensis*	x	x	S1 [I]	S6a [I]	.	.	.	
269	*Sagina apetala*			S3 [RR]	.	.	.	.	
270	*Sagina maritima*			S1 [RR]	.	.	.	.	
271	*Sagina procumbens*			S21	S21 [R]	.	.	.	✓
272	*Salsola melitensis*		x	S1 [I]	S6a [R]	S6b [I]	.	S6b [R]	
273	*Samolus valerandi*			S16 [RR]	.	.	.	.	
274	Sanguisorba minor subsp. verrucosa	x		S3 [I]	.	.	.	.	
275	*Schoenus nigricans*			S10 [RR]	S10 [RR]	.	.	.	✓
276	*Scorpiurus muricatus*	x		S2 [I]	S10 [I]	.	S20a [RR]	.	
277	*Scrophularia peregrina*		x	S9 [RR]	.	.	.	.	
278	*Sedum adolphii*			S18 [RR]	.	.	.	.	
279	*Sedum caeruleum*	x		S1 [I]	.	.	.	.	
280	*Sedum litoreum*			S2 [R]	S10 [R]	.	S14 [I]	.	
281	*Sedum rubens*		x	S1 [RR]	S10 [RR]	.	.	.	
282	*Sedum sediforme*	x		S1 [C]	.	.	.	.	
283	*Senecio leucanthemifolius*		x	S1 [RR]	S10 [RR]	S6b [R]	S20a [RR]	S6b [R]	✓
284	*Senecio pygmaeus*		x	S1 [R]	S20c [R]	.	.	.	
285	*Serapias parviflora*			S18 [RR]	.	.	.	.	
286	*Setaria adhaerens*			S2 [RR]	.	.	.	.	
287	*Sideritis romana*			S1 [R]	S10 [RR]	.	.	.	
288	*Sinapis cf. alba*			S1 [RR]	S10 [RR]	.	.	.	
289	*Sinapis arvensis*			S15 [RR]	.	.	.	.	
290	*Silene sedoides*	x		S1 [I]	S10 [I]	S6b [R]	S14 [I]	S6b [R]	
291	*Sisymbrium officinale*			S2 [RR]	.	.	.	.	
292	*Solanum nigrum*			S1 [RR]	.	.	.	.	
293	*Sonchus asper* s.l.			S9 [RR]	.	.	.	.	
294	*Sonchus oleraceus* s. str.		x	S1 [R]	S10 [R]	.	S20a [RR]	S20b [RR]	
295	*Sonchus tenerrimus*			S1 [RR]	S20c	.	.	.	
296	*Spergularia diandra*			S11 [RR]	.	.	S14 [R]	S12c [R]	
297	*Sporobolus pungens*			S7 [RR]	S6a [RR]	.	.	.	✓
298	*Tamarix africana*			S1 [R]	.	.	.	.	
299	*Tamarix gallica*		x	S1 [RR]	.	.	.	.	
300	*Tetraclinis articulata*		x	S2 [RR]	.	.	.	.	
301	*Teucrium flavum*	x	x	S1 [I]	S6a [R]	.	.	.	
302	*Teucrium fruticans*	x	x	S1 [C]	S7 [C]	.	.	.	
303	*Theligonum cynocrambe*			S10 [RR]	S10 [R]	.	.	.	✓
304	*Thymbra capitata*	x	x	S1 [C]	S6a [I]	.	.	.	
305	*Tordylium apulum*			S2 [R]	S10 [I]	.	.	.	
306	*Trifolium campestre*			S11 [RR]	.	.	.	.	
307	*Trifolium nigrescens*			S2 [R]	.	.	.	.	
308	*Trifolium scabrum*			S10 [R]	S10 [R]	.	.	.	✓
309	*Trifolium suffocatum*			S9 [RR]	.	.	.	.	
310	*Trigonella monspeliaca*			S2 [RR]	.	.	.	.	
311	*Tripodion tetraphyllum*	x		S2 [RR]	.	.	.	.	
312	*Triticum durum*			S10 [RR]	S10 [RR]	.	.	.	✓
313	*Typha domingensis*			S8 [RR]	.	.	.	.	
314	*Umbilicus rupestris*			S1 [RR]	.	.	.	.	
315	*Urospermum picroides*	x		S1 [R]	S21 [RR]	.	.	.	
316	*Urtica membranacea*			S1 [R]	.	.	.	.	
317	*Urtica urens*		x	S2 [R]	.	.	.	.	
318	*Valantia muralis*		x	S1 [C]	S10 [CC]	S6b [RR]	S14 [I]	.	
319	*Verbascum sinuatum*	x	x	S1 [R]	.	.	.	.	
320	Vicia sativa subsp. sativa			S3 [RR]	.	.	.	.	
321	*Vitis vinifera*			S3 [RR]	.	.	.	.	
322	*Vitex agnus-castus*			S1 [RR]	.	.	.	.	
323	*Vulpia ciliata*	x		S8 [I]	S10 [R]	.	.	.	
324	*Vulpia fasciculata*			S11 [RR]	.	.	.	.	
325	*Washingtonia filifera*			S1 [RR]	.	.	.	.	
326	*Yucca gigantea*			S9 [RR]	.	.	.	.	
327	*Zannichellia melitensis*		x	S1 [RR]	.	.	.	.	
328	*Zantedeschia aethiopica*			S3 [RR]	.	.	.	.	
	Total no. of spp.:			328	131	32	53	20	

**Table 5.** Checklist of vascular plant species recorded on the Comino archipelago, whether documented in past records or observed during this study (!), sorted by family name. The table provides whether the taxon is (denoted by Y) a new record for Comino (NRc / bold type), whether it was previously recorded in historical literature but not observed in this study (= lost record, LRc), whether it occurs specifically on Cominotto (CTO), Ħaġra l-Kbira (ĦK), Ħaġra ż-Żgħira (ĦZ), and Ħaġra ta’ Taħt il-Mazz (ĦM), and its Raunkiaer’s life form and status for the Maltese Islands.

No.	Family	Species recorded on Comino [! = observed in this study] [bold = new record for Comino]	Life form	Status for Malta	New rec	Lost rec	CTO	ĦK	ĦŻ	ĦM
1	Aizoaceae	*Carpobrotus acinaciformis* (L.) L.Bolus !	Ch	Inv. Alien	.	.	.	.	.	.
2	Aizoaceae	*Mesembryanthemum crystallinum* L.	Th	Native	.	Y	.	.	.	.
3	Aizoaceae	*Mesembryanthemum lancifolium* (L.Bolus) Klak !	Ch	Inv. Alien	Y	.	.	.	.	.
4	Aizoaceae	*Mesembryanthemum nodiflorum* L. !	Th	Native	.	.	Y	Y	Y	Y
5	Amaranthaceae	*Arthrocaulon macrostachyum* (Moric.) Piirainen & G.Kadereit !	nPh	Native	.	.	.	Y	Y	.
6	Amaranthaceae	*Atriplex halimus* L. !	nPh	Native	Y	.	.	.	.	.
7	Amaranthaceae	*Atriplex prostrata* Boucher ex DC. !	H	Native	Y	.	.	.	.	.
8	Amaranthaceae	*Beta vulgaris* L. s.l.!	H	Native	.	.	.	.	.	.
9	Amaranthaceae	*Chenopodiastrum murale* L. !	Th	Native	.	.	Y	.	.	.
10	Amaranthaceae	*Salsola kali* L.	Ch	Native	.	Y	Y	.	Y	Y
11	Amaranthaceae	*Salsola melitensis* Botsch. !	Th	Native [E]	.	.	.	.	.	.
12	Amaranthaceae	*Salsola soda* L.	Th	Native	.	Y	.	.	.	.
13	Amaranthaceae	*Suaeda vera* Forssk. ex J.F.Gmel.	nPh	Native	.	Y	.	.	.	.
14	Amaryllidaceae	*Allium cepa* L.	G	Alien	.	Y	.	.	.	.
15	Amaryllidaceae	*Allium lojaconoi* Brullo, Lanfr. & Pavone !	G	Native [SE]	.	.	Y	Y	.	.
16	Amaryllidaceae	*Allium polyanthum* Schult. & Schult.f. !	G	Native	.	.	Y	Y	.	.
17	Amaryllidaceae	*Allium roseum* Krock. !	G	Native	.	.	.	.	.	.
18	Amaryllidaceae	*Allium sativum* L.	G	Alien	.	Y	.	.	.	.
19	Amaryllidaceae	*Allium subvillosum* Salzm. ex Schult. & Schult.f.	G	Native	.	Y	.	.	.	.
20	Amaryllidaceae	*Allium trifoliatum* Cirillo	G	Native	.	Y	.	.	.	.
21	Amaryllidaceae	*Narcissus deficiens* Herb. !	G	Native	.	.	Y	Y	.	.
22	Amaryllidaceae	*Narcissus tazetta* L. s.l.!	G	Native	.	.	Y	Y	.	.
23	Amaryllidaceae	*Pancratium maritimum* L. !	G	Native	.	.	.	.	.	.
24	Anacardiaceae	*Pistacia lentiscus* L. !	Ph	Native	.	.	Y	Y	.	Y
25	Apiaceae	*Ammi majus* L.	Th	Native	.	Y	.	.	.	.
26	Apiaceae	*Ammoides pusilla* (Brot.) Breistr.	Th	Native	.	Y	.	.	.	.
27	Apiaceae	*Bupleurum lancifolium* Hornem.	Th	Native	.	Y	.	.	.	.
28	Apiaceae	*Crithmum maritimum* L. !	Ch	Native	.	.	Y	.	.	.
29	Apiaceae	*Cuminum cyminum* L.	Th	Alien	.	Y	.	.	.	.
30	Apiaceae	*Daucus carota* L. !	Ch	Native	Y	.	.	.	.	.
31	Apiaceae	*Daucus carota* L. subsp. commutatus var. tenuisectus (Degen ex Palyi) Reduron !	Th	Native	Y	.	Y	.	.	.
32	Apiaceae	*Daucus carota* L. subsp. drepanensis (Arcang.) Heywood	Ch	Native	.	Y	.	.	.	.
33	Apiaceae	*Daucus carota* L. subsp. rupestris (Guss.) Heywood !	H	Native [SE]	.	.	Y	Y	Y	Y
34	Apiaceae	*Daucus lopadusanus* Tineo !	H	Native [SE]	.	.	.	.	.	.
35	Apiaceae	*Daucus pumilus* Ball	Th	Native	.	Y	.	.	.	.
36	Apiaceae	*Eryngium maritimum* L.	G	Native	.	Y	.	.	.	.
37	Apiaceae	*Ferula melitensis* Brullo, C.Brullo, Cambria, Giusso, Salmeri & Bacch. !	H	Native [E]	.	.	Y	.	.	.
38	Apiaceae	*Foeniculum vulgare* Mill. !	H	Native	.	.	.	.	.	.
39	Apiaceae	*Kundmannia sicula* DC.	Th	Native	.	Y	.	.	.	.
40	Apiaceae	*Ridolfia segetum* Moris	Th	Native	.	Y	.	.	.	.
41	Apiaceae	*Scandix pecten-veneris* L.	Th	Native	.	Y	.	.	.	.
42	Apiaceae	*Smyrnium olusatrum* L.	H	Native	.	Y	.	.	.	.
43	Apiaceae	*Tordylium apulum* L. !	Th	Native	.	.	Y	.	.	.
44	Apocynaceae	*Nerium oleander* L. !	Ph	Re-introduced	.	.	.	.	.	.
45	Apocynaceae	*Periploca angustifolia* Labill. !	nPh	Native	.	.	Y	.	.	.
46	Araceae	*Arisarum vulgare* O.Targ.Tozz. !	G	Native	.	.	Y	Y	.	.
47	Araceae	Arum italicum subsp. italicum Mill. !	G	Native	.	.	.	.	.	.
48	Araceae	*Washingtonia filifera* (Rafarin) H.Wendl. ex de Bary !	Ph	Alien	Y	.	.	.	.	.
49	Araceae	*Zantedeschia aethiopica* (L.) Spreng. !	G	Alien	Y	.	.	.	.	.
50	Arecaceae	*Chamaerops humilis* L. subsp. humilis !	Ph	Native	Y	.	.	.	.	.
51	Arecaceae	*Phoenix canariensis* Chabaud !	Ph	Alien	Y	.	.	.	.	.
52	Arecaceae	*Phoenix dactylifera* L.	Ph	Native	.	Y	.	.	.	.
53	Asparagaceae	Agave americana subsp. americana L. !	H	Inv. Alien	.	.	Y	.	.	.
54	Asparagaceae	*Agave attenuata* Salm-Dyck !	Ph	Alien	Y	.	.	.	.	.
55	Asparagaceae	*Agave sisalana* Perine !	H	Inv. Alien	Y	.	Y	.	.	.
56	Asparagaceae	*Asparagus aphyllus* L. !	Ch	Native	.	.	Y	Y	Y	Y
57	Asparagaceae	*Drimia pancration* (Steinh.) J.C.Manning & Goldblatt !	G	Native	.	.	Y	Y	Y	Y
58	Asparagaceae	*Leopoldia comosa* (L.) Parl. !	G	Native	.	.	Y	.	.	.
59	Asparagaceae	*Ornithogalum arabicum* L. !	G	Native	.	.	.	.	.	.
60	Asparagaceae	*Ornithogalum narbonense* L. !	G	Native	.	.	.	.	.	.
61	Asparagaceae	*Prospero autumnale* s.l. (L.) Speta !	G	Native	.	.	Y	Y	.	.
62	Asparagaceae	*Scilla sicula* Tineo ex Guss.	G	Native [SE]	.	y	.	.	.	.
63	Asparagaceae	*Yucca gigantea* Lem. !	Ph	Alien	Y	.	.	.	.	.
64	Asphodelaceae	*Aloe vera* (L.) Burm.f.	Ch	Alien	.	Y	.	.	.	.
65	Asphodelaceae	*Aloiampelos ciliaris* Klopper & Gideon F.Sm. !	Ch	Alien	Y	.	.	.	.	.
66	Asphodelaceae	*Asphodelus fistulosus* L. !	G	Native	Y	.	.	.	.	.
67	Asphodelaceae	*Asphodelus ramosus* L. !	G	Native	.	.	.	.	.	.
68	Asteraceae	*Ambrosia maritima* L.	Th	Native	.	Y	.	.	.	.
69	Asteraceae	*Anthemis arvensis* L.	Th	Native	.	Y	.	.	.	.
70	Asteraceae	*Anthemis tomentosa* Gouan ex Boiss.	Th	Native	.	Y	.	.	.	.
71	Asteraceae	*Anthemis secundiramea* Biv. !	Th	Native	.	.	Y	Y	Y	.
72	Asteraceae	*Asteriscus aquaticus* (L.) Less. !	Th	Native	.	.	Y	.	.	.
73	Asteraceae	*Bellis annua* L. !	Th	Native	.	.	.	.	.	.
74	Asteraceae	*Bellis sylvestris* Cirillo	H	Native	.	Y	.	.	.	.
75	Asteraceae	*Calendula arvensis* L.	Th	Native	.	Y	.	.	.	.
76	Asteraceae	*Calendula suffruticosa* Vahl.	Ch	Native	.	Y	.	.	.	.
77	Asteraceae	*Carduus pycnocephalus* Spreng. subsp. arabicus (Jacq. ex Murray) Nyman	Th	Native	.	Y	.	.	.	.
78	Asteraceae	*Carduus pycnocephalus* Spreng. subsp. pycnocephalus L.	Th	Native	.	Y	.	.	.	.
79	Asteraceae	*Carlina gummifera* Less. !	H	Native	.	.	Y	.	.	.
80	Asteraceae	*Carlina involucrata* Poir. !	H	Native	.	.	Y	.	.	.
81	Asteraceae	*Carthamus lanatus* L. !	Th	Native	Y	.	Y	.	.	.
82	Asteraceae	*Carthamus tinctorius* L.	Th	Alien	.	Y	.	.	.	.
83	Asteraceae	*Centaurea diluta* Aiton !	Th	Alien	Y	.	.	.	.	.
84	Asteraceae	*Centaurea melitensis* L. !	Th	Native	.	.	.	.	.	.
85	Asteraceae	*Centaurea nicaeensis* L.	Th	Native	.	Y	.	.	.	.
86	Asteraceae	*Chiliadenus bocconei* Brullo !	Ch	Native [E]	.	.	.	.	.	.
87	Asteraceae	*Cichorium spinosum* L. !	Ch	Native	.	.	Y	.	.	.
88	Asteraceae	*Cynara cardunculus* L. !	H	Native	.	.	Y	.	.	.
89	Asteraceae	*Dittrichia graveolens* (L.) Greuter !	Th	Native	.	.	.	.	.	.
90	Asteraceae	*Dittrichia viscosa* (L.) Greuter subsp. viscosa!	Ch	Native	.	.	Y	.	.	.
91	Asteraceae	*Erigeron bonariensis* L.	Th	Alien	.	Y	.	.	.	.
92	Asteraceae	*Filago pygmaea* Cav. !	Th	Native	.	.	Y	.	.	.
93	Asteraceae	*Filago pyramidata* s.l. L. !	Th	Native	.	.	Y	.	.	.
94	Asteraceae	*Galactites tomentosus* Moench !	Th	Native	.	.	Y	.	.	.
95	Asteraceae	*Glebionis coronarium* (L.) Cass. ex Spach !	Th	Native	.	.	.	.	.	.
96	Asteraceae	*Hedera helix* L.	Ph	Native	.	Y	.	.	.	.
97	Asteraceae	*Hedypnois cretica* !	Th	Native	Y	.	.	.	.	.
98	Asteraceae	*Hedypnois rhagadioloides* (L.) F.W.Schmidt !	Th	Native	.	.	Y	.	.	.
99	Asteraceae	*Helminthotheca echioides* (L.) Holub	Th	Native	.	Y	.	.	.	.
100	Asteraceae	*Hyoseris frutescens* Brullo & Pavone	Ch	Native [E]	.	Y	.	.	.	.
101	Asteraceae	*Hyoseris radiata* L.	H	Native	.	Y	.	.	.	.
102	Asteraceae	*Hyoseris scabra* L. !	Th	Native	.	.	.	.	.	.
103	Asteraceae	*Hypochaeris achyrophorus* L. !	Th	Native	.	.	.	Y	.	.
104	Asteraceae	*Jacobaea maritima* (L.) Pelser & Meijden subsp. sicula N.G.Passal., Peruzzi & Pellegrino !	Ch	Native [SE]	.	.	Y	Y	.	.
105	Asteraceae	*Lactuca serriola* L. !	Th	Native	.	.	.	.	.	.
106	Asteraceae	*Limbarda crithmoides* (L.) Dumort. !	Ch	Native	.	.	Y	Y	Y	Y
107	Asteraceae	*Onopordum tauricum* Willd.	H	Native	.	Y	.	.	.	.
108	Asteraceae	*Pallenis spinosa* (L.) Cass. !	H	Native	.	.	Y	.	.	.
109	Asteraceae	*Phagnalon rupestre* (L.) DC. subsp. graecum Batt. !	Ch	Native	.	.	.	Y	.	.
110	Asteraceae	Phagnalon rupestre subsp. rupestre (L.) DC. !	Ch	Native	.	.	Y	.	.	.
111	Asteraceae	*Reichardia picroides* (L.) Roth !	Ch	Native	.	.	Y	Y	.	.
112	Asteraceae	*Scolymus grandiflorus* Desf.	H	Native	.	Y	.	.	.	.
113	Asteraceae	*Scolymus hispanicus* L.	H	Native	.	Y	.	.	.	.
114	Asteraceae	*Scolymus maculatus* L.	Th	Native	.	Y	.	.	.	.
115	Asteraceae	*Senecio leucanthemifolius* Poir. !	Th	Native	.	.	Y	Y	Y	Y
116	Asteraceae	*Senecio pygmaeus* DC. !	Th	Native [SE]	.	.	Y	.	.	.
117	Asteraceae	*Senecio vulgaris* L.	Th	Native	.	Y	.	.	.	.
118	Asteraceae	*Sonchus asper* (L.) Hill s.l.!	Th	Native	.	.	.	.	.	.
119	Asteraceae	*Sonchus bulbosus* (L.) N.Kilian & Greuter !	G	Native	.	.	Y	.	.	.
120	Asteraceae	*Sonchus oleraceus* L. !	Th	Native	.	.	Y	Y	Y	.
121	Asteraceae	*Sonchus tenerrimus* L. !	Th	Native	.	.	.	.	.	.
122	Asteraceae	*Urospermum picroides* (L.) Scop. ex F.W.Schmidt !	Th	Native	.	.	Y	.	.	.
123	Basellaceae	*Anredera cordifolia* (Ten.) Steenis !	nPh	Alien	Y	.	.	.	.	.
124	Boraginaceae	*Anchusa azurea* Mill. !	H	Native	.	.	.	.	.	.
125	Boraginaceae	*Borago officinalis* L. !	Th	Native	.	.	.	.	.	.
126	Boraginaceae	*Cynoglossum creticum* Mill.	Th	Native	.	Y	.	.	.	.
127	Boraginaceae	*Echium arenarium* Guss. !	H	Native	.	.	Y	.	.	.
128	Boraginaceae	*Echium parviflorum* Moench !	Th	Native	.	.	Y	.	.	.
129	Boraginaceae	*Heliotropium europaeum* L. !	Th	Native	.	.	.	.	.	.
130	Boraginaceae	*Neatostema apulum* (L.) I.M.Johnst.	Th	Native	.	Y	.	.	.	.
131	Brassicaceae	*Biscutella didyma* L.	Th	Native	.	Y	.	.	.	.
132	Brassicaceae	*Biscutella lyrata* L.	Th	Native	.	Y	.	.	.	.
133	Brassicaceae	*Brassica oleracea* s.l. L.	Th	Alien	.	Y	.	.	.	.
134	Brassicaceae	*Brassica rapa* L. !	Th	Native	Y	.	.	.	.	.
135	Brassicaceae	*Brassica rupestris* s.l. Raf.	Th	Native	.	Y	.	.	.	.
136	Brassicaceae	*Cakile maritima* Scop. !	Th	Native	.	.	.	.	.	.
137	Brassicaceae	*Capsella bursa-pastoris* Medik.	Th	Native	.	Y	.	.	.	.
138	Brassicaceae	*Diplotaxis erucoides* (L.) DC. !	Th	Native	.	.	.	.	.	.
139	Brassicaceae	*Diplotaxis viminea* DC. !	Th	Native	.	.	.	.	.	.
140	Brassicaceae	*Draba verna* L.	Th	Native	.	Y	.	.	.	.
141	Brassicaceae	*Eruca vesicaria* (L.) Cav.	Th	Alien	.	Y	.	.	.	.
142	Brassicaceae	*Hirschfeldia incana* (L.) Lagr.-Foss. !	Th	Native	.	.	.	.	.	.
143	Brassicaceae	*Hornungia procumbens* (L.) Hayek subsp. revelierei (Jord.) Giardina & Raimondo	Th	Native	.	.	Y	.	.	.
144	Brassicaceae	*Lobularia maritima* (L.) Desv. !	Ch	Native	.	.	Y	.	.	.
145	Brassicaceae	*Matthiola incana* (L.) W.T.Aiton subsp. melitensis Brullo, Lanfr., Pavone & Ronsisv. !	Ch	Native [E]	.	.	.	.	.	Y
146	Brassicaceae	*Raphanus raphanistrum* L.	Th	Native	.	Y	.	.	.	.
147	Brassicaceae	*Rapistrum rugosum* (L.) All.	Th	Native	.	Y	.	.	.	.
148	Brassicaceae	*Sinapis alba* L. !	Th	Native	Y	.	Y	.	.	.
149	Brassicaceae	*Sinapis arvensis* L. !	Th	Native	.	.	.	.	.	.
150	Brassicaceae	*Sisymbrium officinale* (L.) Scop. !	Th	Native	.	.	.	.	.	.
151	Cactaceae	*Opuntia ficus-indica* (L.) Mill. !	Ph	Inv. Alien	.	.	.	.	.	.
152	Cactaceae	*Opuntia microdasys* (Lehm.) Pfeiff. !	nPh	Alien	Y	.	.	.	.	.
153	Cactaceae	*Opuntia stricta* (Haw.) Haw. !	Ph	Alien	Y	.	.	.	.	.
154	Cactaceae	*Selenicereus undatus* (Haw.) D.R.Hunt !	Ph	Alien	Y	.	.	.	.	.
155	Campanulaceae	*Campanula erinus* L. !	Th	Native	.	.	.	.	.	.
156	Capparidaceae	*Capparis spinosa* L. subsp. rupestris (Sm.) Nyman	nPh	Native	.	.	Y	Y	Y	Y
157	Caprifoliaceae	*Lonicera implexa* Aiton !	Ph	Native	Y	.	.	.	.	.
158	Caprifoliaceae	*Scabiosa atropurpurea* L.	Th	Native	.	Y	.	.	.	.
159	Caprifoliaceae	*Valeriana graciliflora* (Fisch. & C.A.Mey.) Byng & Christenh.	Th	Native	.	Y	.	.	.	.
160	Caprifoliaceae	*Valeriana rubra* L. !	Ch	Native	Y	.	.	.	.	.
161	Caryophyllaceae	*Cerastium glomeratum* Thuill.	Th	Native	.	Y	.	.	.	.
162	Caryophyllaceae	*Minuartia geniculata* Thell.	Th	Native	.	Y	.	.	.	.
163	Caryophyllaceae	*Polycarpon tetraphyllum* (L.) L. subsp. alsinifolium (Biv.) Arcang.	Th	Native	.	Y	.	.	.	.
164	Caryophyllaceae	*Polycarpon tetraphyllum* (L.) L. subsp. diphyllum (Cav.) O.Bolòs & Font Quer	Th	Native	.	y	.	.	.	.
165	Caryophyllaceae	*Polycarpon tetraphyllum* subsp. tetraphyllum (L.) L. !	Th	Native	.	.	.	.	.	.
166	Caryophyllaceae	*Sagina apetala* Ard. !	Th	Native	.	.	.	.	.	.
167	Caryophyllaceae	*Sagina maritima* Don !	Th	Native	.	.	.	.	.	.
168	Caryophyllaceae	*Sagina procumbens* L.	H	Native	.	.	Y	.	.	.
169	Caryophyllaceae	*Silene colorata* Poir.	Th	Native	.	Y	.	.	.	.
170	Caryophyllaceae	*Silene nocturna* L.	Th	Native	.	Y	.	.	.	.
171	Caryophyllaceae	*Silene sedoides* Poir. !	Th	Native	.	.	Y	Y	Y	Y
172	Caryophyllaceae	*Silene vulgaris* (Moench) Garcke	H	Native	.	Y	.	.	.	.
173	Caryophyllaceae	*Spergularia diandra* (Guss.) Heldr. !	Th	Native	Y	.	.	Y	Y	.
174	Caryophyllaceae	*Spergularia rubra* J.Presl & C.Presl	Ch	Native	.	Y	.	.	.	.
175	Caryophyllaceae	*Stellaria media* (L.) Vill.	Th	Native	.	Y	.	.	.	.
176	Caryophyllaceae	*Stellaria neglecta* (Lej.) Weihe	Th	Native	.	Y	.	.	.	.
177	Caryophyllaceae	*Stellaria pallida* (Dumort.) Crép.	Th	Native	.	Y	.	.	.	.
178	Cistaceae	*Fumana thymifolia* Spach	Ch	Native	.	Y	.	.	.	.
179	Colchicaceae	*Colchicum cupanii* s.l. Guss. !	G	Native	.	.	.	.	.	.
180	Convolvulaceae	*Convolvulus althaeoides* L. !	G	Native	.	.	Y	.	.	.
181	Convolvulaceae	*Convolvulus arvensis* L. !	G	Native	.	.	.	.	.	.
182	Convolvulaceae	*Convolvulus elegantissimus* Mill. !	Th	Native	.	.	.	.	.	.
183	Convolvulaceae	*Convolvulus lineatus* L. !	H	Native	.	.	.	.	.	.
184	Convolvulaceae	*Convolvulus oleifolius* Desr. !	Ch	Native	.	.	Y	Y	.	.
185	Convolvulaceae	*Convolvulus pentapetaloides* L.	H	Native	.	Y	.	.	.	.
186	Convolvulaceae	*Convolvulus siculus* L. !	Th	Native	Y	.	.	.	.	.
187	Convolvulaceae	*Cuscuta epithymum* L. !	Th	Native	.	.	Y	Y	.	.
188	Crassulaceae	*Aeonium arboreum* Webb & Berthel. !	nPh	Alien	Y	.	.	.	.	.
189	Crassulaceae	*Crassula vaillantii* (Willd.) Schoenl.	Hy	Native	.	Y	.	.	.	.
190	Crassulaceae	*Petrosedum sediforme* (Jacq.) Grulich !	Ch	Native	.	.	.	.	.	.
191	Crassulaceae	*Sedum adolphii* Raym.-Hamet !	nPh	Alien	Y	.	.	.	.	.
192	Crassulaceae	*Sedum caeruleum* L. !	Th	Native	.	.	.	.	.	.
193	Crassulaceae	*Sedum litoreum* Guss. !	Th	Native	.	.	Y	Y	.	.
194	Crassulaceae	*Sedum rubens* L. !	Th	Native	.	.	Y	.	.	.
195	Crassulaceae	*Umbilicus rupestris* (Salisb.) Dandy !	G	Native	Y	.	.	.	.	.
196	Cucurbitaceae	*Ecballium elaterium* (L.) A.Rich. !	G	Native	.	.	.	.	.	.
197	Cupressaceae	*Cupressus sempervirens* L. !	Ph	Native	Y	.	.	.	.	.
198	Cupressaceae	*Tetraclinis articulata* (Vahl) Mast. !	Ph	Native	Y	.	.	.	.	.
199	Cyperaceae	*Carex divisa* Huds.	G	Native	.	Y	.	.	.	.
200	Cyperaceae	*Schoenus nigricans* L. !	G	Native	Y	.	Y	.	.	.
201	Ericaceae	*Erica multiflora* Huds. !	nPh	Native	Y	.	.	.	.	.
202	Euphorbiaceae	*Chrozophora tinctoria* (L.) A.Juss. !	Th	Native	.	.	.	.	.	.
203	Euphorbiaceae	*Euphorbia dendroides* L. !	nPh	Native	.	.	.	.	.	.
204	Euphorbiaceae	*Euphorbia exigua* L. !	Th	Native	.	.	Y	.	.	.
205	Euphorbiaceae	*Euphorbia hypericifolia* L. !	Ch	Alien	Y	.	.	.	.	.
206	Euphorbiaceae	*Euphorbia melitensis* Parl. !	nPh	Native [E]	.	.	Y	.	.	.
207	Euphorbiaceae	*Euphorbia paralias* L.	Ch	Native	.	Y	.	.	.	.
208	Euphorbiaceae	*Euphorbia peplis* L.	Th	Native	.	y	.	.	.	.
209	Euphorbiaceae	*Euphorbia peplus* L. !	Th	Native	.	.	Y	.	.	.
210	Euphorbiaceae	Euphorbia segetalis var. pinea (L.) Lange !	Ch	Native	.	.	Y	Y	.	.
211	Euphorbiaceae	*Euphorbia terracina* L.	Th	Native	.	Y	.	.	.	.
212	Euphorbiaceae	*Mercurialis annua* L. !	Th	Native	.	.	.	.	.	.
213	Euphorbiaceae	*Ricinus communis* L. !	nPh	Inv. Alien	.	.	.	.	.	.
214	Fabaceae	*Acacia saligna* (Labill.) H.L.Wendl. !	Ph	Inv. Alien	.	.	.	.	.	.
215	Fabaceae	*Anthyllis hermanniae* L. subsp. melitensis Brullo & Giusso !	Ch	Native [E]	.	.	Y	.	.	Y
216	Fabaceae	*Anthyllis vulneraria* L. subsp. maura (Beck) Maire !	H	Native	.	.	Y	Y	.	.
217	Fabaceae	*Astragalus boeticus* L. !	Th	Native	.	.	.	.	.	.
218	Fabaceae	*Astragalus hamosus* L. !	Th	Native	.	.	.	.	.	.
219	Fabaceae	*Astragalus sesameus* L.	Th	Native	.	Y	.	.	.	.
220	Fabaceae	*Bituminaria bituminosa* (L.) C.H.Stirt. !	Th	Native	.	.	Y	.	.	.
221	Fabaceae	*Ceratonia siliqua* L. !	Ph	Archaeophyte	.	.	.	.	.	.
222	Fabaceae	*Coronilla scorpioides* (L.) Koch !	Th	Native	.	.	Y	.	.	.
223	Fabaceae	*Hippocrepis ciliata* Willd. !	Th	Native	.	.	.	.	.	.
224	Fabaceae	*Hippocrepis multisiliquosa* L. !	Th	Native	.	.	Y	.	.	.
225	Fabaceae	*Hippocrepis unisiliquosa* L. !	Th	Native	.	.	Y	.	.	.
226	Fabaceae	*Lathyrus clymenum* L.	Th	Native	.	Y	.	.	.	.
227	Fabaceae	Lathyrus clymenum var. articulatus (L.) Arcang.	Th	Native	.	Y	.	.	.	.
228	Fabaceae	*Lathyrus ochrus* (L.) DC.	Th	Native	.	Y	.	.	.	.
229	Fabaceae	*Lotus cytisoides* L. !	Ch	Native	.	.	Y	.	.	.
230	Fabaceae	*Lotus edulis* L. !	Th	Native	.	.	Y	Y	.	Y
231	Fabaceae	*Lotus halophilus* Boiss. & Spruner !	Th	Native	.	.	.	.	.	.
232	Fabaceae	*Lotus ornithopodioides* L. !	Th	Native	.	.	.	.	.	.
233	Fabaceae	*Lotus tetragonolobus* L.	Th	Native	.	Y	.	.	.	.
234	Fabaceae	*Medicago littoralis* Rohde ex Loisel. !	Th	Native	.	.	Y	.	.	.
235	Fabaceae	*Medicago marina* L.	Ch	Native	.	Y	.	.	.	.
236	Fabaceae	*Medicago minima* (L.) L.	Th	Native	.	Y	.	.	.	.
237	Fabaceae	*Medicago monspeliaca* (L.) Trautv. !	Th	Native	.	.	.	.	.	.
238	Fabaceae	*Medicago orbicularis* (L.) Bartal.	Th	Native	.	Y	.	.	.	.
239	Fabaceae	*Medicago polymorpha* L. !	Th	Native	.	.	.	.	.	Y
240	Fabaceae	*Medicago scutellata* (L.) Mill.	Th	Native	.	Y	.	.	.	.
241	Fabaceae	*Medicago truncatula* Gaertn.	Th	Native	.	Y	.	.	.	.
242	Fabaceae	*Melilotus indicus* (L.) All. !	Th	Native	.	.	Y	Y	Y	.
243	Fabaceae	*Melilotus segetalis* (Brot.) Ser. !	Th	Native	.	.	.	.	.	.
244	Fabaceae	*Melilotus siculus* (Turra) Steud.	Th	Native	.	Y	.	.	.	.
245	Fabaceae	*Melilotus sulcatus* Desf.	Th	Native	.	Y	.	.	.	.
246	Fabaceae	*Ononis mitissima* L. !	Th	Native	.	.	.	.	.	.
247	Fabaceae	*Ononis ornithopodioides* L. !	Th	Native	.	.	.	.	.	.
248	Fabaceae	*Ononis ramosissima* Desf. !	H	Native	.	.	.	.	.	.
249	Fabaceae	*Ononis reclinata* L. !	Th	Native	.	.	Y	Y	.	.
250	Fabaceae	*Ononis sieberi* Besser ex DC. !	Th	Native	.	.	Y	.	.	.
251	Fabaceae	*Scorpiurus muricatus* L. !	Th	Native	.	.	Y	Y	.	.
252	Fabaceae	*Sulla coronaria* (L.) B.H.Choi & H.Ohashi !	H	Native	.	.	.	.	.	.
253	Fabaceae	*Sulla spinosissima* (L.) B.H.Choi & H.Ohashi !	Th	Native	.	.	Y	.	.	.
254	Fabaceae	*Trifolium campestre* Schreb. !	Th	Native	.	.	.	.	.	.
255	Fabaceae	*Trifolium nigrescens* Viv. !	Th	Native	.	.	.	.	.	.
256	Fabaceae	*Trifolium repens* L.	H	Native	.	Y	.	.	.	.
257	Fabaceae	*Trifolium resupinatum* L.	Th	Native	.	Y	.	.	.	.
258	Fabaceae	*Trifolium scabrum* L. !	Th	Native	.	.	Y	.	.	.
259	Fabaceae	*Trifolium stellatum* L.	Th	Native	.	Y	.	.	.	.
260	Fabaceae	*Trifolium suffocatum* L. !	Th	Native	Y	.	.	.	.	.
261	Fabaceae	*Trifolium tomentosum* L.	Th	Native	.	Y	.	.	.	.
262	Fabaceae	*Tripodion tetraphyllum* (L.) Fourr. !	Th	Native	.	.	.	.	.	.
263	Fabaceae	*Vicia ervilia* (L.) Willd.	Th	Alien	.	Y	.	.	.	.
264	Fabaceae	*Vicia faba* L.	Th	Alien	.	Y	.	.	.	.
265	Fabaceae	*Vicia sativa* L. subsp. nigra (L.) Ehrh.	Th	Native	.	Y	.	.	.	.
266	Fabaceae	Vicia sativa subsp. sativa L. !	Th	Native	.	.	.	.	.	.
267	Frankeniaceae	*Frankenia hirsuta* L. !	Ch	Native	.	.	Y	.	Y	.
268	Frankeniaceae	*Frankenia pulverulenta* L. !	Th	Native	.	.	Y	.	.	.
269	Gentianaceae	*Blackstonia acuminata* (W.D.J.Koch & Ziz) Domin !	Th	Native	Y	.	Y	Y	.	Y
270	Gentianaceae	*Centaurium erythraea* Rafn !	H	Native	.	.	Y	Y	.	Y
271	Gentianaceae	*Centaurium pulchellum* (Sw.) Hayek ex Hand.-Mazz., Stadlm., Janch. & Faltis !	Th	Native	.	.	Y	Y	.	.
272	Gentianaceae	*Centaurium tenuiflorum* (Hoffmanns. & Link) Fritsch !	Th	Native	Y	.	Y	.	.	Y
273	Gentianaceae	*Schenkia spiccata* (L.) G.Mans. !	Th	Native	Y	.	.	.	.	.
274	Geraniaceae	*Erodium cicutarium* (L.) L’Hér. !	Th	Native	.	.	.	.	.	.
275	Geraniaceae	*Erodium malacoides* (L.) L’Hér. !	Th	Native	.	.	Y	.	.	.
276	Geraniaceae	*Erodium moschatum* (Burm.f.) L’Hér.	Th	Native	.	Y	.	.	.	.
277	Geraniaceae	*Erodium salzmannii* Delile !	Th	Native	Y	.	.	.	.	.
278	Geraniaceae	*Geranium molle* L.	Th	Native	.	Y	.	.	.	.
279	Geraniaceae	*Geranium robertianum* L.	Th	Native	.	Y	.	.	.	.
280	Geraniaceae	*Geranium rotundifolium* L.	Th	Native	.	Y	.	.	.	.
281	Geraniaceae	*Pelargonium × hortorum* L.H.Bailey !	Ch	Alien	Y	.	.	.	.	.
282	Geraniaceae	*Pelargonium cucullatum* (L.) L’Her. !	Ch	Alien	Y	.	.	.	.	.
283	Hypericaceae	*Hypericum aegypticum* L. !	Ch	Native	.	.	Y	Y	.	.
284	Hypericaceae	*Hypericum pubescens* Boiss.	H	Native	.	Y	.	.	.	.
285	Hypericaceae	*Hypericum triquetrifolium* Turra !	Th	Native	.	.	.	.	.	.
286	Iridaceae	*Chasmanthe floribunda* (Dozy & Molk.) M. Fleisch. !	G	Inv. Alien	Y	.	.	.	.	.
287	Iridaceae	*Gladiolus communis* s.l. L. !	G	Native	Y	.	.	.	.	.
288	Iridaceae	*Gladiolus italicus* Mill.	G	Native	.	Y	.	.	.	.
289	Iridaceae	*Moraea sisyrinchium* Ker Gawl. !	G	Native	.	.	.	.	.	.
290	Iridaceae	*Romulea columnae* Sebast. & Mauri	G	Native	.	Y	.	.	.	.
291	Iridaceae	*Romulea variicolor* Mifsud !	G	Native [SE]	.	.	Y	Y	.	.
292	Juncaceae	*Juncus acutus* L. !	H	Native	.	.	.	.	.	.
293	Juncaceae	*Juncus hybridus* Brot. !	Th	Native	.	.	.	Y	.	.
294	Juncaceae	*Juncus maritimus* Lam.	G	Native	.	Y	.	.	.	.
295	Lamiaceae	*Ajuga iva* (L.) Schreb. !	Ch	Native	.	.	Y	.	.	.
296	Lamiaceae	Clinopodium nepeta subsp. spruneri (Boiss.) Bartolucci & F.Conti	Ch	Native	.	Y	.	.	.	.
297	Lamiaceae	*Lamium amplexicaule* L.	Th	Native	.	Y	.	.	.	.
298	Lamiaceae	*Lavandula dentata* L. !	Ch	Alien	Y	.	.	.	.	.
299	Lamiaceae	*Leontodon tuberosus* L. !	H	Native	.	.	Y	.	.	.
300	Lamiaceae	*Mentha pulegium* L. !	H	Native	.	.	.	.	.	.
301	Lamiaceae	*Micromeria microphylla* Benth. !	Ch	Native	.	.	Y	.	.	.
302	Lamiaceae	*Prasium majus* L. !	Ch	Native	.	.	Y	.	.	.
303	Lamiaceae	*Salvia rosmarinus* L. !	nPh	Native	Y	.	.	.	.	.
304	Lamiaceae	*Salvia verbenaca* L.	H	Native	.	Y	.	.	.	.
305	Lamiaceae	*Sideritis romana* L. !	Th	Native	.	.	Y	.	.	.
306	Lamiaceae	*Stachys ocymastrum* Briq.	Th	Native	.	Y	.	.	.	.
307	Lamiaceae	*Teucrium flavum* L. !	Ch	Native	.	.	Y	.	.	.
308	Lamiaceae	*Teucrium fruticans* L. !	nPh	Native	.	.	Y	.	.	.
309	Lamiaceae	*Thymbra capitata* (L.) Cav. !	Ch	Native	.	.	Y	.	.	.
310	Lamiaceae	*Vitex agnus-castus* L. !	nPh	Native	.	.	.	.	.	.
311	Lauraceae	*Laurus nobilis* L. !	Ph	Native	Y	.	.	.	.	.
312	Linaceae	*Linum bienne* Mill.	H	Native	.	y	.	.	.	.
313	Linaceae	*Linum strictum* L. !	Th	Native	.	.	Y	Y	.	.
314	Linaceae	*Linum trigynum* L. !	Th	Native	.	.	Y	Y	.	.
315	Lythraceae	*Lythrum hyssopifolia* L. !	Th	Native	Y	.	.	.	.	.
316	Lythraceae	*Punica granatum* L. !	nPh	Archaeophyte	Y	.	.	.	.	.
317	Malvaceae	*Malva arborea* (L.) Webb & Berthel.	nPh	Native	.	.	.	.	.	.
318	Malvaceae	*Malva cretica* Cav. !	Th	Native	.	.	.	.	.	.
319	Malvaceae	*Malva multiflora* (Cav.) Soldano, Banfi & Galasso !	Th	Native	.	.	.	.	.	.
320	Malvaceae	*Malva nicaeensis* All. !	Th	Native	Y	.	.	.	.	.
321	Malvaceae	*Malva parviflora* L. !	Th	Native	.	.	.	.	.	.
322	Malvaceae	*Malva setigera* K.F.Schimp. & Spenn. !	Th	Native	.	.	Y	.	.	.
323	Moraceae	*Ficus carica* L. !	Ph	Archaeophyte	.	.	Y	.	.	.
324	Moraceae	*Morus nigra* L. !	Ph	Alien	Y	.	.	.	.	.
325	Musaceae	*Musa × paradisiaca* L. !	Ph	Alien	Y	.	.	.	.	.
326	Myrtaceae	*Eucalyptus gomphocephala* A.Cunn. ex DC. !	Ph	Alien	.	.	.	.	.	.
327	Nyctaginaceae	*Bougainvillae spectabilis* Willd. !	Ph	Alien	Y	.	.	.	.	.
328	Nyctaginaceae	*Mirabilis jalapa* L. !	nPh	Alien	Y	.	.	.	.	.
329	Oleaceae	Olea europaea var. europaea L. !	Ph	Archaeophyte	.	.	.	.	.	.
330	Oleaceae	Olea europaea var. oleaster (Hoffmanns. & Link) A.DC. !	nPh	Native	Y	.	Y	.	.	.
331	Orchidaceae	*Anacamptis collina* Banks & Sol. ex Russell) R.M.Bateman, Pridgeon & M.W.Chase	G	Native	.	y	.	.	.	.
332	Orchidaceae	*Anacamptis coriophora* (L.) R.M.Bateman, Pridgeon & M.W.Chase subsp. fragrans (Pollini) R.M.Bateman, Pridgeon & M.W.Chase !	G	Native	.	.	Y	.	.	.
333	Orchidaceae	Anacamptis pyramidalis subsp. pyramidalis (L.) Rich. !	G	Native	.	.	Y	.	.	.
334	Orchidaceae	Anacamptis pyramidalis subsp. urvilleana (Sommier and Caruana) Landwehr	G	Native [E]	.	y	.	.	.	.
335	Orchidaceae	*Neotinea lactea* (Poir.) R.M.Bateman, Pridgeon & M.W.Chase	G	Native	.	Y	.	.	.	.
336	Orchidaceae	*Ophrys bertolonii* Moretti !	G	Native	Y	y	.	.	.	.
337	Orchidaceae	*Ophrys bombyliflora* Link !	G	Native	.	.	.	.	.	.
338	Orchidaceae	*Ophrys caesiella* (P.Delforge) Kreutz !	G	Native	.	Y	.	.	.	.
339	Orchidaceae	*Ophrys iricolor* Desf. subsp. mesaritica (Paulus, C.Alibertis & A.Alibertis) Kreutz !	G	Native	.	.	.	.	.	.
340	Orchidaceae	*Ophrys melitensis* (Salk.) Devillers-Tersch. & Devillers !	G	Native [E]	.	.	Y	.	.	.
341	Orchidaceae	*Ophrys speculum* Link !	G	Native	.	.	Y	.	.	.
342	Orchidaceae	*Serapias parviflora* Parl. !	G	Native	.	.	.	.	.	.
343	Orobanchaceae	*Bellardia trixago* (L.) All. !	Th	Native	.	.	.	.	.	.
344	Orobanchaceae	*Orobanche cernua* Loefl. !	Th	Native	.	.	Y	Y	.	Y
345	Orobanchaceae	*Orobanche cf. minor* Sm. !	Th	Native	.	.	Y	.	.	.
346	Orobanchaceae	*Orobanche crenata* Forssk. !	Th	Native	.	.	.	.	.	.
347	Orobanchaceae	*Orobanche nana* Noë ex Rchb. !	Th	Native	.	.	.	.	.	.
348	Orobanchaceae	*Orobanche picridis* F.W.Schultz	Th	Native	.	Y	.	.	.	.
349	Orobanchaceae	*Orobanche pubescens* d’Urv. !	Th	Native	.	.	.	.	.	.
350	Oxalidaceae	*Oxalis pes-caprae* L. !	Th	Inv. Alien	.	.	Y	.	.	.
351	Oxalidaceae	Oxalis pes-caprae var. pleniflora (Lowe) Blanco-Dios !	G	Inv. Alien	Y	.	Y	.	.	.
352	Papaveraceae	*Fumaria agraria* Lag.	Th	Native	.	Y	.	.	.	.
353	Papaveraceae	*Fumaria flabellata* Gasp.	Th	Native	.	Y	.	.	.	.
354	Papaveraceae	*Fumaria officinalis* L.	Th	Native	.	Y	.	.	.	.
355	Papaveraceae	*Fumaria parviflora* Lam. !	Th	Native	Y	.	.	.	.	.
356	Papaveraceae	*Glaucium flavum* Crantz !	H	Native	.	.	.	.	.	.
357	Papaveraceae	*Papaver rhoeas* L. !	Th	Native	.	.	.	.	.	.
358	Papaveraceae	*Papaver somniferum* L. subsp. setigerum (DC.) Arcang. !	Th	Native	.	.	.	.	.	.
359	Pinaceae	*Pinus halepensis* Mill. !	Ph	Re-introduced	.	.	.	.	.	.
360	Pittosporaceae	*Pittosporum tobira* W.T.Aiton !	Ph	Alien	Y	.	.	.	.	.
361	Plantaginaceae	*Antirrhinum siculum* Mill.	H	Native	.	Y	.	.	.	.
362	Plantaginaceae	*Antirrhinum tortuosum* Bosc ex Lam.	Ch	Native	.	Y	.	.	.	.
363	Plantaginaceae	*Kickxia spuria* (L.) Dumort.	Th	Native	.	Y	.	.	.	.
364	Plantaginaceae	*Linaria pseudolaxiflora* Lojac. !	Th	Native [SE]	.	.	Y	.	.	.
365	Plantaginaceae	*Misopates orontium* (L.) Raf.	Th	Native	.	Y	.	.	.	.
366	Plantaginaceae	*Plantago afra* L.	Th	Native	.	Y	.	.	.	.
367	Plantaginaceae	*Plantago coronopus* s.l. L. !	Th	Native	.	.	.	.	.	.
368	Plantaginaceae	*Plantago lagopus* L. !	Th	Native	.	.	.	.	.	.
369	Plantaginaceae	*Plantago serraria* L.	H	Native	.	Y	.	.	.	.
370	Plantaginaceae	*Plantago weldenii* Rchb. !	Ch	Native	.	.	.	.	.	.
371	Plumbaginaceae	*Limonium melitense* Brullo !	H	Native [E]	.	.	Y	Y	Y	Y
372	Plumbaginaceae	*Limonium virgatum* (Willd.) Fourr. !	H	Native	.	.	Y	Y	Y	Y
373	Plumbaginaceae	*Limonium zeraphae* Brullo !	H	Native [E]	.	.	.	.	.	.
374	Poaceae	*Aegilops neglecta* Req. ex Bertol.	Th	Native	.	Y	.	.	.	.
375	Poaceae	*Anthoxanthum gracile* Bivon.	Th	Native	.	Y	.	.	.	.
376	Poaceae	*Arundo donax* L. !	G	Inv. Alien (Archaeophyte)	.	.	.	.	.	.
377	Poaceae	*Avena barbata* Pott ex Link !	Th	Native	.	.	.	.	.	.
378	Poaceae	*Avena sterilis* L.	Th	Native	.	Y	.	.	.	.
379	Poaceae	*Brachypodium hybridum* Catalán, Joch.Müll., Hasterok & G.Jenkins !	Th	Native	Y	.	Y	.	.	Y
380	Poaceae	*Brachypodium pinnatum* (L.) P.Beauv.	Th	Native	.	Y	.	.	.	.
381	Poaceae	*Brachypodium retusum* (Pers.) P.Beauv. !	H	Native	.	.	Y	.	.	.
382	Poaceae	*Briza maxima* L.	Th	Native	.	Y	.	.	.	.
383	Poaceae	*Briza minor* L. !	Th	Native	Y	.	.	.	.	.
384	Poaceae	*Bromus alopecuros* Pers. !	Th	Native	Y	.	.	.	.	.
385	Poaceae	*Bromus diandrus* Roth.	Th	Native	.	Y	.	.	.	.
386	Poaceae	*Bromus fasciculatus* C.Presl !	Th	Native	.	.	Y	Y	.	Y
387	Poaceae	*Bromus hordeaceus* s.l. L.	Th	Native	.	Y	.	.	.	.
388	Poaceae	*Bromus madritensis* L. !	Th	Native	.	.	Y	.	.	.
389	Poaceae	*Bromus rigidus* Roth !	Th	Native	.	.	Y	.	.	.
390	Poaceae	*Bromus tectorum* L.	Th	Native	.	Y	.	.	.	.
391	Poaceae	*Catapodium hemipoa* (Delile ex Spreng.) Laínz	Th	Native	.	Y	.	.	.	.
392	Poaceae	*Catapodium marinum* (L.) C.E.Hubb. !	H	Native	.	.	Y	.	.	Y
393	Poaceae	*Catapodium pauciflorum* (Merino) Brullo, Giusso, Miniss. & Spamp. !	Th	Native	Y	.	Y	Y	.	Y
394	Poaceae	*Catapodium rigidum* (L.) C.E.Hubb. !	Th	Native	.	.	Y	Y	.	Y
395	Poaceae	Catapodium rigidum subsp. major (C.Presl) F.H.Perring & P.D.Sell !	Th	Native	Y	.	.	.	.	.
396	Poaceae	*Catapodium zwierleinii* (Lojac.) Brullo !	Th	Native	.	.	.	.	.	.
397	Poaceae	*Coix lacryma-jobi* L.	Th	Alien	.	Y	.	.	.	.
398	Poaceae	*Cynodon dactylon* (L.) Pers. !	H	Native	.	.	.	.	.	.
399	Poaceae	*Dactylis glomerata* L. subsp. hispanica (Roth) Nyman	H	Native	.	Y	.	.	.	.
400	Poaceae	*Desmazeria pignatti* Brullo & Pavone !	Th	Native [SE]	.	.	Y	.	Y	Y
401	Poaceae	*Gastridium ventricosum* (Gouan) Schinz & Thell.	Th	Native	.	Y	.	.	.	.
402	Poaceae	*Hordeum murinum* L. subsp. leporinum (Link) Arcang.	Th	Native	.	Y	.	.	.	.
403	Poaceae	*Hordeum vulgare* L.	Th	Alien	.	Y	.	.	.	.
404	Poaceae	*Hyparrhenia hirta* (L.) Stapf !	H	Native	.	.	.	.	.	.
405	Poaceae	*Lagurus ovatus* L. !	Th	Native	.	.	.	.	.	.
406	Poaceae	*Lolium rigidum* Gaudin !	Th	Native	.	.	.	.	.	.
407	Poaceae	*Lolium temulentum* L.	Th	Native	.	Y	.	.	.	.
408	Poaceae	*Lygeum spartum* Loefl. ex L. !	Ch	Native	.	.	.	.	Y	.
409	Poaceae	*Melica ciliata* L.	Th	Native	.	Y	.	.	.	.
410	Poaceae	*Parapholis incurva* (L.) C.E.Hubb. !	Th	Native	.	.	Y	Y	Y	Y
411	Poaceae	*Phalaris minor* Retz. !	Th	Native	.	.	.	.	.	.
412	Poaceae	*Phalaris paradoxa* L.	Th	Native	.	Y	.	.	.	.
413	Poaceae	*Phragmites australis* (Cav.) Trin. ex Steud. !	G	Native	.	.	.	.	.	.
414	Poaceae	*Piptatherum miliaceum* Coss.	H	Native	.	Y	.	.	.	.
415	Poaceae	*Poa annua* L.	Th	Native	.	Y	.	.	.	.
416	Poaceae	*Poa bulbosa* L.	G	Native	.	Y	.	.	.	.
417	Poaceae	*Polypogon maritimus* Willd. !	Th	Native	.	.	Y	Y	.	Y
418	Poaceae	*Polypogon monspeliensis* (L.) Desf. !	Th	Native	Y	.	Y	.	.	.
419	Poaceae	*Polypogon subspathaceus* Req. !	Th	Native	Y	.	.	Y	.	Y
420	Poaceae	*Rostraria cristata* (L.) Tzvelev !	Th	Native	.	.	.	Y	.	Y
421	Poaceae	*Setaria adhaerens* (Forssk.) Chiov. !	Th	Native	Y	.	.	.	.	.
422	Poaceae	*Setaria verticillata* (L.) P.Beauv.	Th	Alien	.	Y	.	.	.	.
423	Poaceae	*Sporobolus pungens* (Schreb.) Kunth !	G	Native	.	.	Y	.	.	.
424	Poaceae	*Stipellula capensis* (Thunb.) Röser & Hamasha	Ph	Native	.	Y	.	.	.	.
425	Poaceae	*Trisetaria aurea* (Ten.) Pignatti ex Kerguélen	Th	Native	.	Y	.	.	.	.
426	Poaceae	*Triticum aestivum* L.	Th	Alien	.	Y	.	.	.	.
427	Poaceae	*Triticum durum* Desf. !	Th	Alien	.	.	Y	.	.	.
428	Poaceae	*Vulpia ciliata* Dumort. !	Th	Native	.	.	Y	.	.	.
429	Poaceae	*Vulpia fasciculata* Forssk.	Th	Native	.	Y	.	.	.	.
430	Polygonaceae	*Emex spinosa* (L.) Campd. !	Th	Native	.	.	.	.	.	.
431	Polygonaceae	*Polygonum aviculare* L.	Th	Native	.	Y	.	.	.	.
432	Polygonaceae	*Polygonum maritimum* L. !	Ch	Native	.	.	.	.	.	.
433	Polygonaceae	*Rumex bucephalophorus* L. !	Th	Native	.	.	.	.	.	.
434	Polygonaceae	*Rumex conglomeratus* Murray !	H	Native	.	.	.	.	.	.
435	Portulacaceae	*Portulaca oleracea* L.	Th	Native	.	Y	.	.	.	.
436	Potamogetonaceae	*Zannichellia melitensis* Brullo, Giusso & Lanfr. !	Hy	Native [E]	.	.	.	.	.	.
437	Primulaceae	*Lysimachia arvensis* (L.) U.Manns & Anderb. !	Th	Native	.	.	Y	Y	.	.
438	Primulaceae	*Lysimachia loeflingii* F.J.Jiménez & M.Talavera !	Th	Native	Y	.	Y	.	.	.
439	Primulaceae	*Samolus valerandi* L. !	H	Native	.	.	.	.	.	.
440	Pteridaceae	*Adiantum capillus-veneris* L. !	H	Native	.	.	.	.	.	.
441	Ranunculaceae	*Adonis microcarpa* DC.	Th	Native	.	Y	.	.	.	.
442	Ranunculaceae	*Anemone coronaria* L.	G	Native	.	Y	.	.	.	.
443	Ranunculaceae	*Nigella damascena* L. !	Th	Native	.	.	.	.	.	.
444	Ranunculaceae	*Ranunculus bullatus* L. !	H	Native	.	.	.	.	.	.
445	Ranunculaceae	*Ranunculus muricatus* L.	Th	Native	.	Y	.	.	.	.
446	Ranunculaceae	*Ranunculus saniculifolius* Viv.	Hy	Native	.	Y	.	.	.	.
447	Resedaceae	*Reseda alba* L. !	Th	Native	.	.	.	.	.	.
448	Resedaceae	*Reseda lutea* L.	Th	Native	.	Y	.	.	.	.
449	Rhamnaceae	*Rhamnus oleoides* L. !	nPh	Native	.	.	.	.	.	.
450	Rosaceae	*Cydonia oblonga* Mill. !	Ph	Alien	Y	.	.	.	.	.
451	Rosaceae	*Eriobotrya japonica* (Thunb.) Lindl. !	Ph	Alien	Y	.	.	.	.	.
452	Rosaceae	*Malus domestica* (Suckow) Borkh. !	Ph	Alien	Y	.	.	.	.	.
453	Rosaceae	*Prunus domestica* L.	Ph	Alien	.	Y	.	.	.	.
454	Rosaceae	*Prunus dulcis* D.A.Webb !	Ph	Archaeophyte	.	.	.	.	.	.
455	Rosaceae	*Prunus insititia* L. !	Ph	Alien	Y	.	.	.	.	.
456	Rosaceae	*Prunus persica* (L.) Batsch !	Ph	Alien	.	.	.	.	.	.
457	Rosaceae	*Pyrus communis* L. !	Ph	Alien	Y	.	.	.	.	.
458	Rosaceae	*Rubus ulmifolius* Schott	Ph	Native	.	Y	.	.	.	.
459	Rosaceae	*Sanguisorba minor* Bertol. subsp. verrucosa (Ehrenb. ex Decne.) Holmboe !	Th	Native	.	.	.	.	.	.
460	Rubiaceae	*Crucianella maritima* L. !	Ch	Native	.	.	Y	Y	.	Y
461	Rubiaceae	*Cynanchica aristata* (L.f.) P.Caputo & Del Guacchio subsp. scabra (C.Presl) P.Caputo & Del Guacchio!	Ch	Native	.	.	.	.	.	.
462	Rubiaceae	*Galium aparine* L.	Th	Native	.	Y	.	.	.	.
463	Rubiaceae	*Galium murale* M.Bieb. !	Th	Native	.	.	Y	.	.	.
464	Rubiaceae	*Galium verrucosum* Huds.	Th	Native	.	Y	.	.	.	.
465	Rubiaceae	*Plocama calabrica* (L.f.) M.Backlund & Thulin	Ch	Native	.	Y	.	.	.	.
466	Rubiaceae	*Sherardia arvensis* L.	Th	Native	.	Y	.	.	.	.
467	Rubiaceae	*Theligonum cynocrambe* L. !	Th	Native	.	.	Y	.	.	.
468	Rubiaceae	*Valantia hispida* L.	Th	Native	.	Y	.	.	.	.
469	Rubiaceae	*Valantia muralis* L. !	Th	Native	.	.	Y	Y	.	Y
470	Ruppiaceae	*Ruppia maritima* L. !	Hy	Native	Y	.	.	.	.	.
471	Rutaceae	*Citrus limon* Reise Ostindi !	Ph	Archaeophyte	Y	.	.	.	.	.
472	Rutaceae	*Ruta chalepensis* L. !	Ch	Native	.	.	Y	.	.	.
473	Santalaceae	*Thesium humile* Vahl	Th	Native	.	Y	.	.	.	.
474	Scrophulariaceae	*Scrophularia peregrina* L. !	Th	Native	.	.	.	.	.	.
475	Scrophulariaceae	*Verbascum creticum* (L.) Cav.	Th	Native	Y	y	.	.	.	.
476	Scrophulariaceae	*Verbascum sinuatum* L. !	H	Native	.	.	.	.	.	.
477	Simaroubaceae	*Ailanthus altissima* (Mill.) Swingle !	Ph	Inv. Alien	.	.	.	.	.	.
478	Solanaceae	*Hyoscyamus albus* L. !	Th	Native	.	.	.	.	.	.
479	Solanaceae	*Nicotiana glauca* Graham !	Ph	Alien	.	.	.	.	.	.
480	Solanaceae	*Solanum nigrum* L. !	Th	Native	.	.	.	.	.	.
481	Tamaricaceae	*Tamarix africana* Poir. !	Ph	Native	.	.	.	.	.	.
482	Tamaricaceae	*Tamarix gallica* L. !	Ph	Alien	.	.	.	.	.	.
483	Typhaceae	*Typha domingensis* Pers. !	Hy	Native	Y	.	.	.	.	.
484	Urticaceae	*Parietaria judaica* L. !	Ch	Native	.	.	.	.	.	.
485	Urticaceae	*Urtica membranacea* Poir. ex Savigny !	Th	Native	.	.	.	.	.	.
486	Urticaceae	*Urtica pilulifera* L.	Th	Native	.	Y	.	.	.	.
487	Urticaceae	*Urtica urens* L. !	Th	Native	.	.	.	.	.	.
488	Verbenaceae	*Lantana camara* L. !	nPh	Alien	Y	.	.	.	.	.
489	Verbenaceae	*Verbena officinalis* L.	H	Native	.	Y	.	.	.	.
490	Vitaceae	*Vitis vinifera* L. !	Ph	Alien	.	.	.	.	.	.
				Total:	78	163	131	53	20	32

## Results

### Historic records of plants observed on Comino till publications at the end of 2018

Table 3 lists all published records from Comino reported in major publications up to 2018, namely, until Mifsud (2018) and hence before the commencement of our surveys on Comino from 3 February 2019. This extensive literature review yielded a total of 420 verified species.

Taxa that appear in older literature but are excluded from Table 3 are listed in Suppl. material 1: appendix A, which includes the rationale for their omission. Primarily, several taxa used in old work are now obsolete or old synonyms, for example, *Alyssum maritimum* (L.) Lam. [= *Lobularia maritima* (L.) Desv.]; some others were misidentifications of closely related species (e.g. *Juncus hybridus* Brot. was confused and reported as *J. bufonius* L.), while a few others were taxa of species closely related to endemics that were described later from the historic publications [e.g. *Ferula melitensis* Brullo et al. (Brullo et al. 2018) was previously reported as *F. communis* L.].

Some interesting records have emerged from the iNaturalist website (2025; https://www.inaturalist.org). Applying the boundary of Comino and some filters as specified above, 333 observations of 82 plant species were recorded and uploaded to this online platform until September 2024. Two were algae, and one was a marine species [(*Posidonia oceanica* (L.) Delile)]; the rest were terrestrial vascular plants. When browsing these species, none were new records for Comino or plants not recorded by us. The most observed were *Cynara cardunculus* L. (21 observations), *Asteriscus aquaticus* (L.) Less. (16), *Asphodelus ramosus* L. (12), *Capparis orientalis* Veill. (12), and *Euphorbia segetalis* L. subsp. pinea (L.) H.J.Coste (21). The most interesting observations included the rare occurrence of *Ornithogalum arabicum* Brot. (obs. 18 April 2024) and a small plantlet (approximately 20 leaves) of *Nicotiana glauca* Graham (obs. 28 December 2023). The observation of *Micromeria graeca* (L.) Benth. turned out to be a misidentification of *M. microphylla* (d’Urv.) Benth. and was later corrected accordingly in iNaturalist.

Finally, personal communication, verified through photos, includes two new interesting additions to the flora of Comino: *Verbascum creticum* (L.) Cav. (Sciberras 2017), which was observed back in 2005, and *Ophrys bertolonii* Moretti (Cardona 2019), recorded in March 2019. Both species are very rare in Malta.

### Plants observed from surveys conducted between 2019 and 2025

The second phase of this study involved 26 floristic surveys (see Table 2) to record the current species present on the Comino archipelago. The results are presented in Table 4, which, along with the corresponding survey where a particular species was recorded, gives the islands or islets where they were found and an estimated frequency of occurrence for each on the corresponding island or islet. For taxa recorded specifically from Cominotto or from the three smaller islets, please refer to Suppl. material 1: appendix B. Previously recorded taxa that have not been observed during these surveys are listed in Suppl. material 1: appendix F and are referred to as ‘Lost Species’.

This study confirms the observation and photographing of 328 species on the Comino archipelago, of which 306 spp. have been recorded from Comino Island, 131 from Cominotto, 32 from Ħaġra ta’ Taħt il-Mazz, 53 from Ħaġra l-Kbira, and 20 from Ħaġra ż-Żgħira. It is interesting that some 25 species have been found on Cominotto or the islets but are very rare or no longer occur on the larger island of Comino. These have been indicated in a dedicated column also in Table 4. Further statistics of how many species are native, alien, and endemic and their specific growth form according to the Raunkiaer classification are provided in Table 6 and discussed below.

**Table 6.** Overview of species richness within the Comino archipelago, indicating for each island and islet the species present, their status (native, alien, or endemic), and their life-form types according to the Raunkiaer system (Raunkiaer 1934): Therophytes (Th), Chamaephytes (Ch), Hemicryptophytes (H), Phanerophytes (Ph and nPh), Geophytes (G), and Hydrophytes (Hy).

Island/islet	Total plant richness (current and historically recorded)	Current total plant richness	Current native species (inc. arch-aeophytes)	Current alien species	Current endemism (inc. sub-endemism)	Distribution of species life- form types sensu Raunkiaer (1934), for the total records					
						Th	Ch	H	Ph + nPh	G	Hy
Comino archipelago	490	328	282	46	21	261	52	53	64	55	5
Comino island	≃480	306	261	45	19	255	50	52	64	54	5
Cominotto	≃163	131	126	5	12	66	23	17	7	18	0
Ħaġra ta’ Taħt il-Mazz	33	32	32	0	5	18	6	6	2	1	0
Ħaġra l-Kbira	53	53	53	0	5	28	9	5	3	8	0
Ħaġra ż-Żgħira	22	20	20	0	3	9	6	3	3	1	0

### Comprehensive checklist of vascular plants of the Comino archipelago

The published and validated taxa (Table 3) and the new records from our surveys (Table 4) have been combined to create the first comprehensive checklist of terrestrial vascular plants occurring in the Comino archipelago. This checklist is presented in Table 5, and it is sorted by family name, then A–Z of the species per family. Apart from the species name and its author citation and family, the table also provides the status in the Maltese Islands; the Raunkiaer’s life-form classification; which species are New Records (New Rec); which species were previously recorded but were not observed during these surveys, referred to as Lost Records (Lost Rec); and specifies the islands or islets where each species was recorded or observed (marked by !) during the surveys.

### Species richness and life forms of plants from the Comino archipelago

This synthesis allows us to provide statistics on the plant richness for each island or islet of the Comino archipelago. These include the number of native or alien species, the number of endemic taxa, and the distribution of life-forms according to the Raunkiaer classification (Table 6).

This first checklist of the Comino archipelago features a total of 490 valid and confirmed vascular plant species, of which 429 are native, archaeophytes, or reintroduced natives, and 61 are aliens, of which only 12 are declared invasive in Malta. Species referred to as re-introduced are native species that have been recently reintroduced after becoming extinct, and for Comino, these are specifically *Pinus halepensis* L. and *Nerium oleander* L.

Comino Island is undoubtedly the one with the richest flora, as 93% of the plants currently observed (*n* = 328) are found on this island. Despite its much smaller size, Cominotto Island includes c. 40% of the archipelago’s flora, while the islets have a less diverse flora.

From these 490 plant species, 59 are of conservation importance for their endemism, for being listed in the “Red Data Book of the Maltese Islands” (Lanfranco 1989), or for their legal protection according to the Subsidiary Legal Notices SL549.44 (GM 2006) and SL549.123 (GM 2018). For convenience, these are shortlisted in Suppl. material 1: appendix C, which also shows their frequency across the Comino archipelago and identifies new records by displaying species names in bold.

The endemism level is quite elevated, with 21 species (Suppl. material 1: appendix D) out of the 40 or so endemic or subendemic species occurring in the Maltese archipelago. Several of the non-occurring endemics are hydrophytes or chasmophyte species, which are reduced on Comino because the respective cliff or wetland habitats are much more restricted in Comino compared to Malta and Gozo.

A separate list of alien species is provided in Appendix D, indicating those that are declared invasive for Malta. One can appreciate the relatively low level (14%) of the alien flora currently observed (46 species out of the 328 recorded for the archipelago), many of which were casual aliens. Moreover, only five alien species are on Cominotto and none on the smaller islets.

### Species richness and diversity on the Comino archipelago

The dataset generated through this study enables a quantitative assessment of plant diversity across the entire archipelago, as well as for each individual island and islet examined. Specifically, it provides metrics on the proportion of native versus alien taxa, levels of endemism, and the distribution of life-form categories as defined by the Raunkiaer system for each landmass. A summary of these results is presented in Table 6.

Concerning life-forms, therophytes, accounting for about 53% of the plants (total records), are the dominant component occurring in the Comino archipelago, since this life strategy is well adapted to escape the five arid months of summer through seeds and other climatic unpredictability linked to the Mediterranean climate. The other life-forms are less represented (Phanerophytes = 13%; Chamaephytes = 10%; Hemicryptophytes = 11%; Geophytes = 11%), and hydrophytes are very rare (1%) due to the arid nature and geomorphology of the archipelago.

## Discussion

### Rejected and doubtful species recorded in the past

*Ophrys fusca* Link. undoubtedly refers to either *O. caesiella* P.Delforge or *O. iricolor* Desf. s.l., both observed in Comino (Mifsud 2018), and it was excluded from the present checklist. Casha’s (2017) record of *Iris sicula* is of a cultivated specimen that succumbed and was not seen again a few years later (Alex Casha pers. comm., June 2024), and hence this casual introduction is omitted from the flora of Comino. *Vulpia membranacea* (L.) Dumort., cited only by Borg (1927) as a frequent annual grass, is likely confused with some other grass, which is difficult to determine, and is further unsupported by Sommier and Caruana Gatto (1915), who do not include this species in their flora. *Daucus carota* L., reported from the satellite islets of Comino (Sciberras and Sciberras 2010), refers to *D. rupestris* Guss., which is common on the islets, and the authors do not mention it in their checklist. Similarly, *Blackstonia perfoliata* (L.) Huds. was replaced by *B. acuminata* (W.D.J.Koch & Ziz) Domin, as detailed in Suppl. material 1: appendix G; hence, it was also rejected.

The record of *Cynomorium coccineum* from the islet of Ħaġra il-Kbira ta’ Bejn il-Kmiemen (Sciberras and Sciberras 2010) is very odd and unexpected from this islet because records of this parasitic plant from the Maltese Islands have been reported from rupestral habitats (Lanfranco 1960; Stephen Mifsud and Joe Attard pers. obs. 2020–2025). When populations of *C. coccineum* were studied by one of us [SM] in Gozo, the plants were growing on sheltered cliff ledges, about 80 m above sea level in a semi-consolidated sediment of Globigerina (marl-like) limestone and always growing on *Atriplex lanfrancoi* (Brullo & Pavone) G.Kadereit & Sukhor. (originally described as *Cremnophyton lanfrancoi* Brullo & Pavone). The very shallow soil of Ħaġra il-Kbira and the lack of *A. lanfrancoi* or other shrubs serving as a suitable host make the record of *C. coccineum* very anomalous. Moreover, such a conspicuous and persistent species would have been observed by us or others on this very small islet (150 × 70 m), of which only approximately half is well vegetated. In addition, Sciberras and Sciberras (2010) did not provide photographic evidence or any further information on this extraordinary record. As a result, this species is omitted from the present checklist of Comino.

The new description of *Limonium lanfrancoi* Agius, M.E.Galea, Cambria, del Galdo & Brullo from Cominotto (Agius et al. 2023) is treated within the variability of the *Limonium melitense/Limonium zeraphae* complex Brullo, which was found growing in proximity to *L. virgatum* (Willd.) Fourr. and *L. melitense* in Cominotto. A genetic analysis is required to confirm the distinctiveness and taxonomic validity of this species, as the previous assessment relied primarily on morphological characters; an approach that can be problematic and unreliable in the morphologically plastic and nomenclaturally difficult genus *Limonium* (Iamonico and Del Guacchio 2017; Iamonico et al. 2017; Del Guacchio et al. 2018; Koutroumpa et al. 2018; Iamonico et al. 2022).

There are possibly other species recorded in the past on Comino that are doubtful and are likely misidentifications of closely related species. Indeed, this is one of the main explanations why, out of the 420 species reported in historic works, more than 100 species were never confirmed again from Comino. For example, *Phoenix dactylifera* L. is likely referable to *P. canariensis* H.Wildpret, since the two are similar to each other. Some historical records appear to represent reasonably close guesses of species morphologically similar to neoendemics that at that time were not yet described. For example, *Hyoseris lucida* L., first reported from Comino by Borg (1927), clearly refers to the strict endemic *H. frutescens* Brullo & Pavone described in 1988, whereas *Linaria reflexa* (L.) Chaz. (Duthie 1874, 1875a, 1875b) likely refers to *L. pseudolaxiflora* Lojac. described in 1907.

Some determination errors are also expected from historical records, for example: *Ranunculus baudotii* Godr., first reported by Borg (1927), most likely refers to the frequent species *R. saniculifolius* Viv.; *Valantia hispida* L., recorded by Duthie (1875a), may have been confused with the hispid form of *Valantia muralis* L. (sometimes reported in the past as V. muralis var. hirsuta Guss.); *Convolvulus cneorum* L. in Duthie (1875a) is probably *C. oleifolius* Desr.

However, identifying and validating other old records can be less straightforward, as it is unclear whether they are genuine records that became extinct or misidentifications of the past, for example, *Anthemis tomentosa* L., *Biscutella lyrata* L., *Catapodium hemipoa* (Delile ex Spreng.) Laínz, *Stellaria media* (L.) Vill., (refer to Appendix F). Each doubtful record requires a thorough study in itself, and, for the benefit of the doubt and given the lack of tangible proof for these suspected mistakes, we kept many such entries on the checklist until stronger evidence or studies could support their exclusion.

### Comparison between historical records and current observations

Comparing ancient records from the 1830s to the 1930s with current observations reveals both a remarkable number of new records as well as many undetected/unobserved species. A total of 78 species are new records for the Comino archipelago, comprising 47 native or archaeophytes and 32 alien species. These new taxa are provided in Suppl. material 1: appendix E. Many of these species have a rare occurrence and hence were simply undetected by previous explorers, yet it is rather unbelievable that common species like *Pistacia lentiscus* were only recorded recently (Sciberras and Sciberras 2010).

Conversely, a considerable proportion of historically reported species were not detected during the 26 surveys undertaken. Owing to uncertainties regarding the reliability of some historical records, as well as the possibility that certain taxa may persist undetected, it is premature to classify all of these species as extirpated from the Comino archipelago. Accordingly, they are treated as lost records and listed in Appendix F, which currently comprises approximately 160 taxa. In contrast, species associated with highly threatened habitats, particularly sand dune specialists, hydrophytes, and agriculturally related wild plants (including former agricultural escapes), can be regarded as extirpated with a high degree of confidence. For instance, the San Niklaw and Santa Marija dune systems on Comino Island have undergone severe disturbance for a long period of time, local water sources have declined markedly over the last decades, and traditional farming ceased by the 1970s, all leading to severe habitat degradation and the loss of mandatory conditions to sustain the ecological niche of these taxa.

Species from other habitats may have become extirpated from Comino Island, and their disappearance can be attributed to direct human activities such as tourism, trenching, hunting, fires, and land reclamation or to indirect pressures such as climate change or the introduction of pests and diseases. Indeed, the second part of Appendix E lists species that have been lost in the last 30 years, where, for example, a single specimen of *Verbascum creticum* observed by Jeffrey Sciberras in 2005 (Jeffrey Sciberras pers. comm., Oct 2012) may have succumbed due to heat stress and drought that took place in the 2010s.

### Species turnover in relation to habitat changes

#### - Decreased occurrence of Cichorioideae Chev. (Asteraceae)

The floristic composition of Comino Island reveals a markedly diminished diversity and abundance of species within the subfamily Cichorioideae when compared to the neighbouring islands of Malta and Gozo. Taxa commonly encountered in Malta, such as those belonging to the genera *Carthamus*, *Carlina*, *Hyoseris*, *Leontodon*, *Reichardia*, *Scolymus*, *Sonchus*, and *Urospermum*, among others, are very rare or extirpated in Comino. A defining morphological characteristic of many Cichorioideae species is the presence of lactiferous tissues that exude milky latex. The conspicuous absence of these taxa on Comino is plausibly attributed to sustained herbivory pressure exerted by feral rabbit populations [*Oryctolagus cuniculus* (Linnaeus, 1758)], which were reintroduced in great numbers to the island during the 17^th^ century by the Knights of Malta for recreational hunting. Despite the passage of centuries, wild rabbits remain prevalent on Comino and are frequently observed during field surveys. Given the palatability of latex-producing species to rabbits, it is hypothesised that selective browsing has significantly suppressed Cichorioideae populations. This trophic interaction may also underlie the broader scarcity of other herbaceous taxa on the island, suggesting a long-term ecological shift driven by these introduced herbivores. This observation is corroborated by the fact that the impact of rabbits on island ecosystems is well documented on some Mediterranean islands (e.g., Kossoff et al. 2024).

#### - Wetland species

Historic records mention several wetland species that are no longer extant on Comino Island. These include *Carex divisa* Huds., *Juncus acutus* L., *Juncus maritimus* Lam., *Juncus bufonius* L. s.l. (possibly for *J. hybridus* Brot.), *Melilotus siculus* (Turra) Steud. (= *M. messanensis* (L.) All.) and *Phragmites australis* L. Their persistent reporting in the past (Duthie 1874, 1875a; Sommier and Caruana Gatto 1915; Borg 1927) suggests that they occurred in the marshland area behind Santa Marija Bay, which was destroyed in 1991 (see Introduction) and converted into a recreational area. Further evidence was given by Camilleri et al. (2012), who mentioned that the last stands of *Phragmites australis* were transplanted from the Santa Marija marshland to an artificial pond in 1990. In 2020, attempts were made to restore the marshland; however, as of the time of writing, the area is still overrun with pioneer and ruderal species (e.g., Dittrichia viscosa subsp. viscosa), and efforts to introduce wetland species have not been successful. On the other hand, *Ruppia maritima* L. s.l. was first recorded from Comino in an artificial water pool situated at the Ta’ Ħażina area in May 2022 (located close to TPA001KM, https://era.org.mt/wp-content/uploads/2019/05/Il-Hażina_Kemmuna.pdf) and was later successfully transferred and naturalised at the Santa Marija marshland. Another new record is the red-listed *Typha domingensis* Pers. in a small water reservoir. *Zannichellia melitensis* Brullo, Giusso & Lanfr. (sensu auct. melit.) was observed in two small populations and is very threatened on Comino Island.

#### - Cultivated crops and agriculture

Some crops and vegetables that were cultivated on Comino in the early 20^th^ century have been documented by Borg (1927) and nowadays are no longer seen due to the abandonment of agriculture, which started to decline in the 1970s (Farrugia Randon and Farrugia Randon 1995). Examples include *Allium cepa* L. (onions), *A. sativum* L. (garlic), *Cuminum cyminum* L. (cumin), and *Eruca vesicaria* (L.) Cav. (rocket, rucola); *Ervilia sativa* Link (ervil, a type of pulse crop, which became more used as fodder due to its bitter taste); *Hordeum vulgare* L. (barley); *Prunus domestica* L. (plum tree); *Prunus persica* (L.) Batsch (peach tree); and *Triticum* spp. (wheat). In addition, fig and vine trees (*Ficus carica* L. and *Vitis vinifera* L.) were introduced in large numbers in the early 20^th^ C. and later, in the 1970s and 1980s, whereas Aleppo pine, oleander, *Acacia* and some *Eucalyptus* trees have been introduced for embellishment (Farrugia Randon and Farrugia Randon 1995; Camilleri et al. 2012). Since the early 2020s, various tree species have been reintroduced, but only a few, such as tamarisk and Aleppo pine, have been established successfully. As a result, subsequent afforestation efforts have relied heavily on *Pinus halepensis* Mill., often in excessive numbers and in inadequate places, competing with native vegetation.

#### - Sand dune species

Although there are three sandy bays in Comino, namely Blue Lagoon (including Cominotto), St Nicholas Bay and Santa Marija Bay, only the latter harbours a small but established sand dune community. The plants presently recorded from the Santa Marija sand dune (excluding non-arenous species and ruderals) are only limited to *Cakile maritima* Scop., *Echium arenarium* Guss., *Glaucium flavum* Crantz, *Medicago marina* L., *Pancratium maritimum* L. and *Polygonum maritimum* L. (very threatened). In addition, *Lotus halophilus* Boiss. & Spruner was rediscovered and reported by Lanfranco and Stevens (2000) and reconfirmed by one of us [SM] on 16 Apr 2007 but has not been encountered again in our surveys. On 16 June 2012, [SM] also recorded the rare and strictly protected *Euphorbia peplis* L., but it was not seen again in subsequent years due to the degradation of the sand dune by multiple pressures (Fig. 6E, F), and it is now believed to be extinct on Comino.

The degradation of this sand dune has been alerted for some three decades by various ecologists, but nothing effective has seemed to be done except cordoning off the area with ropes. On the contrary, a part of the sand dune was scraped to accommodate more sunbeds for a tourist operator in summer 2016 (Stephen Mifsud pers. obs., Jun 2016). Fortunately, this operated only for three years (Tripadvisor 2019). The small beach becomes overcrowded when more than 200 tourists arrive at once from large vessels docking at the jetty near Santa Marija Beach. When this happens, some visitors have been seen stepping over the cordon to sunbathe on the sand dune, trampling the protected area (Fig. 6E). Enforcement officers and rangers are rarely present to educate and enforce environmental regulations in this Natura 2000 site’s sand dunes.

It is then no surprise that this long-term degradation has brought about the extirpation of several sand-dune species recorded by Duthie (1874, 1875a, 1875b), Sommier and Caruana Gatto (1915), Borg (1927), Haslam et al. (1977) and others, namely, *Ambrosia maritima* L., *Euphorbia peplis*, *Euphorbia paralias* L., *Euphorbia terracina* Lag., *Lotus halophilus* Boiss. & Spruner, *Neatostema apulum* (L.) I.M.Johnst., *Daucus pumilus* Ball (= *Pseudorlaya pumila* (L.) Grande), *Scolymus hispanicus* L. and *Sporobolus pungens* (Schreb.) Kunth (a surviving population is only present on Cominotto). With more than half of plant biodiversity lost from the Santa Marija sand dune, conservation measures and enforcement are desperately needed, in concurrence with previous recommendations advocated by ecologists over the last decades (e.g., Haslam et al. 1977; Lanfranco 2004; Camilleri et al. 2012).

#### - Tree assemblages and woodlands

Due to the arid and rocky nature of the island, as well as its shallow soils, very few autochthonous woodlands or copse of trees are found on Comino. The most significant are:

1. A very ancient copse of olive trees situated in an area known as il-Ħażina, whose antiquity resulted in their designation as a tree-protected area (TPA001KM, https://era.org.mt/wp-content/uploads/2019/05/Il-Hażina_Kemmuna.pdf).
2. Maquis trees (carob, olive and almond trees) at the valley banks of Wied l-Aħmar.
3. A copse of *Vitex agnus-castus* and *Tamarix africana* trees at Santa Marija Bay, with one example of tamarisk being of an extraordinarily large size and antiquity.

In addition, the introduction of trees (mostly alien), mostly during the last 150 years, is significant, and although they have not been aggressively invasive in Comino, some tree species formed sizeable populations. These include:

1. *Acacia* spp. and *Eucalyptus* spp., typically introduced in Malta in the 1970s and 1980s for bird-hunting purposes.
2. *Ailanthus altissima*, an escape from ornamental and casual introduction in the garden located beside the chapel.
3. *Cupressus sempervirens*, introduced in the cemetery that served as a burial site for individuals quarantined on the island and became naturalised to a sizeable population outside the cemetery.
4. *Nerium oleander*, introduced to embellish some pathways leading to the Blue Lagoon, the Palazz and Isolation Hospital, the hotel, and Santa Marija Beach.
5. Agricultural fruiting trees were planted and farmed by farmers mostly in the early and mid-20 ^th^ C., many of which are still alive and without any irrigation or attention. These include citrus, fig, olive, almond, stone fruit and grapevine trees. Some accounts provide further detail, stating, for example, that 3,750 vines, 400 figs, and 100 carob trees were planted in 1912 (Gauci 1993; Falzon 2004). In the late 1970s and early 1980s, another afforestation of olive and fig trees was conducted together with Aleppo pine, tamarisk and *Acacia* trees (Borg 2002; Falzon 2004).
6. *Pinus halepensis*, representing the most abundant tree, yet it is alien to Comino (Camilleri et al. 2012; Edwin Lanfranco pers. comm., Apr 2021). They have become established in naturalised, large, dense copses that are expanding slowly. The introduction and naturalisation of *Pinus halepensis* on Comino requires further discussion.

#### - *Pinus halepensis* on Comino

The mature trees on Comino are not native, as all native trees were eradicated from Malta a long time ago, possibly as early as the Bronze Age and again during the Arab period. Consequently, reintroductions of this tree in Malta have originated from foreign stock. Claims that seeds from some surviving trees were used to reintroduce the species in Malta (ERA n.d.) are so far unfounded and unlikely, especially for Comino, given that plantations took place in the 1970s and 1980s (Falzon 2004), and there was no awareness of conserving local genotypes at that time.

Despite being one of the most resilient and successful trees in afforestation projects in the Mediterranean region (Alsanousi et al. 2025), its naturalisation, expansion, and habitat displacement in rocky places have been discussed (Maestre and Cortina 2004; Osem et al. 2011; García-de-Lomas et al. 2023) and observed repeatedly in mainland Malta (Baldacchino 1984; Edwin Lanfranco pers. comm., Apr 2021; SM pers. obs. 2015–2025). Typically, trees grow slowly but readily radially away from the introduced areas, later displacing the native and endemic flora due to overshadowing, root mass, competition for water, and the formation of thick carpets of slowly decomposing leaf litter these trees produce. Its spread into rocky habitats often leads to reduced biodiversity and the homogenisation of plant communities below and near them.

Ambjent Malta, a governmental entity (https://ambjentmalta.gov.mt/) that undertook a habitat restoration initiative on Comino, involving the planting of over 13,000 native trees and shrubs as part of a broader conservation strategy within the Natura 2000 network (Times of Malta 2021), has mostly introduced *Pinus halepensis* on Comino. This is possibly because the Aleppo pine demonstrated itself to be the most successful of the different trials of tree species. These plantations were initially intended for abandoned, barren fields, making them a strategic choice for afforesting areas with low biodiversity. However, later afforestation was implemented in pristine garigue habitats and pathways adjacent to the garigue and phrygana with protected species such as *Euphorbia melitensis* Parl. and Anthyllis hermanniae subsp. melitensis Brullo & Giusso, *Thymbra capitata* (L.) Cav., and local orchids (Fig. 6G). The introduction of canopy-forming species such as *P. halepensis* into these open, sun-exposed systems poses significant ecological pressures and risks, as mentioned above.

The introduction of pine and other conifer trees in Comino by central authorities in excessive amounts, including in sensitive areas with endemic or protected plants, must be urgently revised by the Environment and Resource Authority of Malta, as already suggested by Masini (2022): “*It was evident from the Comino case study that afforestation on the islet needed to deviate from a quantity-oriented approach and focus more on appropriate post-planting maintenance, whilst investing in re-introduction of threatened local species*.”.

### Ecologically sensitive plant species

#### - Endemic species

Despite its adverse climatic and geographic conditions and its small size, the Comino archipelago offers refuge to a number of ecologically sensitive species, namely endemic, protected, red-listed or/and very rare. These are provided in the dedicated tables in Suppl. material 1: appendices C, D. In this archipelago, 21 endemic or subendemic species are currently recorded (Table 7). As expected, Comino offers “pristine” habitats supporting large populations of *Anthyllis hermanniae* L. subsp. melitensis Brullo & Giusso (Fig. 2C) and *Euphorbia melitensis* Parl. (Fig. 2D), which are considered the most frequent endemics on the archipelago.

**Table 7.** List of endemic (E) and subendemic (S) species and their occurrences (CC: very common; C: common; S: scarce; R: rare; RR: very rare) on Comino Island, Cominotto, and the three larger islets, and the date of the respective survey (1: 3 Feb 2019; 2: 30 Mar 2019; 6a, 6b: 4 Jun 2020; 10: 26 Mar 2021; 14: 4 May 2021; 20: 26 Nov 2024; 21: 4 Apr 2025).

Species	Status	Frequency on the Comino Island	Frequency on Cominotto Island	Frequency at Ħaġra taħt il-Mazz	Frequency at Ħaġra l-Kbira	Frequency at Ħaġra ż-Żgħira	Visit when first observed on the respective island or islet
*Allium lojaconoi*	S	R	RR	-	I	-	1 6a × 14 ×
Anacamptis pyramidalis subsp. urvilleana	E	RR	-	-	-	-	Mar 2007
Anthyllis hermanniae subsp. melitensis	E	CC	CC	RR	-	-	1 6a 6b × ×
*Chiliadenus bocconei*	E	R	-	-	-	-	2 × × × ×
*Daucus lopadusanus*	S	R	-	-	-	-	2 × × × ×
*Daucus rupestris*	S	S	R	CC	C	S	1 6a 6b 14 6b
*Desmazeria pignatti*	S	S	S	R	-	RR	1 10 6b × 6b
*Euphorbia melitensis*	E	CC	S	-	-	-	1 6a × × ×
*Ferula melitensis*	E	C	S	-	-	-	1 6a × × ×
*Hyoseris frutescens*	E	X	-	-	-	-	Not observed
Jacobaea maritima subsp. sicula	S	S	S	-	RR	-	1 6a × 14 ×
*Limonium melitense*	E	S	R	R	R	S	1 6a 6b 14 6b
*Limonium zeraphae*	E	R	R	-	-	-	1 6a × × ×
*Linaria pseudolaxiflora*	S	R	RR	-	-	-	1 10 × × ×
Matthiola incana subsp. melitensis	E	-	-	S	-	-	× × 6b × ×
*Ophrys melitensis*	E	-	RR	-	-	-	× 21 × × ×
*Romulea variicolor*	S	R	RR	-	S	-	1 21 × 14 ×
*Salsola melitensis*	E	S	R	S	-	R	1 6a 6b × 6b
*Scilla sicula*	S	X	-	-	-	-	Not observed
*Senecio pygmaeus*	S	R	R	-	-	-	1 20c × × ×
*Zannichellia melitensis*	E	RR	-	-	-	-	1 × × × ×

Another endemic group is that of sea lavender, with *L. zeraphae* Brullo and *L. melitense* Brullo commonly found with *L. virgatum* (Willd.) Fourr. on the shallow coasts of Comino and Cominotto. *L. lanfrancoi*, recently identified from Cominotto (Agius et al. 2023), is here considered with taxonomic doubt, and its distinction with *L. melitense* appears not so clear; molecular analyses are needed, in our opinion, to confirm its status.

**Figure 4.**
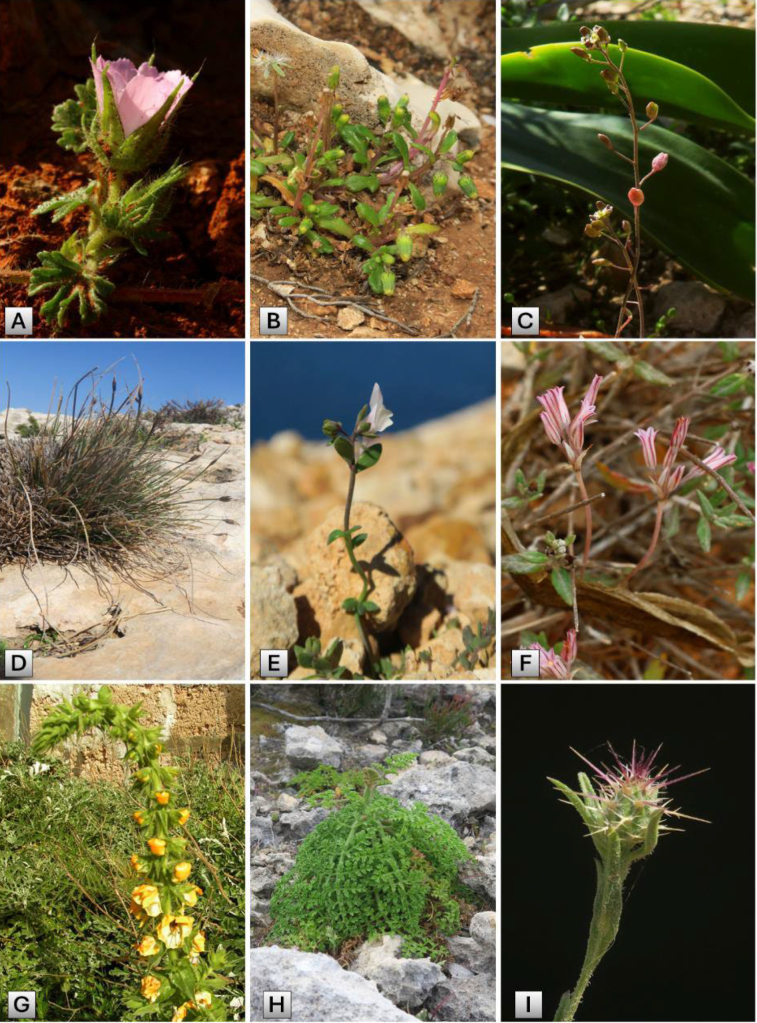
Selection of strictly protected, rare or/and ecologically sensitive species from the Comino archipelago. **A**. *Malva setigera* (= *Althea hirsuta*) (11 Apr 2021); **B**. *Senecio pygmaeus* (4 Apr 2025); **C**. Hornungia procumbens subsp. revelierei (4 Apr 2025); **D**. *Schoenus nigricans* (26 Mar 2021); **E**. *Linaria pseudolaxiflora* (31 Mar 2019); **F**. *Allium lojaconoi* (27 May 2022); **G**. *Verbascum creticum*, discovered in Comino and photo supplied by Jeffrey Sciberras (9 Apr 2004); **H**. Daucus communis subsp. tenuifolia (15 Apr 2021); **I**. *Centaurea melitensis* (5 May 2019).

Other important endemics are Matthiola incana subsp. melitensis (Sciberras et al. 2009, 2010), as it extends its natural range, which mostly falls in the western part of Gozo; *Senecio pygmaeus* (Fig. 4B); *Linaria pseudolaxiflora* Lojac. (Fig. 4E); and *Allium lojaconoi* Brullo, Lanfr. & Pavone (Fig. 4F), found in several pockets on Comino and Cominotto and also considered threatened and rare endemic plants for the Maltese Islands.

*Hornungia procumbens* (L.) Hayek subsp. revelierei (Jord.) Giardina & Raimondo from Comino was also considered endemic by Brullo’s school as *Hymenolobus revelierei* (Jordan) Brullo subsp. sommieri (Pamp.) Brullo, apparently for its small size. However, upon observing large plants in more fertile soil (Fig. 4C), its claimed endemism is highly questionable, and until further verification is substantiated, it is treated here in synonymy with H. procumbens subsp. revelierei, as in global classification (GBIF 2026; iNaturalist 2026; POWO 2026; WFO 2026).

#### - Strictly protected species

**Figure 5.**
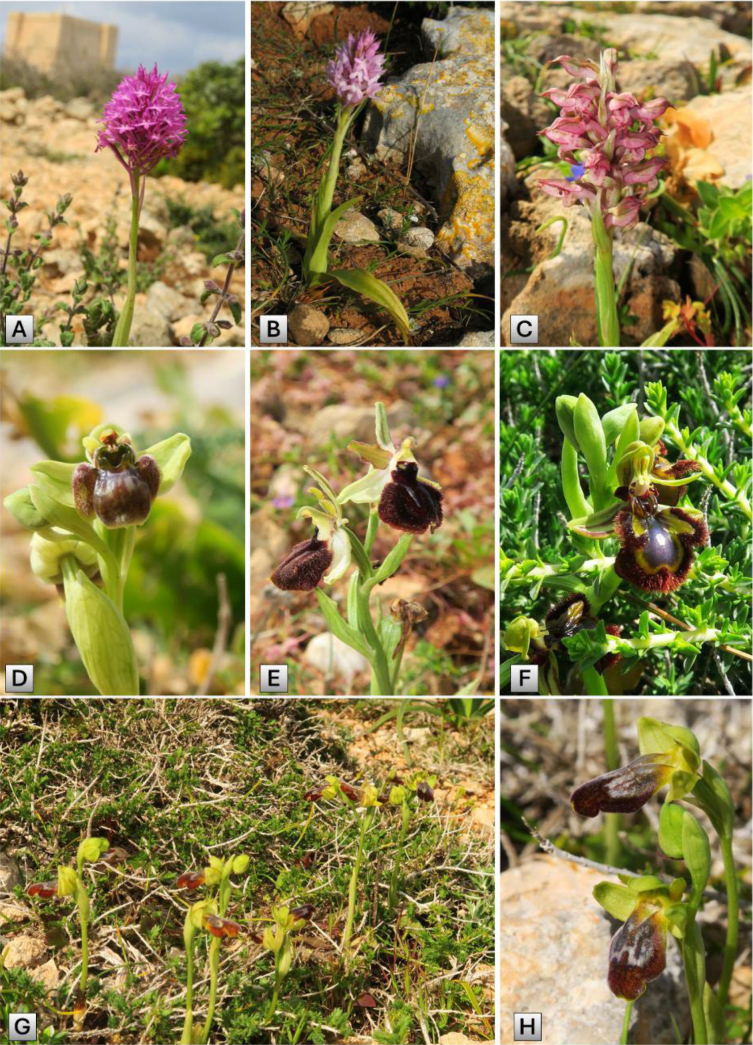
Orchids recorded from the Comino archipelago. **A**. *Anacamptis pyramidalis* s.str. (31 May 2025); **B**. Anacamptis pyramidalis subsp. urvilleana (23 Mar 2007); **C**. Anacamptis coriophora subsp. fragrans (4 Apr 2025); **D**. *Ophrys bombyliflora* (2 Mar 2023); **E**. *Ophrys melitensis* (4 Apr 2025); **F**. *Ophrys speculum* (4 Apr 2025); **G**. *Ophrys caesiella* (= *O. subfusca*), including; **H**. *Ophrys × tumentia* (15 Mar 2022).

A total of 58 legally protected species of vascular plants is recorded on Comino (Appendix C), many of which are also included in the Red Data Book by Lanfranco (1989). Several species have been observed and confirmed in this study, except for the following species: Anacamptis pyramidalis subsp. urvilleana (Sommier and Caruana) Landwehr – last observed in 2007 by one of us (Fig. 5B); *Neotinea lactea*, *Scilla sicula* and *Prunus domestica* are not rare in Malta, so they may simply have not been spotted in our surveys; *Valantia hispida* and *Phoenix dactylifera* are assumed to be past misidentifications for V. muralis var. hirsuta and *P. canariensis*, respectively; *Euphorbia paralias*, *Euphorbia peplis* and *Euphorbia terracina* are assumed to be extinct sand dune species from the small Santa Marija Bay. *E. peplis* was observed by one of us (Stephen Mifsud pers. obs., 16 June 2012) and vanished around the year 2020. Other examples of protected species that are probably extirpated from Comino include *Hyoseris frutescens*, *Juncus maritimus*, *Ophrys bertolonii* Moretti and *Plocama calabrica* (L.f.) M.Backlund & Thulin. Specific remarks for these lost taxa are found in Suppl. material 1: appendix F.

#### - Rare species of ecological significance and new records for the Maltese Islands

Apart from the endemic and protected species, Comino and Cominotto islands harbour other rare or ecologically important species, many of which are listed in the Red Data Book of the Maltese Islands (Lanfranco 1989). Picking some of the most remarkable examples, one can mention the following: *Malva setigera* K.F.Schimp. & Spenn. (Fig. 4A) (always recorded from Comino as *Althea hirsuta* L.), possibly only present on Comino within the Maltese Islands; *Schoenus nigricans* L. (Fig. 4D), found in Cominotto and known in only two other places in Malta (Armier Peninsula and the limits of Wied Rini – pers. obs. Stephen Mifsud); *Verbascum creticum* (Fig. 4G), possibly extinct from Comino, as it was last seen in the mid-2010s (Jeffrey Sciberras pers. comm., Oct 2012); *Hornungia procumbens* (Fig. 4C), very rare in Malta, and the most established population occurs on Comino and Cominotto (new record); *Centaurea melitensis* L. (Fig. 4I), very rare, and possibly Comino is one of the last stations in Malta; Daucus carota subsp. commutatus var. tenuisectus (Fig. 4H), *Orobanche balsensis* (J.A.Guim.) Carlón, M.Laínz, Moreno Mor. & Ó.Sánchez (recorded from Comino as *O. minor* Sm.) and *Catapodium pauciflorum* (Merino) Brullo, Giusso, Miniss. & Spamp., corresponding to new records for the Maltese Islands (unpublished material); *Lotus halophilus*, a very threatened sand dune species (Lanfranco and Stevens 2000), possibly lost from Comino now; and *Ruppia maritima* (new record), a very threatened brackish species.

Apart from the selected topics discussed above regarding the ecology and biodiversity of plants on Comino, many other species also warrant some commentary or interesting observation. Further brief remarks on several species are catalogued in Suppl. material 1: appendix G.

#### - Orchids

Compared to Malta and Gozo, the diversity and abundance of orchids on the Comino archipelago are relatively low and extremely scarce. Only 12 species are recorded here (Table 5), of which only ten were observed in these surveys. Some 40 orchid species are recorded and extant in Malta (Mifsud 2018). Even the common orchids, like *Anacamptis collina* (Banks & Sol. ex Russell) R.M.Bateman, Pridgeon & M.W.Chase, *Neotinea lactea* (Poir.) R.M.Bateman, Pridgeon & M.W.Chase, *Ophrys bombyliflora* Link, Anacamptis coriophora subsp. fragrans (Pollini) K.Richt. and *Serapias parviflora* Parl., are only to be found in few numbers or are absent on Comino Island. The most encountered orchid on Comino is *Anacamptis pyramidalis* (L.) Rich. s.str. (Fig. 5A), scattered here and there but found as individuals. *Ophrys caesiella* P.Delforge (*O. subfusca* (Rchb.f.) Hausskn. sensu Lowe) (Fig. 5G), including the hybridogenous *O. × tumentia* Mifsud (Fig. 5H), is rare but found in dense populations, one of which comprised about 80 to 100 individuals. One of the most interesting records from Comino was that of Matthew Borg Cardona, who in April 2019 spotted and photographed a single specimen of the very rare *Ophrys bertolonii* beside a rocky pathway (Matthew Borg Cardona pers. comm., Mar 2019). This individual was never seen again. Another notable record was an orchid with relatively large leaves found in one of the surveys on Cominotto (26 Nov 2025), which resembles *Himantoglossum robertianum* (Loisel.) P.Delforge, but since it did not flower the following winter and its identity cannot be confirmed, it was not included in the present checklist. The endemic A. pyramidalis subsp. urvilleana and *Ophrys melitensis* (Salk.) Devillers-Tersch. & Devillers, recorded by Stevens (2000) and Bartolo et al. (2001), respectively, have been reconfirmed either in these surveys (*O. melitensis*, Fig. 5E) or observed by Mifsud in 2007 (A. pyramidalis subsp. urvilleana, Fig. 5B). The common Maltese orchids A. coriophora subsp. fragrans (Fig. 5C), *O. bombyliflora* (Fig. 5D) and *Serapias parviflora* Parl. are very rare on Comino Island, whereas only one individual of *O. speculum* Bertol. (Fig. 5F) was observed, which otherwise occurs frequently in Gozo.

The limited occurrence of orchid species on the island of Comino remains an unresolved ecological question. Several factors may contribute to this phenomenon, including the absence of essential mycorrhizal fungi in the soil, which are critical for orchid germination and growth, as well as the island’s pronounced aridity and low water retention capacity due to the negligible presence of clay. Consumption of bulbs by herbivores (e.g. rats) or their foraging by previous mediaeval or Arabic colonisers for obtaining salep is a remote but possible speculation, given that Comino was inhabited by pirates for centuries (Boffa 1966; Farrugia Randon and Farrugia Randon 1995).

### Alien flora

A total of 61 alien species were recorded on the Comino archipelago, many of which consist of one or just a few individuals forming very small and restricted populations, usually escapees from abandoned cultivation. However, twelve of these are declared as invasive in Malta, of which only six species were observed to threaten (or potentially do) the native flora of the Comino archipelago (see below). Yet, except for *Oxalis pes-caprae* L., which spread extensively on the island of Comino (and, to a lesser extent, on Cominotto), the other invasive species observed are rather contained in sizeable pockets.

#### - Occurrence and methods of introduction

Cominotto and the smaller islets have no or negligible alien flora, whereas Comino Island has at least three main artificial areas from which alien species were introduced and spread into natural habitats, mainly consisting of cultivated exotic and ornamental plants. These are:

1. The main spillage of aliens into natural habitats are the gardens, embellished terraces, modified land, and fields belonging to the Comino hotel, close to San Niklaw Bay and the hotel’s bungalows on the western shore of Santa Marija Bay (refer to map in Fig. 1). The introduction of ornamental plants went on for these 50 years that the hotel was in operation, between the late 1960s and 2019 when it closed. Cultivated horticultural species from these artificial sites are not included in the checklist (e.g. *Centaurea ragusina* L. and *Gerbera* sp.), but escapees in adjacent natural areas were recorded (e.g. *Carpobrotus acinaciformis* (L.) L.Bolus, *Aptenia* spp.).
2. Gardens and fields of a residence currently with two inhabitants living on Comino but which were more numerous in the late 1900s. They live on the premises of il-Palazz (refer to the ‘old hospital’ in Fig. 1), and apart from the main garden, there are scattered cultivation plots and some fields which are now mostly in a rather abandoned state. Only a few table crops (potatoes, tomatoes, aromatic herbs, etc.) are still managed in the main garden by these old residents. Most alien species here were perennials (trees, succulents, and ornamentals) that persisted after being completely abandoned.
3. Another source of alien species is the dilapidated pig farm in the southeastern part of the island. These premises have an open, embellished area where some ornamental species have managed to escape at or close to the ruins, but many have not spread widely away from this site.

Mature fruiting trees in previously cultivated fields at il-Ħażina and the lower reaches of Wied l-Aħmar are also included within the alien flora of Comino and are considered relics of abandoned cultivation but offer no threat to the native flora.

#### - Invasive or potentially invasive species for Comino Island

**Figure 6.**
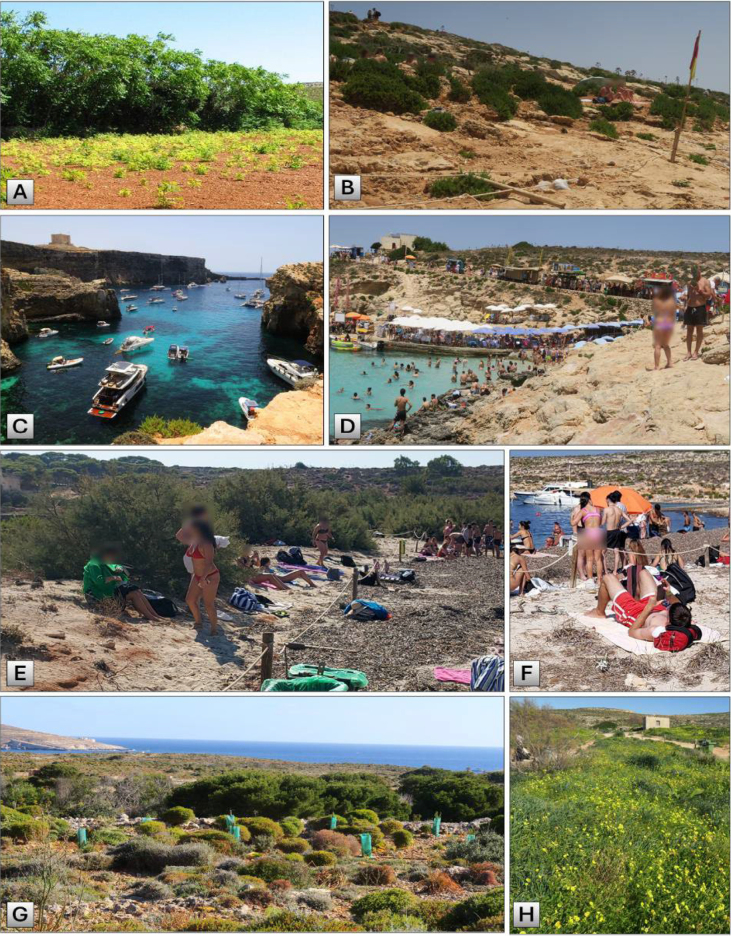
Main ecological pressures and threats for Comino. **A**. Invasive alien species, in particular *Ailanthus altissima*. **B–E**. Overtourism; **B**. Excessive moored boats and ships, including larger ones at Blue Lagoon; **C**. Trampling of tourists since the beaches are too small to accommodate everyone; **D**. Excessive tourists at the Blue Lagoon area out of the peak season (5 Jun 2022); **E**. Tourists jumping cordons and trampling the strictly protected Santa Marija sand dune due to insufficient space on the small beach and no enforcement officers to manage the situation; **F**. Sunbathing and smoking on the protected sand dune; **G**. Unnecessary afforestation in pristine natural habitats with the genetically alien *Pinus halepensis*, here planted among the endemic *Euphorbia melitensis* and Anthyllis hermanniae subsp. melitensis, intermixed with *Thymbra capitata* and other local garigue-type labiates; **H**. Degraded state of the Santa Marija sand dune, dominated by *Oxalis pes-caprae* and other ruderal species.

Compared to Malta and Gozo, the island of Comino appears to be less affected by invasive plant species, likely due to its relatively lower levels of anthropogenic disturbance. Field surveys have identified five species currently exhibiting invasive behaviour within natural habitats, along with one additional species that may pose a future threat. These species are listed below in descending order of ecological concern:

1. *Ailanthus altissima* (Mill.) Swingle (Fig. 6A) – Originally introduced as an ornamental plant in the garden behind the chapel (Camilleri et al. 2012), this species has proliferated extensively across fields and the semi-wetland zone at the mouth of Wied l-Aħmar valley, demonstrating aggressive colonisation.
2. *Oxalis pes-caprae* (Fig. 6H), forming intensive carpets in abandoned and a few disturbed places but does not particularly invade rocky areas, possibly due to shallow soil and aridity.
3. *Agave* spp. There have been only a few localised populations of *Agave*, which, however, exhibited gradual expansion in recent years, raising ecological concerns due to their invasive potential. Thankfully, they were removed by the Environment and Resources Authority in 2019–2020, and while these interventions were largely effective, residual pockets of *Agave* remain, and sporadic regrowth has been observed.
4. *Carpobrotus* spp., introduced as ornamental species in the Comino Hotel and escaped in a few places close to the shores near the hotel and had formed extensive but localised carpets. Many populations have been eradicated by competent authorities too.
5. *Acacia saligna* (Labill.) H.L.Wendl., naturalised successfully after its introduction about 50 years ago but has not spread that much from its source of introduction. Comino’s shallow, arid soil, lacking the clay component, may have slowed its spread compared to Malta and Gozo, where this tree exhibits vigorous growth in clayey soil. Competent authorities chopped down several trees and populations of *Acacia* on Comino.
6. *Pinus halepensis*, introduced in the second half of the 20 ^th^ C. and considered alien to Comino (see discussion above), forms sizeable copses that drastically degrade the biodiversity beneath them.

### Frequently occurring and conspicuous species not recorded in historic accounts

Although pioneer botanists have been very careful to record as many plants as possible – but mainly from Comino Island, including tiny species such as *Centaurium pulchellum* (Sw.) Hayek ex Hand.-Mazz., Stadlm., Janch. & Faltis, *Malva setigera*, and *Hornungia procumbens* – it is surprising that none have recorded, for example, the mature and ancient *Olea europaea* L. stand in the central area of Comino (known as tal-Ħażina) or the shrub forms (var. sylvestris) scattered across Comino. Camilleri et al. (2012) only mention recently introduced olive trees for afforestation. The same can be said for *Cupressus sempervirens* L., which is abundant at the cemetery and the chapel; *Erica multiflora* Biv.; and *Lonicera implexa* Aiton, forming conspicuous populations here and there, whereas *Salsola melitensis* is abundant on the cliffs of Comino and all the islets. The record of *Suaeda vera* Forssk. ex J.F.Gmel. (Sommier and Caruana Gatto 1915) may have been a misidentification of this endemic plant since the former has never been observed. Aleppo pine, oleander, *Eucalyptus*, and *Acacia* trees were not recorded by Haslam et al. (1977) or in previous literature; therefore, they must have been introduced after the late 1970s. *Ceratonia siliqua*, exhibited as huge trees at Wied l-Aħmar, was only reported by Sommier and Caruana Gatto (1915). Yet the most astounding “omission” is that of *Pistacia lentiscus* L., which in our surveys is among the most abundant species present everywhere, yet its first mention is by Sciberras and Sciberras (2010) from Cominotto and Taħt il-Mazz islet.

### Anthropogenic disturbances

The conservation of Comino’s biodiversity can be largely attributed to its historically low levels of human habitation and minimal anthropogenic disturbance. Unlike many other Mediterranean islands, where the expansion of agricultural land was a widespread response to population growth and subsistence needs, Comino experienced only limited transformation of its natural habitats into cultivated fields. This relative absence of intensive land-use change has allowed much of the island’s native vegetation and ecological integrity to persist. At its demographic peak, Comino supported a small community of approximately 120 residents for a relatively brief period of less than a century. Such a population density was ecologically sustainable given the island’s size and resource base. Built infrastructure remained sparse, with only a handful of permanent structures ever established. The most notable among these were an isolation hospital, a traditional bakery, a single hotel, a pig farm, and a defence battery and tower – developments that, while significant, did not result in widespread habitat fragmentation or irreversible ecological degradation.

The period of limited human presence on Comino began to decline once the island emerged as a tourist destination in the late 1960s. Initially, visitation levels remained within sustainable limits, a situation that persisted until the early 2010s. However, by that time, the concentration of visitors (predominantly tourists) at focal sites such as the Blue Lagoon and Santa Marija Bay had reached unsustainable levels, fluctuating between approximately 5,000 and 12,000 individuals per day during peak periods (approximately 800,000 visitors a year). The pressures associated with mass tourism extended well beyond visitor numbers alone, generating a cascade of secondary disturbances. These included the proliferation of kiosks, with attendant increases in noise and artificial lighting; the construction of additional mooring facilities and piers; the introduction of more vehicles and vans to service tourist demand; and a marked rise in litter accumulation.

For example, the use of pineapple shells to serve ice cream or drinks should be abolished or better managed. Wandering tourists often toss the shells in the countryside, reasoning that since they are organic, it is fine to do so, without realising that they are helping the population of rats to increase and then their large numbers become a serious threat to local fauna and even flora in the non-touristic season when such anthropogenic-derived food is not available anymore. Beach cleaning organised by central authorities is effective. Still, a few pineapple shells remain on site in inaccessible pockets, rock depressions, or far from the beach where cleaners do not operate.

Collectively, these impacts have contributed to significant ecological stress and a progressive erosion of the island’s natural character. Fortunately, following sustained pressure on the relevant authorities, a regulated booking system was introduced in the summer of 2025 to manage visitor flows to Comino. This intervention has proven highly effective in reducing visitor density, capping attendance at 4,000 per designated time slot. On the busiest days, numbers were further contained, with peak attendance reaching no more than 3,800 visitors. Average participation per slot stabilised at approximately 2,000 persons, reflecting a significant improvement in crowd management compared to previous years. Notably, during the peak tourist months of July and August, only 34 out of 189 monitored slots exceeded 3,000 visitors, underscoring the system’s success in mitigating excessive pressures on the island’s fragile ecosystems. This measure represents a critical step toward balancing tourism demand with the long-term sustainability of Comino’s natural and cultural heritage.

A significant challenge now looms over Comino, arising from plans to reconstruct a larger, more luxurious hotel to replace the existing facility, coupled with the proposed sale of the hotel’s bungalows to private owners. The proposed conversion of Santa Marija Bay into a small private residential area, composed of some 16 villas replacing the old hotel’s bungalows, would definitely increase the risk of urbanisation at this quasi-pristine Natura 2000 site. Such development would trigger multiple secondary pressures, including the inevitable construction of tarmacked access roads from Santa Marija Bay to the hotel and potentially to the Blue Lagoon, increased vehicular traffic, and the gradual expansion of ancillary infrastructure. Anticipated facilities such as a food market, basic retail outlets, a medical point, entertainment venues, and tourist-oriented shops would further intensify human presence and landscape modification. The likely establishment of a small dock to service new residents would introduce additional marine and coastal disturbance. Collectively, these incremental developments would contribute to a progressive and cumulative deterioration of the site’s ecological character.

This trajectory underscores the urgent need for careful planning and stringent regulation to safeguard the island’s biodiversity and natural heritage, while preserving Comino’s longstanding character as a tranquil refuge, where residents can periodically withdraw from the densely populated, highly urbanised, and often chaotic environments of mainland Malta and Gozo.

## Conclusion

This study is the first comprehensive synthesis concerning the terrestrial vascular flora of the Comino archipelago. Our botanical surveys, supplemented by an exhaustive review of historical floristic data, provide a workable checklist of vascular plants recorded and currently occurring on the Comino archipelago. This work confirms the presence (historic and present) of at least 490 taxa, of which 328 have been observed across 26 surveys conducted between February 2019 and April 2025. Of these, 58 species are strictly protected, 21 are endemic or subendemic taxa, and several more are rare and threatened in the Maltese archipelago. These results underscore once again the key role of small Mediterranean islands as refuges for coastal biodiversity (Médail 2017, 2022).

However, the main island of Comino has undergone significant anthropogenic upheavals throughout its history, where it was deserted at one phase in time, occupied by over 100 resident farmers in the early 20^th^ C., and, over the last 20 years, visited by an average of 4,000–5,000 tourists per day in summertime (peaking at about 12,000 in July and August of 2024). It is undeniable that this number of tourists is unsustainable for the limited small beaches and bathing areas on Comino Island, resulting in inevitable mass trampling and litter. This disturbance is not limited only to coastal habitats but also to the strictly protected sand dune of Santa Marija, where bathers trespass into the cordoned area to sunbathe on the dune, despite the signs requesting the safeguarding of this small and critically threatened arenous habitat.

Improper afforestation practices and the introduction of alien species constitute additional anthropogenic pressures contributing to the decline of native flora. When sustained over extended periods, these interventions have driven significant changes in the floristic diversity across the archipelago. In recent years, limited mitigation efforts have been initiated, including the targeted management of invasive *Acacia* spp. by central authorities and the regulation of visitor numbers at the Blue Lagoon, where a pilot intervention was implemented in 2025.

Further conservation-oriented measures include increased patrolling of densely visited areas by environmental wardens (e.g. the Malta Rangers Unit), stricter enforcement of ecological regulations, reductions in vehicular access and kiosk proliferation, and a broader policy emphasis on biodiversity protection. Additionally, an eco-sensitive management plan for the demolition of the existing hotel premises must be implemented and adhered to, without further degrading natural habitats.

The Comino archipelago is one of the few remaining sites in the Maltese Islands where a coastal eco-complex can be safeguarded through sustainable management. The implementation of a coherent, evidence-based habitat management strategy is essential for enhancing ecosystem resilience and ensuring the long-term survival of threatened plant species that persist here, largely insulated from the intense demographic and pollution pressures characteristic of mainland Malta and Gozo.

Our present synthesis is intended to provide a foundational ecological resource for stakeholders engaged in the conservation and management of Comino’s natural environment. More broadly, our findings underscore the critical importance of protecting these last vestiges of Malta’s relatively undisturbed habitats and associated biodiversity. One has to appreciate that the Maltese flora reflects a blend of Sicilian, Aegean–Eastern Mediterranean, and Tunisian–North African plant communities, forming a distinctive assemblage found nowhere else. While much of this natural heritage has been degraded on the main islands, it remains remarkably well preserved on the Comino archipelago, which was designated a Natura 2000 site for this reason and to safeguard it for the benefit of future generations.

## Additional information

### Use of AI

No use of AI was reported.

### Author ORCIDs

Stephen Mifsud https://orcid.org/0000-0002-0319-300X

### Data availability

All of the data that support the findings of this study are available in the main text or Supplementary Information.

## Supplementary materials

Supplementary material 1 Appendices Data type docx https://binary.pensoft.net/file/1587315This dataset is made available under the Open Database License (http://opendatacommons.org/licenses/odbl/1.0/). The Open Database License (ODbL) is a license agreement intended to allow users to freely share, modify, and use this Dataset while maintaining this same freedom for others, provided that the original source and author(s) are credited. Stephen Mifsud
