# Supplementary material for: The vascular flora of the Comino archipelago (Maltese Islands)
Source: PhytoKeys. 2026 Apr 8;272:217–82. doi: 10.3897/phytokeys.272.184198 (PMC13084267; doi:10.3897/phytokeys.272.184198)
Supplement: Supplementary material 1 — Appendices [file phytokeys-272-217_article-184198__-s001.docx]

**Appendix A**

Published taxa that are obsolete, misapplied, synonymous or ambiguous and their corresponding accepted taxon.

Table A1: Obsolete, synonymous, misapplied or erratic taxa cited in published old literature and which have been rejected, and the corresponding treatment of currently accepted taxon used in this work, giving the rationale (“Rtn” in column 4) with the following legend: syn synonym of the currently used taxon; mis misidentification/misapplied taxon in the past; end tentative taxon used for an endemic species described later.

| Cited taxon in literature | Currently used taxon | First citation | Rtn |
| --- | --- | --- | --- |
| *Ailanthus glutinosa* | *Ailanthus altissima* | Sommier and Caruana Gatto (1915) | syn |
| *Allium ampeloprasum* | *Allium polyanthum* | Sommier and Caruana Gatto (1915) | mis |
| *Allium ampeloprasum* var. *melitense* | *Allium polyanthum* | Sommier and Caruana Gatto (1915) | syn |
| *Allium commutatum* | *Allium polyanthum* | Borg (1927) | mis |
| *Allium melitense* | *Allium polyanthum* | Haslam et al (1977) | syn |
| *Allium parciflorum* | *Allium lojacanoi* | Sommier and Caruana Gatto (1915) | end |
| *Allium subhirsutum* | *Allium subvillosum* | Borg (1927) | syn |
| *Althaea hirsuta* | *Malva setigera* | Duthie (1874-75) | syn |
| *Alyssum maritimum* | *Lobularia maritima* | Sommier and Caruana Gatto (1915) | syn |
| *Anagallis arvensis* | *Lysimachia arvensis* | Duthie (1874-75) | syn |
| *Anchusa italica* | *Anchusa azurea* | Sommier and Caruana Gatto (1915) | syn |
| *Andropogon hirtus* | *Hyparrhenia hirta* | Sommier and Caruana Gatto (1915) | syn |
| *Andropogon pubescens* | *Hyparrhenia hirta* | Borg (1927) | syn |
| *Anthyllis tetraphylla* | *Tripodion tetraphyllum* | Sommier and Caruana Gatto (1915) | syn |
| *Antirrhinum majus* | *Antirrhinum tortuosum* | Borg (1927) | mis |
| *Antirrhinum orontium* | *Misopates orontium* | Sommier and Caruana Gatto (1915) | syn |
| *Arenaria geniculata* | *Rhodalsine geniculata* | Duthie (1874-75) | syn |
| *Arthrocnemum glaucum* | *Arthrocaolon macrostachyum* | Sommier and Caruana Gatto (1915) | syn |
| *Asperula longiflora* | *Cynanchica aristata* subsp. *scabra* | Sommier and Caruana Gatto (1915) | syn |
| *Atractylis gummifera* | *Carlina gummifera* | Sommier and Caruana Gatto (1915) | syn |
| *Bartsia trixago* var. *versicolor* | *Bellardia trixago* | Duthie (1874-75) | syn |
| *Beta maritima* | *Beta vulgaris* subsp. *maritima* | Sommier and Caruana Gatto (1915) | syn |
| *Blackstonia perfoliata* | *Blackstonia acuminata* | Duthie (1874-75) | mis |
| *Brachypodium distachyon* | *Brachypodium hybridum* | Sommier and Caruana Gatto (1915) | mis |
| *Brachypodium plukenetti* | *Brachypodium retusum* | Duthie (1874-75) | syn |
| *Brachypodium ramosum* | *Brachypodium retusum* | Sommier and Caruana Gatto (1915) | syn |
| *Brassica adpressa* | *Hirschfeldia incana* | Sommier and Caruana Gatto (1915) | syn |
| *Brassica sinapistrum* | *Sinapis arvensis* | Sommier and Caruana Gatto (1915) | syn |
| *Bromus maximus* | *Bromus madritensis* | Sommier and Caruana Gatto (1915) | syn |
| *Buphthalmum spinosum* | *Pallenis spinosa* | Duthie (1874-75) | syn |
| *Bupleurum subovatum* | *Bupleurum lancifolium* | Sommier and Caruana Gatto (1915) | syn |
| *Carduus marmoratus* | *Carduus pycnocephalus* subsp. *arabicus* | Sommier and Caruana Gatto (1915) | syn |
| *Callipeltis muralis* | *Galium murale* | Sommier and Caruana Gatto (1915) | syn |
| *Capparis spinosa* | *Capparis orientalis / C. spinosa* subsp. *rupestris* | Sommier and Caruana Gatto (1915) | syn |
| *Carlina corymbosa* | *Carlina involucrata* | Borg (1927) | mis |
| *Carlina gummifera* | *Atractylis gummifer* | Sommier and Caruana Gatto (1915) | syn |
| *Carpobrotus edulis* | *Carpobrotus acinaciformis* | Camilleri et al (2012) | mis |
| *Caruelia arabica* | *Ornithogalum arabicum* | Sommier and Caruana Gatto (1915) | syn |
| *Catapodium loliaceum* | *Catapodium marinum* | Sommier and Caruana Gatto (1915) | syn |
| *Catapodium siculum* | *Desmazeria pignatti* | Duthie (1874-75) | end |
| *Chlora perfoliata* | *Blackstonia perfoliata* | Duthie (1874-75) | syn |
| *Cineraria maritima* | *Jacobaea maritima* subsp. *sicula* | Duthie (1874-75) | syn |
| *Colchicum bertolonii* | *Colchicum cupanii* | Sommier and Caruana Gatto (1915) | syn |
| *Convolvulus cneorum* | *Convolvulus oleifolius* | Duthie (1874-75) | mis |
| *Convolvulus doneatus* | n/a | Borg (1927) | ? |
| *Conyza ambigua* | *Erigeron bonariensis* | Sommier and Caruana Gatto (1915) | syn |
| *Conyza bonariensis* | *Erigeron bonariensis* | Duthie (1874-75) | syn |
| *Conyza tenorii* | *Phagnalon rupestre* | Duthie (1874-75) | syn |
| *Crepis bulbosa* | *Sonchus bulbosa* | Sommier and Caruana Gatto (1915) | syn |
| *Crucianella rupestris* | *Crucianella maritima* | Borg (1927) | syn |
| *Cuscuta alba* | *Cuscuta epithymum* | Sommier and Caruana Gatto (1915) | syn |
| *Daucus gingidium* | *Daucus carota* subsp. *drepanensis / D. rupestris* s.l. | Sommier and Caruana Gatto (1915) | syn |
| *Desmazeria sicula* | *Desmazeria pignatti* | Haslam et al (1977) | end |
| *Erophila verna* | *Hornungia procumbens* subsp. *revelierei* | Duthie (1874-75) | mis |
| *Erophila vulgaris* | *Draba verna* | Duthie (1874-75) | mis |
| *Ervilia sativa* | *Vicia ervilia* | Borg (1927) | syn |
| *Erythraea centaurium* | *Centaurium erythraea* | Sommier and Caruana Gatto (1915) | syn |
| *Erythraea pulchellum* | *Centaurium pulchellum* | Sommier and Caruana Gatto (1915) | syn |
| *Euphorbia peploides* | *Euphorbia peplus* | Duthie (1874-75) | syn |
| *Euphorbia pinea* | *Euphorbia segetalis* var. *pinea* | Duthie (1874-75) | syn |
| *Euphorbia spinosa* | *Euphorbia melitensis* | Sommier and Caruana Gatto (1915) | end |
| *Evax pygmaea* | *Filago pygmaea* | Duthie (1874-75) | syn |
| *Fedia cornucopiae* | *Valeriana graciliflora* | Sommier and Caruana Gatto (1915) | syn |
| *Ferula communis* | *Ferula melitensis* | Sommier and Caruana Gatto (1915) | end |
| *Filago gussonei* | *Filago pyramidata* s.l. | Sommier and Caruana Gatto (1915) | syn |
| *Filago prostrata* | *Filago pyramidata* s.l. | Sommier and Caruana Gatto (1915) | syn |
| *Filago spathulata* | *Filago pyramidata* s.l. | Sommier and Caruana Gatto (1915) | syn |
| *Frankenia intermedia* | *Frankenia hirsuta* | Duthie (1874-75) | syn |
| *Frankenia laevis var. hirsuta* | *Frankenia hirsuta* | Sommier and Caruana Gatto (1915) | syn |
| *Galium saccharatum* | *Galium verrucosum* | Sommier and Caruana Gatto (1915) | syn |
| *Gastridium lendiferum* | *Gastridium ventricosum* | Sommier and Caruana Gatto (1915) | syn |
| *Gladiolus segetum* | *Gladiolus italicus* | Sommier and Caruana Gatto (1915) | syn |
| *Hedypnois cretica* | *Hedypnois rhagadioloides* | Haslam et al (1977) | mis |
| *Hedypnois mauritanica* | *Hedypnois rhagadioloides* | Duthie (1874-75) | syn |
| *Hedypnois polymorpha* | *Hedypnois rhagadioloides* | Sommier and Caruana Gatto (1915) | syn |
| *Hedysarum pallens* | *Sulla spinosissimum* | Sommier and Caruana Gatto (1915) | syn |
| *Helminthia echioides* | *Picris echioides* | Sommier and Caruana Gatto (1915) | syn |
| *Hippocrepis ciliata* | *Hippocrepis multisiliquosa* | Duthie (1874-75) | syn |
| *Hippocrepis unisiliquosa* | *Hippocrepis biflora* | Sommier and Caruana Gatto (1915) | syn |
| *Hutchinsia procumbens* | *Hornungia procumbens* subsp. *revelierei* | Sommier and Caruana Gatto (1915) | syn |
| *Hymenolobus revelieri* subsp. *sommieri* | *Hornungia procumbens* subsp. *revelierei* | Haslam et al (1977) | syn |
| *Hyoseris lucida* | *Hyoseris frutescens* | Borg (1927) | end |
| *Inula crithmoides* | *Limbarda crithmoides* | Duthie (1874-75) | syn |
| *Inula graveolens* | *Dittrichia graveolens* | Sommier and Caruana Gatto (1915) | syn |
| *Inula viscosa* | *Dittrichia viscosa* | Sommier and Caruana Gatto (1915) | syn |
| *Iris sisyrinchium* | *Moraea sisyrinchium* | Sommier and Caruana Gatto (1915) | syn |
| *Jasonia glutinosa* | *Chiliadenus bocconei* | Borg (1927) | end |
| *Juncus bufonius* | *Juncus hybridus* | Sommier and Caruana Gatto (1915) | mis |
| *Juncus bufonius var. sorrentini* | *Juncus hybridus* | Borg (1927) | mis |
| *Koeleria phleoides* | *Rostraria cristata* | Duthie (1874-75) | syn |
| *Kopsia mutelii* | *Phelipanche ramosa* | Sommier and Caruana Gatto (1915) | syn |
| *Lactuca spinosa* | *Lactuca serriola* | Duthie (1874-75) | mis |
| *Lathyrus articulatus* | *Lathyrus clymenum* | Sommier and Caruana Gatto (1915) | syn |
| *Lavatera cretica* | *Malva multiflora* | Haslam et al (1977) | syn |
| *Lepturus incurvatus* | *Parapholis incurva* | Duthie (1874-75) | syn |
| *Limonium graecum* | *Limonium melitense* | Haslam et al (1977) | end |
| *Linaria reflexa* | *Linaria pseudolaxiflora* | Duthie (1874-75) | end |
| *Linum gallicum* | *Linum trigynum* | Duthie (1874-75) | syn |
| *Lithospermum apulum* | *Neatostema apulum* | Sommier and Caruana Gatto (1915) | syn |
| *Lotus creticus* | *Lotus cytisoides* | Duthie (1874-75) | syn |
| *Lotus pusillus* | *Lotus halophilus* | Duthie (1874-75) | syn |
| *Malva microcarpa* | *Malva parviflora* | Sommier and Caruana Gatto (1915) | syn |
| *Medicago denticulata* | *Medicago polymorpha* | Sommier and Caruana Gatto (1915) | syn |
| *Medicago infestus* | *Melilotus sulcatus* | Sommier and Caruana Gatto (1915) | syn |
| *Medicago recta* | *Medicago minima* | Duthie (1874-75) | syn |
| *Medicago sulcatus* subsp. *infestus* | *Melilotus sulcatus* | Sommier and Caruana Gatto (1915) | syn |
| *Melilotus compacta* | *Melilotus segetalis* | Duthie (1874-75) | syn |
| *Melilotus messanensis* | *Melilotus siculus* | Sommier and Caruana Gatto (1915) | syn |
| *Muscari comosum* | *Leopoldia comosa* | Sommier and Caruana Gatto (1915) | syn |
| *Narcissus serotinus* | *Narcissus deficiens* | Borg (1927) | mis |
| *Ononis mollis* | *Ononis reclinata* | Duthie (1874-75) | syn |
| *Ononis natrix* subsp. *ramosissima* | *Ononis ramosissima* | Duthie (1874-75) | syn |
| *Ononis viscosa* subsp. *sieberi* | *Ononis sieberi* | Sommier and Caruana Gatto (1915) | syn |
| *Onopordum sibthorpianum* | *Onopordum argolicum* | Sommier and Caruana Gatto (1915) | syn |
| *Orchis collina* | *Anacamptis collina* | Lanfranco (2004) | syn |
| *Orchis lactea* | *Neotinea lactea* | Sommier and Caruana Gatto (1915) | syn |
| *Orobanche mutelii* | *Orobanche nana* | Sommier and Caruana Gatto (1915) | syn |
| *Orobanche picris* | *Orobanche picridis-hieracioidis* | Sommier and Caruana Gatto (1915) | syn |
| *Orobanche ramosa* | *Orobanche nana* | Sommier and Caruana Gatto (1915) | syn |
| *Orobanche versicolor* | *Orobanche pubescens* | Sommier and Caruana Gatto (1915) | syn |
| *Oxalis cernua* | *Oxalis pes-caprae* | Sommier and Caruana Gatto (1915) | syn |
| *Parietaria officinalis* | *Parietaria judaica* | Sommier and Caruana Gatto (1915) | syn |
| *Phagnalon graecum* | *Phagnalon rupestre* subsp. *graecum* | Sommier and Caruana Gatto (1915) | syn |
| *Phagnalon tenori[e]i* | *Phagnalon rupestre* subsp. *graecum* | Sommier and Caruana Gatto (1915) | syn |
| *Phragmites communis* | *Phragmites australis* | Sommier and Caruana Gatto (1915) | syn |
| *Picridium vulgare* | *Reichardia picroides* | Sommier and Caruana Gatto (1915) | syn |
| *Picris echioides* | *Helminthotheca echioides* | Sommier and Caruana Gatto (1915) | syn |
| *Pinardia coronaria* | *Glebionis coronaria* | Sommier and Caruana Gatto (1915) | syn |
| *Plantago bombycina* | *Plantago weldenii* | Sommier and Caruana Gatto (1915) | syn |
| *Poa rigida* | *Catapodium rigidum* | Duthie (1874-75) | syn |
| *Polycarpon alsinifolium* | *Polycarpon tetraphyllum* subsp. *alsinifolium* | Duthie (1874-75) | syn |
| *Polycarpon diphyllum* | *Polycarpon tetraphyllum* subsp. *diphyllum* | Lanfranco (2004) | syn |
| *Poterium sanguisorba* | *Sanguisorba minor* | Sommier and Caruana Gatto (1915) | syn |
| *Pseudorlaya pumila* | *Daucus pumilus* | Borg (1927) | syn |
| *Psoralea bituminosa* | *Bituminaria bituminosa* | Sommier and Caruana Gatto (1915) | syn |
| *Ptychotis pusilla* | *Ammoides pusilla* | Sommier and Caruana Gatto (1915) | syn |
| *Putoria calabrica* | *Plocama calabrica* | Haslam et al (1977) | syn |
| *Ranunculus aquatilis* subsp. *diversifolis* var. *baudotii* | *Ranunculus saniculifolius* | Borg (1927) | mis |
| *Rhodalsine geniculata* | *Minuartia geniculata* | Duthie (1874-75) | syn |
| *Romulea melitensis* | *Romulea variicolor* | Sommier and Caruana Gatto (1915) | end |
| *Romulea ramiflora* | *Romulea variicolor* | Borg (1927) | end |
| *Ruta bracteosa* | *Ruta chalepensis* | Duthie (1874-75) | syn |
| *Salsola tragus* | *Salsola kali* | Sommier and Caruana Gatto (1915) | syn |
| *Satureja nepeta* | *Clinopodium nepeta* | Sommier and Caruana Gatto (1915) | syn |
| *Scilla autumnalis* | *Prospero autumnale* complex | Sommier and Caruana Gatto (1915) | syn |
| *Scleropoa rigida* | *Catapodium rigidum* | Sommier and Caruana Gatto (1915) | syn |
| *Scorpiurus subvillosus* | *Scorpiurus muricatus* | Duthie (1874-75) | syn |
| *Sedum heptapetalum* | *Sedum caeruleum* | Sommier and Caruana Gatto (1915) | syn |
| *Sedum sediforme* | *Petrosedum sediforme* | Borg (1927) | syn |
| *Senecio bicolor* | *Jacobaea maritima* subsp. *sicula* | Duthie (1874-75) | syn |
| *Senecio vernus* | *Senecio leucanthemifolius* | Duthie (1874-75) | mis |
| *Serapias occulta* | *Serapias parviflora* | Sommier and Caruana Gatto (1915) | syn |
| *Seriola aethnensis* | *Hypochoeris achyrophorus* | Duthie (1874-75) | syn |
| *Serrafalcus mollis* | *Bromus hordeaceus* | Sommier and Caruana Gatto (1915) | syn |
| *Silene inflata* | *Silene vulgaris* | Sommier and Caruana Gatto (1915) | syn |
| *Sonchus levis* | *Sonchus oleraceus* | Duthie (1874-75) | syn |
| *Stachys hirta* | *Stachys ocymastrum* | Sommier and Caruana Gatto (1915) | syn |
| *Statice reticulata* | *Limonium melitense* | Sommier and Caruana Gatto (1915) | end |
| *Statice virgata* | *Limonium virgatum* | Sommier and Caruana Gatto (1915) | syn |
| *Stipa capensis* | *Stipellula capensis* | Haslam et al (1977) | syn |
| *Stipa tortilis* | *Stipellula capensis* | Duthie (1874-75) | syn |
| *Suaeda fruticosa* | *Suaeda vera* | Sommier and Caruana Gatto (1915) | syn |
| *Tetragonolobus purpureus* | *Lotus tetragonolobus* | Sommier and Caruana Gatto (1915) | syn |
| *Thrincia tuberosa* | *Leontodon tuberosus* | Sommier and Caruana Gatto (1915) | syn |
| *Trifolium agrarium* | *Trifolium campestre* | Sommier and Caruana Gatto (1915) | syn |
| *Trigonella monspeliaca* | *Medicago monspeliaca* | Duthie (1874-75) | syn |
| *Urginea scilla* | *Drimia pancration* | Duthie (1874-75) | syn |
| *Vicia angustifolia* | *Vicia sativa* subsp. *nigra* | Borg (1927) | syn |
| *Vulpia membranacea* | *Vulpia ciliata* | Borg (1927) | mis |
| *Vulpia uniglumis* | *Vulpia fasciculata* | Sommier and Caruana Gatto (1915) | mis |
| *Zannichellia palustris* | *Zannichellia melitensis* | Sommier and Caruana Gatto (1915) | end |

**Appendix B**

Plants that have been recorded specifically from Cominotto Island and the three islets of the Comino Archipelago (Ħaġra ta’ Taħt il-Mazz, il-Ħaġra l-Kbira, and il-Ħaġra z-Żgħira).

Table B Plants observed in Cominotto (132 species), the islets of il-Ħaġra ta’ Taħt il-Mazz [**ĦM**] (32 spp), il-Ħaġra l-Kbira [**ĦK**] (53 spp), and il-Ħaġra z-Żgħira [**ĦŻ**] (20 spp), from surveys carried out on the dates dates 04/06/2020 [**6a, 6b**], 11/10/2020 [**7**], 26/03/2021 [**10**], 15/04/2021 [**12a, 12b, 12c**], 04/05/2021 [**14**], 24/11/2024 [**20a, 20b**] and 04/04/2025 [**21**]. Species shaded in green are endemic or subendemic. This table also includes the estimated frequencies on each corresponding island/islet using the following legend: (RR) very rare; (R) rare, (I) scarce, fragmented or frequent in a few places, (C) frequent/common, (CC) very common throughout. The respective frequency of each species on Comino main island is given in the last column for comparison purposes; those absent on the main island are denoted by ‘-‘.

| Species | Visit when first observed on the respective island or islet | Frequency on Cominotto Island | Frequency at Ħaġra taħt il-Mazz | Frequency at Ħaġra l-Kbira ta' bejn il-Kmienen | Frequency at Ħaġra ż-Żgħira ta' bejn il-Kmienen | Frequency on the Comino mainland |
| --- | --- | --- | --- | --- | --- | --- |
| *Agave americana* var. *americana* | 6a | RR |  |  |  | R |
| *Agave sisalana* | 6a | RR |  |  |  | RR |
| *Ajuga iva* s.l. | 7 | R |  |  |  | I |
| *Allium lojaconoi* | 6a, 14 | RR |  | I |  | R |
| *Allium polyanthum* | 10, 20a | R |  | RR |  | RR |
| *Anacamptis coriophora* subsp. *fragrans* | 21 | RR |  |  |  | RR |
| *Anacamptis pyramidalis* subsp*. pyramidalis* | 12a | RR |  |  |  | R |
| *Anthemis secundiramea* | 7, 14, 12c | R |  | CC | I | R |
| *Anthyllis hermanniae* subsp. *melitensis* | 6a, 6b | CC | RR |  |  | CC |
| *Anthyllis vulneraria* subsp. *maura* | 12a, 14 | R |  | R |  | R |
| *Arisarum vulgare* | 7, 20a | I |  | I |  | I |
| *Arthrocaulon macrostachyum* | 14, 12c |  |  | RR | CC | R |
| *Asparagus aphyllus* | 6a, 6b, 14, 6b | R | RR | R | RR | R |
| *Asteriscus aquaticus* | 6a | I |  |  |  | CC |
| *Atractylis gummifera* | 6a | I |  |  |  | I |
| *Bituminaria bituminosa* | 12a | R |  |  |  | RR |
| *Blackstonia acuminata* | 6a, 12b, 14 | R | RR | R |  | I |
| *Brachypodium hybridum* | 10, 6b | R | R |  |  | R |
| *Brachypodium retusum* | 6a | I |  |  |  | R |
| *Bromus fasciculatus* | 21, 12b, 14 | R | R | RR |  | I |
| *Bromus madritensis* | 12a | RR |  |  |  | I |
| *Bromus rigidus* | 12a | RR |  |  |  | RR |
| *Capparis spinosa* subsp. *rupestris* (= *C. orientalis*) | 6a, 6b, 14, 6b | R | RR | R | R | I |
| *Carlina involucrata* | 7 | CC |  |  |  | I |
| *Carthamus lanatus* | 7 | RR |  |  |  | RR |
| *Catapodium marinum* | 21, 6b | R | RR |  |  | RR |
| *Catapodium pauciflorum* | 12a, 12b, 14 | R | R | R |  | R |
| *Catapodium rigidum* | 10, 12b, 14 | RR | RR | I |  | I |
| *Centaurium erythraea* | 6a, 12b, 14 | R | R | R |  | I |
| *Centaurium pulchellum* | 12a, 14 | RR |  | R |  | C |
| *Centaurium tenuiflorum* | 6a, 12b | RR | RR |  |  | I |
| *Chenopodiastrum murale* | 7 | R |  |  |  | R |
| *Cichorium spinosum* | 7 | RR |  |  |  | RR |
| *Convolvulus althaeoides* | 7 | RR |  |  |  | R |
| *Convolvulus oleifolius* | 10, 14 | RR |  | CC |  | I |
| *Coronilla scorpioides* | 10 | R |  |  |  | R |
| *Crithmum maritimum* | 6a | R |  |  |  | R |
| *Crucianella maritima* | 6a, 6b, 14 | R | R | C |  | R |
| *Cuscuta epithymum* | 10, 14 | R |  | R |  | R |
| *Cynara cardunculus* | 6a | R |  |  |  | I |
| *Daucus carota* subsp. *commutatus* var. *tenuisectus* | 7 | R |  |  |  | R |
| *Daucus rupestris* s.l. | 6a, 6b, 14, 6b | R | CC | C | I | I |
| *Desmazeria pignatti* | 10, 6b, 6b | I | R |  | RR | I |
| *Dittrichia viscosa* | 6a | R |  |  |  | I |
| *Drimia pancration* | 6a, 6b, 14, 6b | C | R | I | I | R |
| *Echium arenarium* | 10 | R |  |  |  | R |
| *Echium parviflorum* | 7 | RR |  |  |  | RR |
| *Erodium malacoides* | 10 | RR |  |  |  | I |
| *Euphorbia exigua* | 10 | C |  |  |  | I |
| *Euphorbia melitensis* | 6a | I |  |  |  | CC |
| *Euphorbia peplus* | 7 | I |  |  |  | I |
| *Euphorbia segetalis* var*. pinea* | 6a, 14 | C |  | R |  | C |
| *Ferula melitensis* | 6a | I |  |  |  | C |
| *Ficus carica* | 6a | RR |  |  |  | R |
| *Filago pygmaea* | 21 | R |  |  |  | R |
| *Filago pyramidata* s.l. | 10 | RR |  |  |  | R |
| *Frankenia hirsuta* | 7, 12c | RR |  |  | R | R |
| *Frankenia pulverulenta* | 21 | RR |  |  |  | R |
| *Galactites tomentosus* | 10 | RR |  |  |  | R |
| *Galium murale* | 7 | R |  |  |  | I |
| *Hedypnois rhagadioloides* | 12a | RR |  |  |  | RR |
| *Hippocrepis multisiliquosa* | 10 | RR |  |  |  | R |
| *Hippocrepis unisiliquosa* | 10 | I |  |  |  | RR |
| *Hornungia procumbens* subsp. *revelierei* | 21 | RR |  |  |  | RR |
| *Hypericum aegyptiacum* | 6a, 14 | RR |  | CC |  | I |
| *Hypochaeris achyrophorus* | 14 |  |  | R |  | RR |
| *Jacobaea maritima* subsp. *sicula* | 6a, 14 | I |  | RR |  | I |
| *Juncus hybridus* | 14 |  |  | RR |  | R |
| *Leontodon tuberosus* | 20c | RR |  |  |  | R |
| *Leopoldia comosum* | 10 | RR |  |  |  | RR |
| *Limbarda crithmoides* | 6a, 6b, 14, 6b | I | CC | I | I | I |
| *Limonium melitense / L. zeraphae* | 6a, 6b, 14, 6b | R | R | R | I | I |
| *Limonium virgatum* | 6a, 6b, 14, 6b | I | C | C | C | I |
| *Linaria pseudolaxiflora* | 10 | RR |  |  |  | R |
| *Linum strictum* | 12a, 14 | R |  | R |  | R |
| *Linum trigynum* | 10, 14 | RR |  | I |  | I |
| *Lobularia maritima* | 10 | RR |  |  |  | RR |
| *Lotus cytisoides* | 10 | RR |  |  |  | R |
| *Lotus edulis* | 12a, 12b, 20 | R | RR | RR |  | RR |
| *Lygeum spartum* | 12c |  |  |  | R | RR |
| *Lysimachia arvensis* | 6a, 20a | R |  | RR |  | C |
| *Lysimachia loeflingii* | 6a | C |  |  |  | RR |
| *Malva setigera* | 21 | RR |  |  |  | RR |
| *Matthiola incana* subsp. *melitensis* | 6b |  | I |  |  | RR |
| *Medicago littoralis* | 10 | RR |  |  |  | RR |
| *Medicago polymorpha* | 12b |  | RR |  |  | RR |
| *Melilotus indicus* | 10, 14, 12c | I |  | R | I | RR |
| *Mesembryanthemum nodiflorum* | 6a, 12b, 14, 12c | R | RR | C | C | I |
| *Micromeria microphylla* | 20c | RR |  |  |  | R |
| *Narcissus deficiens* | 7, 20a | RR |  | RR |  | RR |
| *Narcissus tazetta* s.l. | 10, 14 | R |  | I |  | C |
| *Olea europaea* var. *silvestris* | 20c | RR |  |  |  | R |
| *Ononis reclinata* | 21, 14 | RR |  | R |  | RR |
| *Ononis sieberi* | 10 | R |  |  |  | RR |
| *Ophrys melitensis* | 21 | RR |  |  |  | RR |
| *Ophrys speculum* | 21 | RR |  |  |  | RR |
| *Orobanche cernua* | 12a, 12b, 14 | RR | RR | RR |  | R |
| *Orobanche* cf. *minor* | 12a | RR |  |  |  | RR |
| *Oxalis pes-caprae* | 10 | RR |  |  |  | I |
| *Oxalis pes-caprae* var. *pleniflora* | 10 | RR |  |  |  | R |
| *Pallenis spinosa* | 12a | RR |  |  |  | RR |
| *Parapholis incurva* | 21, 12b, 14, 12c | RR | RR | RR | I | R |
| *Periploca angustifolia* | 6a | R |  |  |  | I |
| *Phagnalon graecum* subsp. *ginzbergeri* | 14 |  |  | RR |  | RR |
| *Phagnalon rupestre* | 10 | RR |  |  |  | R |
| *Pistacia lentiscus* | 6a, 6b, 20a | I | RR | RR |  | CC |
| *Polypogon maritimus* | 21, 12b, 14 | R | R | R |  | C |
| *Polypogon monspeliensis* | 21 | RR |  |  |  | - |
| *Polypogon subspathaceus* | 12b, 14 |  | I | R |  | C |
| *Prasium majus* | 10 | RR |  |  |  | R |
| *Prospero autumnale* | 7, 20a | I |  | R |  | I |
| *Reichardia picroides* | 10, 20a | I |  | RR |  | R |
| *Romulea variicolor* | 21, 14 | RR |  | I |  | R |
| *Rostraria cristata* | 6b, 14 |  | RR | RR |  | R |
| *Ruta chalepensis* | 6a | I |  |  |  | I |
| *Sagina procumbens* | 21 | R |  |  |  | - |
| *Salsola melitensis* | 6a, 6b, 6b | R | I |  | R | I |
| *Schoenus nigricans* | 10 | RR |  |  |  | RR |
| *Scorpiurus muricatus* | 10, 20a | I |  | RR |  | I |
| *Sedum litoreum* | 10, 14 | R |  | I |  | R |
| *Sedum rubens* | 10 | RR |  |  |  | RR |
| *Senecio leucanthemifolius* | 10, 6b, 20, 6b | RR | R | RR | R | RR |
| *Senecio pygmaeus* | 20c | R |  |  |  | R |
| *Sideritis romana* | 10 | RR |  |  |  | R |
| *Silene sedoides* | 10, 6b, 14, 6b | I | R | I | R | I |
| *Sinapis alba* | 10 | RR |  |  |  | RR |
| *Sonchus bulbosus* | 10 | R |  |  |  | R |
| *Sonchus oleraceus* s.l. | 10, 20a, 20b | R |  | RR | RR | R |
| *Sonchus tenerrimus* | 20c |  |  |  |  | RR |
| *Spergularia diandra* | 14, 12c |  |  | R | R | RR |
| *Sporobolus pungens* | 6a | RR |  |  |  | RR |
| *Sulla spinosissimum* | 10 | RR |  |  |  | R |
| *Teucrium flavum* | 6a | R |  |  |  | I |
| *Teucrium fruticans* | 7 | C |  |  |  | C |
| *Theligonum cynocrambe* | 10 | R |  |  |  | RR |
| *Thymbra capitata* | 6a | I |  |  |  | C |
| *Tordylium apulum* | 10 | I |  |  |  | R |
| *Trifolium scabrum* | 10 | R |  |  |  | R |
| *Triticum durum* | 10 | RR |  |  |  | RR |
| *Urospermum picroides* | 21 | RR |  |  |  | R |
| *Valantia muralis* | 10, 6b, 14 | CC | RR | I |  | C |
| *Vulpia ciliata* | 10 | R |  |  |  | I |

**Appendix C**

Species recorded on Comino archipelago which are ecologically sensitive and protected by Maltese Legislation.

Table C Species that are Endemic, Subendemic, Protected by Law or/and listed in the Red Data Book of the Maltese Islands, hence indicating the most sensitive species occurring on the Comino Archipelago.

| **Species** | **Plant Family** | Frequency on Comino from surveys | Endemism | Local legislative protection | Listed in the RDB of the Maltese Islands |
| --- | --- | --- | --- | --- | --- |
| *Allium lojaconoi* | Amaryllidaceae | R | Subendemic | Yes | Yes |
| *Anacamptis pyramidalis* subsp. *urvilleana* | Orchidaceae | Not observed | Endemic | Yes | Yes |
| *Anthyllis hermanniae* subsp*. melitensis* | Fabaceae | CC | Endemic | Yes |  |
| *Ceratonia siliqua* | Fabaceae | R |  | Yes |  |
| ***Chamaerops humilis*** | Arecaceae | RR |  | Yes | Yes |
| ***Cupressus sempervirens*** | Cupressaceae | I |  | Yes |  |
| ***Cydonia oblonga*** | Rosaceae | RR |  | Yes |  |
| *Daucus lopadusanus* | Apiaceae | R | Subendemic | Yes | Yes |
| *Daucus rupestris* | Apiaceae | I | Subendemic | Yes | Yes |
| *Desmazeria pignatti* | Poaceae | I | Subendemic | Yes | Yes |
| ***Erica multiflora*** | Ericaceae | I |  | Yes |  |
| *Euphorbia dendroides* | Euphorbiaceae | RR |  | Yes |  |
| *Euphorbia melitensis* | Euphorbiaceae | C | Endemic | Yes | Yes |
| *Euphorbia paralias* | Euphorbiaceae | Not observed |  | Yes | Yes |
| *Euphorbia peplis* | Euphorbiaceae | Not observed |  | Yes | Yes |
| *Euphorbia terracina* | Euphorbiaceae | Not observed |  | Yes | Yes |
| *Ficus carica* | Moraceae | R |  | Yes |  |
| *Hyoseris frutescens* | Asteraceae | Not observed | Endemic | Yes | Yes |
| *Jacobaea maritima* subsp*. sicula* | Asteraceae | R | Subendemic |  |  |
| *Juncus acutus* | Juncaceae | RR |  | Yes |  |
| *Juncus maritimus* | Juncaceae | Not observed |  | Yes | Yes |
| ***Laurus nobilis*** | Lauraceae | RR |  | Yes |  |
| *Limonium melitense* | Plumbaginaceae | I | Endemic | Yes | Yes |
| *Limonium zeraphae* | Plumbaginaceae | RR | Endemic | Yes | Yes |
| *Linaria pseudolaxiflora* | Plantaginaceae | R | Subendemic | Yes | Yes |
| *Lotus halophilus* | Fabaceae | RR |  | Yes | Yes |
| ***Malus pumila*** | Rosaceae | RR |  | Yes |  |
| *Matthiola incana* subsp. *melitensis* | Brassicaceae | RR | Endemic | Yes | Yes |
| ***Morus nigra*** | Moraceae | RR |  | Yes |  |
| *Neotinea lactea* | Orchidaceae | Not observed |  | Yes |  |
| ***Olea europaea* var. *europaea*** | Oleaceae | R |  | Yes |  |
| ***Olea europaea* var. *silvestris*** | Oleaceae | R |  | Yes |  |
| ***Ophrys bertolonii*** | Orchidaceae | Not observed |  | Yes | Yes |
| ***Ophrys caesiella*** | Orchidaceae | RR |  | Yes | Yes |
| ***Ophrys iricolor* subsp*. mesaritica*** | Orchidaceae | RR |  | Yes | Yes |
| *Ophrys melitensis* | Orchidaceae | RR | Endemic | Yes | Yes |
| *Ophrys speculum* | Orchidaceae | RR |  | Yes | Yes |
| *Pancratium maritimum* | Amaryllidaceae | RR |  | Yes | Yes |
| *Phoenix dactylifera* | Arecaceae | Not observed |  | Yes |  |
| *Pinus halepensis* | Pinaceae | I |  | Yes |  |
| *Pistacia lentiscus* | Anacardiaceae | CC |  | Yes |  |
| *Plocama calabrica* | Rubiaceae | Not observed |  | Yes | Yes |
| *Polygonum maritimum* | Polygonaceae | RR |  | Yes | Yes |
| *Prunus domestica* | Rosaceae | Not observed |  | Yes |  |
| *Prunus dulcis* | Rosaceae | R |  | Yes |  |
| ***Prunus insititia*** | Rosaceae | RR |  | Yes |  |
| *Prunus persica* | Rosaceae | RR |  | Yes |  |
| ***Punica granatum*** | Lythraceae | R |  | Yes |  |
| ***Pyrus communis*** | Rosaceae | RR |  | Yes |  |
| *Rhamnus oleoides* | Rhamnaceae | RR |  | Yes |  |
| *Romulea variicolor* | Iridaceae | R | Subendemic | Yes |  |
| ***Ruppia maritima*** | Ruppiaceae | RR |  | Yes | Yes |
| *Salsola melitensis* | Amaranthaceae | I | Endemic | Yes | Yes |
| ***Schoenus nigricans*** | Cyperaceae | RR |  | Yes | Yes |
| *Scilla sicula* | Asparagaceae | Not observed | Subendemic | Yes | Yes |
| *Tamarix africana* | Tamaricaceae | R |  | Yes |  |
| ***Tetraclinis articulata*** | Cupressaceae | RR |  | Yes |  |
| *Valantia hispida* | Rubiaceae | Not observed |  | Yes | Yes |
| ***Verbascum creticum*** | Scrophulariaceae | Not observed |  | Yes | Yes |
| *Zannichellia melitensis* | Potamogetonaceae | RR | Endemic | Yes | Yes |

**Appendix E**

Alien species recorded or present on Comino.

Table D List of alien species recorded from Comino including their estimated occurrence classified as very rare (**RR**); rare (R); infrequent/scarce, (**I**) Frequent, (**C**); very common throughout (**CC**), or recorded in the past but not observed in this study (**nil**); and the level of invasiveness each species exhibits on the archipelago (Low, Mild, Moderate, Elevated, High)

| Species | Plant Family | New Record for Comino | Estimated frequency | Declared Invasive in Malta | Invasiveness on Comino |
| --- | --- | --- | --- | --- | --- |
| *Acacia saligna* s.l. | Fabaceae |  | I | Yes | Elevated |
| ***Aeonium arboreum*** | Crassulaceae | yes | RR |  | Low |
| *Agave americana* subsp*. americana* | Asparagaceae |  | R | Yes | Mild |
| ***Agave attenuata*** | Asparagaceae | yes | RR |  | Low |
| ***Agave sisalana*** | Asparagaceae | yes | RR | Yes | Low |
| *Ailanthus altissima* | Simaroubaceae |  | R | Yes | Elevated |
| *Allium cepa* | Amaryllidaceae |  | nil |  | Not observed |
| *Allium sativum* | Amaryllidaceae |  | nil |  | Not observed |
| ***Aloe ciliaris*** | Asphodelaceae | yes | RR |  | Low |
| *Aloe vera* | Asphodelaceae |  | nil |  | Not observed |
| ***Anredera cordifolia*** | Basellaceae | yes | RR |  | Low |
| ***Aptenia lancifolium*** | Aizoaceae | yes | RR | Yes | Low |
| *Arundo donax* | Poaceae |  | R | Yes | Mild |
| ***Bougainvillae spectabilis*** | Nyctaginaceae | yes | RR |  | Low |
| *Brassica oleracea* s.l. | Brassicaceae |  | nil |  | Not Observed |
| *Carpobrotus acinaciformis* | Aizoaceae |  | R | Yes | Moderate |
| *Carthamus tinctorius* | Asteraceae |  | nil |  | Not observed |
| ***Chasmanthe floribunda*** | Iridaceae | yes | RR | Yes | Low |
| ***Centaurea diluta*** | Asteraceae | yes | RR |  | Low |
| ***Citrus limon*** | Rutaceae | yes | RR |  | Low |
| *Coix lacryma-jobi* | Poaceae |  | nil |  | Not Observed |
| *Cuminum cyminum* | Apiaceae |  | nil |  | Not Observed |
| ***Cydonia oblonga*** | Rosaceae | yes | RR |  | Low |
| *Erigeron bonariensis* | Asteraceae |  | nil |  | Not Observed |
| ***Eriobotrya japonica*** | Rosaceae | yes | RR |  | Low |
| *Eruca vesicaria* | Fabaceae |  | nil |  | Not Observed |
| *Eucalyptus gomphocephala* | Myrtaceae |  | R |  | Mild |
| ***Euphorbia hypericifolia*** | Euphorbiaceae | yes | RR |  | Mild |
| *Hordeum vulgare* | Poaceae |  | nil |  | Not observed |
| ***Hylocereus undatus*** | Cactaceae | yes | RR |  | Low |
| ***Lantana camara*** | Verbenaceae | yes | RR |  | Low |
| ***Lavandula dentata*** | Lamiaceae | Yes | RR |  | Low |
| ***Malus pumila*** | Rosaceae | yes | RR |  | Low |
| ***Mirabilis jalapa*** | Nyctaginaceae | yes | RR |  | Low |
| ***Morus nigra*** | Moraceae | yes | RR |  | Low |
| ***Musa × paradisiaca*** | Musaceae | yes | RR |  | Low |
| *Nicotiana glauca* | Solanaceae |  | RR |  | Low |
| *Opuntia ficus-indica* | Cactaceae |  | R | Yes | Moderate |
| ***Opuntia microdasys*** | Cactaceae | yes | RR |  | Low |
| ***Opuntia vulgaris* s.l.** | Cactaceae | yes | RR |  | Mild |
| *Oxalis pes-caprae* | Oxalidaceae |  | I | Yes | High |
| ***Oxalis pes-caprae* var. *pleniflora*** | Oxalidaceae | yes | R | Yes | High |
| ***Pelargonium ×hybridum* (=*P. ×hortorum*)** | Geraniaceae | yes | RR |  | Low |
| ***Pelargonium cucullatum*** | Geraniaceae | yes | RR |  | Low |
| ***Phoenix canariensis*** | Arecaceae | yes | R |  | Low |
| ***Pittosporum tobira*** | Pittosporaceae | yes | R |  | Mild |
| *Prunus domestica* | Rosaceae |  | nil |  | Not observed |
| ***Prunus insititia*** | Rosaceae | yes | RR |  | Low |
| *Prunus persica* | Rosaceae |  | RR |  | Low |
| ***Pyrus communis*** | Rosaceae | yes | RR |  | Low |
| *Ricinus communis* | Euphorbiaceae |  | RR | Yes | Low |
| ***Sedum adolphii*** | Crassulaceae | yes | RR |  | Low |
| *Tamarix gallica* | Tamaricaceae |  | RR |  | Low |
| *Triticum aestivum* | Poaceae |  | nil |  | Not observed |
| *Triticum durum* | Poaceae |  | RR |  | Low |
| *Vicia ervilia* | Fabaceae |  | nil |  | Not Observed |
| *Vicia faba* | Fabaceae |  | RR |  | Low |
| *Vitis vinifera* | Vitaceae |  | RR |  | Low |
| ***Washingtonia filifera*** | Araceae | yes | RR |  | Low |
| ***Yucca gigantea*** | Asparagaceae | yes | RR |  | Low |
| ***Zantedeschia aethiopica*** | Araceae | yes | RR |  | Low |

**Appendix F**

New records of vascular plants for the Comino archipelago resulting from this study.

Table E: New records on Comino archipelago (79 species) observed during the 21 surveys conducted between 2019 and 2025, specifically on the dates 03/02/2019 [**survey # 1**], 30–31/Mar/2019 [**2**], 4-7/05/2019 [**3**], 31/08/2019 [**4**], 19/10/2019 [**5**], 04/06/2020 [**6a,b**], 11/10/2020 [**7**], 01/11/2020 [**8**], 20/03/2021 [**9**], 26/03/2021 [**10**], 11/04/2021 [**11**], 15/04/2021 [**12a-12c**], 25/04/2021 [**13**], 04/05/2021 [**14**], 21/11/2021 [**15**], 15/03/2022 [**16**], 19/04/2022 [**17**], 27/05/2022 [**18**], 01/05/2023 [**19**], 26/11/2024 [**20a-20c**], 04/04/2025 [**21**]. The table also includes the estimated frequencies using the following legend: [**RR**] very rare; [**R**] rare, [**I**] scarce, fragmented or frequent in a few places, [**C**] frequent–common, [**CC**] very common throughout Finally, the status of each species in the Maltese Islands Species denoted by **[X]** are based on personally communicated records and have not been directly observed during our surveys.

| No | Species | Plant Fa  mily | Visit when first recorded | Estimated frequency | Status  in the  Maltese Islands |
| --- | --- | --- | --- | --- | --- |
| 1 | *Aeonium arboreum* | Crassulaceae | 2 | RR | Alien |
| 2 | *Agave sisalana* | Asparagaceae | 6 | RR | Inv. Alien |
| 3 | *Agave attenuata* | Asparagaceae | 9 | RR | Alien |
| 4 | *Aloe ciliaris* | Asphodelaceae | 9 | RR | Alien |
| 5 | *Anredera cordifolia* | Basellaceae | 9 | RR | Alien |
| 6 | *Aptenia lancifolium* | Aizoaceae | 2 | RR | Inv. Alien |
| 7 | *Asphodelus fistulosus* | Asphodelaceae | 18 | RR | Native |
| 8 | *Atriplex halimus* | Amaranthaceae | 1 | RR | Native |
| 9 | *Atriplex prostrata* | Amaranthaceae | 8 | RR | Native |
| 10 | *Blackstonia acuminata* | Clusiaceae | 2 | I | Native |
| 11 | *Bougainvillaea spectabilis* | Nyctaginaceae | 2 | RR | Alien |
| 12 | *Brachypodium hybridum* | Poaceae | 2 | R | Native |
| 13 | *Brassica rapa* subsp. *sylvestris* | Brassicaceae | 2 | RR | Native |
| 14 | *Briza minor* | Poaceae | 3 | RR | Native |
| 15 | *Bromus alopecuros* | Poaceae | 17 | RR | Native |
| 16 | *Carthamus lanatus* | Asteraceae | 7 | RR | Native |
| 17 | *Chasmanthe floribunda* | Iridaceae | 18 | RR | Inv. Alien |
| 18 | *Catapodium pauciflorum* | Poaceae | 11 | R | Native |
| 19 | *Catapodium rigidum* subsp. *major* | Poaceae | 13 | RR | Native |
| 20 | *Centaurea diluta* | Asteraceae | 18 | RR | Alien |
| 21 | *Schenkia spicata* | Gentianaceae | 8 | RR | Native |
| 22 | *Centaurium tenuiflorum* | Gentianaceae | 3 | I | Native |
| 23 | *Chamaerops humilis* | Arecaceae | 9 | RR | Native |
| 24 | *Citrus limon* | Rutaceae | 13 | RR | Native |
| 25 | *Convolvulus siculus* | Convolvulaceae | 2 | RR | Native |
| 26 | *Cupressus sempervirens* | Cupressaceae | 1 | I | Native |
| 27 | *Cydonia oblonga* | Rosaceae | 3 | RR | Alien |
| 28 | *Daucus carota* | Apiaceae | 13 | RR | Native |
| 29 | *Daucus carota* subsp*. commutatus* var. *tenuisectus* | Apiaceae | 7 | R | Native |
| 30 | *Erica multiflora* | Ericaceae | 1 | I | Native |
| 31 | *Eriobotrya japonica* | Rosaceae | 2 | RR | Alien |
| 32 | *Erodium cicutarium* subsp. *salzmannii* | Geraniaceae | 3 | RR | Native |
| 33 | *Euphorbia hypericifolia* | Euphorbiaceae | 5 | RR | Alien |
| 34 | *Fumaria parviflora* | Papaveraceae | 13 | RR | Native |
| 35 | *Gladiolus communis* s.l. | Iridaceae | 17 | RR | Native |
| 36 | *Hedypnois cretica* | Asteraceae | 2 | RR | Native |
| 37 | *Hylocereus undatus* | Cactaceae | 11 | RR | Alien |
| 38 | *Lantana camara* | Verbenaceae | 2 | RR | Alien |
| 39 | *Laurus nobilis* | Lauraceae | 2 | RR | Native |
| 40 | *Lavandula dentata* | Lamiaceae | 15 | RR | Alien |
| 41 | *Lonicera implexa* | Caprifoliaceae | 1 | R | Native |
| 42 | *Lysimachia loeflingii* | Primulaceae | 1 | RR | Native |
| 43 | *Lythrum hyssopifolia* | Lythraceae | 17 | R | Native |
| 44 | *Malus pumila* | Rosaceae | 2 | RR | Alien |
| 45 | *Malva nicaeensis* | Malvaceae | 18 | RR | Native |
| 46 | *Mirabilis jalapa* | Nyctaginaceae | 15 | RR | Alien |
| 47 | *Morus nigra* | Moraceae | 18 | RR | Alien |
| 48 | *Musa × paradisiaca* | Musaceae | 15 | RR | Alien |
| 49 | *Olea europaea* var*. europaea* | Oleaceae | 1 | R | Arch |
| 50 | *Olea europaea* var. *silvestris* | Oleaceae | 3 | R | Native |
| 51 | *Ophrys bertolonii* | Orchidaceae | not observed | X | Native |
| 52 | *Opuntia microdasys* | Cactaceae | 11 | RR | Alien |
| 53 | *Opuntia vulgaris* s.l. | Cactaceae | 13 | RR | Alien |
| 54 | *Oxalis pes-caprae* var. *pleniflora* | Oxalidaceae | 1 | R | Inv. Alien |
| 55 | *Pelargonium cucullatum* | Geraniaceae | 2 | RR | Alien |
| 56 | *Pelargonium ×hortorum* | Geraniaceae | 1 | RR | Alien |
| 57 | *Phoenix canariensis* | Arecaceae | 1 | R | Alien |
| 58 | *Pittosporum tobira* | Pittosporaceae | 2 | R | Alien |
| 59 | *Polypogon monspeliensis* | Poaceae | 21 | RR | Native |
| 60 | *Polypogon subspathaceus* | Poaceae | 2 | C | Native |
| 61 | *Prunus insititia* | Rosaceae | 2 | RR | Alien |
| 62 | *Punica granatum* | Lythraceae | 1 | R | Arch |
| 63 | *Pyrus communis* | Rosaceae | 3 | RR | Alien |
| 64 | *Rosmarinus officinalis* | Lamiaceae | 2 | RR | Native |
| 65 | *Ruppia maritima* | Ruppiaceae | 18 | RR | Native |
| 66 | *Schoenus nigricans* | Cyperaceae | 10 | RR | Native |
| 67 | *Sedum adolphii* | Crassulaceae | 18 | RR | Alien |
| 68 | *Setaria adhaerens* | Poaceae | 2 | RR | Native |
| 69 | *Sinapis alba* | Brassicaceae | 1 | RR | Native |
| 70 | *Spergularia diandra* | Caryophyllaceae | 11 | RR | Native |
| 71 | *Tetraclinis articulata* | Cupressaceae | 2 | RR | Native |
| 72 | *Trifolium suffocatum* | Fabaceae | 9 | RR | Native |
| 73 | *Typha domingensis* | Typhaceae | 8 | RR | Native |
| 74 | *Umbilicus rupestris* | Crassulaceae | 1 | RR | Native |
| 75 | *Valeriana rubra (=Centranthus ruber)* | Caprifoliaceae | 17 | RR | Native |
| 76 | *Verbascum creticum* | Scrophulariaceae | not observed | X | Native |
| 77 | *Washingtonia filifera* | Araceae | 1 | RR | Alien |
| 78 | *Yucca gigantea* | Asparagaceae | 9 | RR | Alien |
| 79 | *Zantedeschia aethiopica* | Araceae | 3 | RR | Alien |

**Appendix G**

Records recorded in the past from Comino and not observed surveys reported in this study (referred to as lost records).

Table F: Species that have been historically recorded but have not been observed in the 21 surveys carried out between 2018 and 2025 Details include their original publication, status in the Maltese Islands, and a spot analysis of possible reasons for their absence. These reasons are categorised as: **Agriculture cessation** (species typically associated with agriculture that may decline if farming ceases); **Confused** (taxa that may have been misidentified or confused with closely related species); **Possibly extinct** (species considered genuinely recorded that no longer occur on the Comino archipelago) and **Inconclusive** (no plausible conclusion and may simply have not been detected in these surveys or casual occurrences).

| No | Species | | First record | | Status | | Rationale |
| --- | --- | --- | --- | --- | --- | --- | --- |
| Historical records (Zerapha, 1827 to Haslam et al 1977), hence not observed for 50 years or more | | | | | | | |
| 1 | *Adonis microcarpus* | | Sommier and Caruana Gatto (1915) | | Native | | Possibly extinct |
| 2 | *Aegilops ovata* | | Sommier and Caruana Gatto (1915) | | Native | | Confused (*A. geniculata*?) |
| 3 | *Allium cepa* | | Borg (1927) | | Alien | | Agriculture cessation |
| 4 | *Allium sativum* | | Borg (1927) | | Alien | | Agriculture cessation |
| 5 | *Allium subhirsutum* | | Borg (1927) | | Native | | Inconclusive |
| 6 | *Allium trifoliatum* | | Sommier and Caruana Gatto (1915) | | Native | | Inconclusive |
| 7 | *Aloe vera* | | Sommier and Caruana Gatto (1915) | | Alien | | Possibly extinct |
| 8 | *Ambrosia maritima* | | Haslam et al (1977) | | Native | | Possibly extinct |
| 9 | *Ammi majus* | | Sommier and Caruana Gatto (1915) | | Native | | Inconclusive |
| 10 | *Ammoides pusilla* | | Sommier and Caruana Gatto (1915) | | Native | | Inconclusive |
| 11 | *Anemone coronaria* | | Sommier and Caruana Gatto (1915) | | Native | | Possibly extinct |
| 12 | *Anthemis arvensis* | | Borg (1927) | | Native | | Inconclusive |
| 13 | *Anthemis tomentosa* | | Haslam et al (1977) | | Native | | Confused (*A. secundiramea*?) |
| 14 | *Anthoxanthum gracile* | | Borg (1927) | | Native | | Inconclusive |
| 15 | *Antirrhinum siculum* | | Borg (1927) | | Native | | Inconclusive |
| 16 | *Antirrhinum tortuosum* | | Borg (1927) | | Native | | Inconclusive |
| 17 | *Astragalus sesameus* | | Duthie (1874) | | Native | | Inconclusive |
| 18 | *Avena sterilis* | | Borg (1927) | | Native | | Inconclusive |
| 19 | *Bellis sylvestris* | | Sommier and Caruana Gatto (1915) | | Native | | Inconclusive |
| 20 | *Biscutella didyma* | | Borg (1927) | | Native | | Inconclusive |
| 21 | *Biscutella lyrata* | | Haslam et al (1977) | | Native | | Confused (*B. didyma*?) |
| 22 | *Blackstonia perfoliata* | | Duthie (1874) | | Native | | Confused (*B. acuminata*?) |
| 23 | *Brachypodium pinnatum* | | Borg (1927) | | Native | | Inconclusive |
| 24 | *Brassica oleracea* s.l. | | Borg (1927) | | Native | | Agriculture cessation |
| 25 | *Brassica rupestris* | | Haslam et al (1977) | | Native | | Inconclusive |
| 26 | *Briza maxima* | | Sommier and Caruana Gatto (1915) | | Native | | Inconclusive |
| 27 | *Bromus diandrus* | | Haslam et al (1977) | | Native | | Inconclusive |
| 28 | *Bromus hordeaceus* s.l. | | Sommier and Caruana Gatto (1915) | | Native | | Inconclusive |
| 29 | *Bromus tectorum* | | Haslam et al (1977) | | Native | | Confused (*B. rigidus*?) |
| 30 | *Bupleurum lancifolium* | | Sommier and Caruana Gatto (1915) | | Native | | Possibly extinct |
| 31 | *Calendula arvensis* | | Borg (1927) | | Native | | Inconclusive |
| 32 | *Calendula suffruticosa* | | Haslam et al (1977) | | Native | | Possibly extinct |
| 33 | *Capsella bursa-pastoris* | | Sommier and Caruana Gatto (1915) | | Native | | Inconclusive |
| 34 | *Carduus marmoratus* | | Sommier and Caruana Gatto (1915) | | Native | | Inconclusive |
| 35 | *Carduus pycnocephalus* subsp. *pycnocephalus* | | Sommier and Caruana Gatto (1915) | | Native | | Inconclusive |
| 36 | *Carex divisa* | | Duthie (1874) | | Native | | Possibly extinct |
| 37 | *Carthamus tinctorius* | | Borg (1927) | | Alien | | Agriculture cessation |
| 38 | *Catapodium hemipoa* | | Haslam et al (1977) | | Native | | Inconclusive / Confused with closely related *Catapodium* sp. |
| 39 | *Centaurea nicaeensis* | | Borg (1927) | | Native | | Inconclusive |
| 40 | *Cerastium glomeratum* | | Sommier and Caruana Gatto (1915) | | Native | | Inconclusive |
| 41 | *Clinopodium nepeta* | | Sommier and Caruana Gatto (1915) | | Native | | Inconclusive |
| 42 | *Convolvulus pentapetaloides* | | Sommier and Caruana Gatto (1915) | | Native | | Inconclusive |
| 43 | *Coix lacryma-jobi* | | Borg (1927) | | Alien | | Agriculture cessation |
| 44 | *Crassula vaillantii* | | Borg (1927) | | Native | | Inconclusive |
| 45 | *Cuminum cyminum* | | Borg (1927) | | Alien | | Agriculture cessation |
| 46 | *Cynoglossum creticum* | | Borg (1927) | | Native | | Inconclusive |
| 47 | *Dactylis glomerata* subsp. *hispanica* | | Borg (1927) | | Native | | Inconclusive |
| 48 | *Daucus gingidium* | | Sommier and Caruana Gatto (1915) | | Native | | Inconclusive / Confused |
| 49 | *Draba verna* | | Duthie (1875) | | Native | | Possibly extinct |
| 50 | *Erigeron bonariensis* | | Duthie (1874) | | Alien | | Possibly extinct |
| 51 | *Erodium moschatum* | | Sommier and Caruana Gatto (1915) | | Native | | Inconclusive |
| 52 | *Eruca vesicaria* | | Borg (1927) | | Alien | | Agriculture cessation |
| 53 | *Ervilia sativa* | | Borg (1927) | | Alien | | Agriculture cessation |
| 54 | *Eryngium maritimum* | | Sommier and Caruana Gatto (1915) | | Native | | Possibly extinct |
| 55 | *Euphorbia paralias* | | Sommier and Caruana Gatto (1915) | | Native | | Possibly extinct |
| 56 | *Euphorbia terracina* | | Sommier and Caruana Gatto (1915) | | Native | | Possibly extinct |
| 57 | *Fumana thymifolia* | | Borg (1927) | | Native | | Inconclusive |
| 58 | *Fumaria agraria* | | Sommier and Caruana Gatto (1915) | | Native | | Inconclusive |
| 59 | *Fumaria flabellata* | | Borg (1927) | | Native | | Inconclusive |
| 60 | *Fumaria officinalis* | | Sommier and Caruana Gatto (1915) | | Native | | Inconclusive |
| 61 | *Galium aparine* | | Borg (1927) | | Native | | Inconclusive |
| 62 | *Galium verrucosum* | | Sommier and Caruana Gatto (1915) | | Native | | Inconclusive |
| 63 | *Gastridium ventricosum* | | Sommier and Caruana Gatto (1915) | | Native | | Inconclusive |
| 64 | *Geranium molle* | | Sommier and Caruana Gatto (1915) | | Native | | Inconclusive |
| 65 | *Geranium robertianum* | | Borg (1927) | | Native | | Inconclusive |
| 66 | *Geranium rotundifolium* | | Borg (1927) | | Native | | Inconclusive |
| 67 | *Gladiolus italicus* | | Sommier and Caruana Gatto (1915) | | Native | | Inconclusive |
| 68 | *Hedera helix* | | Borg (1927) | | Native | | Possibly extinct |
| 69 | *Hordeum murinum* subsp. *leporinum* | | Sommier and Caruana Gatto (1915) | | Native | | Inconclusive |
| 70 | *Hordeum vulgare* | | Borg (1927) | | Alien | | Agriculture cessation |
| 71 | *Hyoseris frutescens* | | Borg (1927) | | Native | | Inconclusive |
| 72 | *Hyoseris radiata* | | Haslam et al (1977) | | Native | | Inconclusive |
| 73 | *Hypericum pubescens* | | Borg (1927) | | Native | | Inconclusive |
| 74 | *Juncus maritimus* | | Sommier and Caruana Gatto (1915) | | Native | | Possibly extinct |
| 75 | *Kickxia spuria* | | Borg (1927) | | Native | | Inconclusive |
| 76 | *Kundmannia sicula* | | Duthie (1875) | | Native | | Inconclusive |
| 77 | *Lamium amplexicaule* | | Borg (1927) | | Native | | Inconclusive |
| 78 | *Lathyrus articulatus* | | Borg (1927) | | Native | | Inconclusive |
| 79 | *Lathyrus clymenum* | | Sommier and Caruana Gatto (1915) | | Native | | Inconclusive |
| 80 | *Lathyrus ochrus* | | Borg (1927) | | Native | | Inconclusive |
| 81 | *Lolium temulentum* | | Borg (1927) | | Native | | Possibly extinct |
| 82 | *Medicago marina* | | Sommier and Caruana Gatto (1915) | | Native | | Inconclusive |
| 83 | *Medicago minima* | | Duthie (1874) | | Native | | Inconclusive |
| 84 | *Medicago orbicularis* | | Sommier and Caruana Gatto (1915) | | Native | | Inconclusive |
| 85 | *Medicago scutellata* | | Sommier and Caruana Gatto (1915) | | Native | | Inconclusive |
| 86 | *Medicago truncatula* | | Sommier and Caruana Gatto (1915) | | Native | | Inconclusive |
| 87 | *Melica ciliata* | | Borg (1927) | | Native | | Inconclusive |
| 88 | *Melilotus messanensis* | | Sommier and Caruana Gatto (1915) | | Native | | Inconclusive |
| 89 | *Melilotus sulcatus* | | Sommier and Caruana Gatto (1915) | | Native | | Inconclusive |
| 90 | *Mesembryanthemum crystallinum* | | Duthie (1874) | | Native | | Inconclusive |
| 91 | *Misopates orontium* | | Sommier and Caruana Gatto (1915) | | Native | | Inconclusive |
| 92 | *Neatostema apulum* | | Sommier and Caruana Gatto (1915) | | Native | | Possibly extinct |
| 93 | *Neotinea lactea* | | Sommier and Caruana Gatto (1915) | | Native | | Inconclusive |
| 94 | *Onopordum tauricum* | | Sommier and Caruana Gatto (1915) | | Native | | Last seen in the early 2000s by Timothy Tabone. Possibly extinct |
| 95 | *Orobanche picridis* | | Sommier and Caruana Gatto (1915) | | Native | | Inconclusive |
| 96 | *Phalaris paradoxa* | | Borg (1927) | | Native | | Inconclusive |
| 97 | *Phoenix dactylifera* | | Haslam et al (1977) | | Native | | Possibly extinct or confused with P canariensis |
| 98 | *Picris echioides* | | Sommier and Caruana Gatto (1915) | | Native | | Possibly extinct |
| 99 | *Piptatherum miliaceum* | | Borg (1927) | | Native | | Possibly extinct |
| 100 | *Plantago afra* | | Borg (1927) | | Native | | Inconclusive |
| 101 | *Plantago serraria* | | Sommier and Caruana Gatto (1915) | | Native | | Inconclusive |
| 102 | *Plocama calabrica* | | Haslam et al (1977) | | Native | | Possibly extinct |
| 103 | *Poa annua* | | Borg (1927) | | Native | | Inconclusive |
| 104 | *Poa bulbosa* | | Borg (1927) | | Native | | Possibly extinct |
| 105 | *Polycarpon alsinifolium* | | Duthie (1874) | | Native | | Confused (P tetraphyllum ?) |
| 106 | *Polygonum aviculare* | | Sommier and Caruana Gatto (1915) | | Native | | Inconclusive |
| 107 | *Portulaca oleracea* | | Borg (1927) | | doubtful ? | | Inconclusive |
| 108 | *Prunus domestica* | | Borg (1927) | | Alien | | Agriculture cessation |
| 109 | *Pseudorlaya pumila* | | Borg (1927) | | Native | | Possibly extinct |
| 110 | *Ranunculus saniculifolius* | | Borg (1927) | | Native | | Possibly extinct |
| 111 | *Ranunculus muricatus* | | Borg (1927) | | Native | | Possibly extinct |
| 112 | *Raphanus raphanistrum* | | Sommier and Caruana Gatto (1915) | | Native | | Possibly extinct |
| 113 | *Rapistrum rugosum* | | Borg (1927) | | Native | | Inconclusive |
| 114 | *Reseda lutea* | | Sommier and Caruana Gatto (1915) | | Native | | Possibly extinct |
| 115 | *Rhodalsine geniculata* | | Duthie (1875) | | Native | | Inconclusive |
| 116 | *Ridolfia segetum* | | Borg (1927) | | Native | | Possibly extinct |
| 117 | *Romulea columnae* | | Sommier and Caruana Gatto (1915) | | Native | | Inconclusive |
| 118 | *Rubus ulmifolius* | | Borg (1927) | | Native | | Possibly extinct |
| 119 | *Salsola soda* | | Sommier and Caruana Gatto (1915) | | Native | | Possibly extinct |
| 120 | *Salvia verbenaca* | | Sommier and Caruana Gatto (1915) | | Native | | Inconclusive |
| 121 | *Scabiosa atropurpurea* | | Sommier and Caruana Gatto (1915) | | Native | | Inconclusive |
| 122 | *Scandix pecten-veneris* | | Sommier and Caruana Gatto (1915) | | Native | | Inconclusive |
| 123 | *Scolymus grandiflorus* | | Sommier and Caruana Gatto (1915) | | Native | | Possibly extinct |
| 124 | *Scolymus hispanicus* | | Sommier and Caruana Gatto (1915) | | Native | | Possibly extinct |
| 125 | *Scolymus maculatus* | | Borg (1927) | | Native | | Possibly extinct |
| 126 | *Senecio vulgaris* | | Borg (1927) | | Native | | Inconclusive |
| 127 | *Setaria verticillata* | | Haslam et al (1977) | | Native | | Inconclusive |
| 128 | *Sherardia arvensis* | | Sommier and Caruana Gatto (1915) | | Native | | Inconclusive |
| 129 | *Silene colorata* | | Sommier and Caruana Gatto (1915) | | Native | | Inconclusive |
| 130 | *Silene nocturna* | | Borg (1927) | | Native | | Inconclusive |
| 131 | *Silene vulgaris* | | Sommier and Caruana Gatto (1915) | | Native | | Inconclusive |
| 132 | *Smyrnium olusatrum* | | Borg (1927) | | Native | | Inconclusive |
| 133 | *Spergularia rubra* | | Sommier and Caruana Gatto (1915) | | Native | | Inconclusive |
| 134 | *Stachys ocymastrum* | | Sommier and Caruana Gatto (1915) | | Native | | Possibly extinct |
| 135 | *Stellaria media* | | Haslam et al (1977) | | Native | | Inconclusive |
| 136 | *Stellaria neglecta* | | Borg (1927) | | Native | | Inconclusive |
| 137 | *Stellaria pallida* | | Borg (1927) | | Native | | Inconclusive |
| 138 | *Stipa capensis* | | Duthie (1874) | | Native | | Inconclusive |
| 139 | *Suaeda vera* | | Sommier and Caruana Gatto (1915) | | Native | | Possibly extinct |
| 140 | *Tetragonolobus purpureus* | | Sommier and Caruana Gatto (1915) | | Native | | Inconclusive |
| 141 | *Thesium humile* | | Borg (1927) | | Native | | Inconclusive |
| 142 | *Trifolium repens* | | Sommier and Caruana Gatto (1915) | | Native | | Inconclusive |
| 143 | *Trifolium resupinatum* | | Duthie (1874) | | Native | | Inconclusive |
| 144 | *Trifolium stellatum* | | Duthie (1874) | | Native | | Inconclusive |
| 145 | *Trifolium tomentosum* | | Sommier and Caruana Gatto (1915) | | Native | | Inconclusive |
| 146 | *Trisetaria aurea* | | Borg (1927) | | Native | | Inconclusive |
| 147 | *Triticum aestivum* | | Borg (1927) | | Alien | | Agriculture cessation |
| 148 | *Urtica pilulifera* | | Borg (1927) | | Native | | Possibly extinct |
| 149 | *Valantia hispida* | | Duthie (1874) | | Native | | Possibly extinct or confused with V muralis var. hispida |
| 150 | *Valeriana graciliflora* | | Sommier and Caruana Gatto (1915) | | Native | | Inconclusive |
| 151 | *Verbena officinalis* | | Sommier and Caruana Gatto (1915) | | Native | | Inconclusive |
| 152 | *Vicia faba* | | Borg (1927) | | Alien | | Agriculture cessation |
| 153 | *Vicia sativa* subsp. *nigra* | | Borg (1927) | | Native | | Inconclusive |
| 154 | *Vicia sativa* subsp. *sativa* | | Sommier and Caruana Gatto (1915) | | Native | | Inconclusive |
| 155 | *Vulpia fasciculata* | | Sommier and Caruana Gatto (1915) | | Native | | Inconclusive |
| Records reported in the last 30 years (since Mallia and Schembri 1991) and not currently observed on Comino | | | | | | | |
|  | | | | | | | |
| 156 | *Anacamptis pyramidalis* subsp. *urvilleana* | Stevens 2000 | | Native | | Last observed by one of us (SM) in Apr 2006 | |
| 157 | *Anacamptis collina* | Mallia and Schembri 1991 | | Native | |  | |
| 158 | *Euphorbia peplis* | Borg (1927) | | Native | | Last observed by one of us (SM) in June 2012 | |
| 159 | *Linum bienne* | Lanfranco (2004) | | Native | |  | |
| 160 | *Malva arborea* | Sciberras and Sciberras (2010) from Ħaġra ż-Żgħira | | Native | |  | |
| 161 | *Ophrys bertolonii* | Matthew Borg Cardona (Pers. Comm. 2019) | | Native | | Last observed by M Borg Cardona in 2019 | |
| 162 | *Polycarpon tetraphyllum* subsp. *diphyllum* | Lanfranco (2004) | | Native | |  | |
| 163 | *Scilla sicula* | Lanfranco (2004) | | Native | |  | |
| 164 | *Verbascum creticum* | Jeffrey Sciberras (Pers. Comm. 2021) | | Native | | Last observed by Jeffrey Sciberras in 2006 | |

**Appendix H**

Remarks and interesting additional notes on some species recorded from Comino archipelago.

1. *Acacia saligna.* This species was introduced to Comino primarily to support hunting activities, resulting in the establishment of three main populations: along the sides of Wied l‑Aħmar; an area southeast of the coastal tower; and another sizeable copse in the central/north‑western part of Comino. The records may include *Acacia pycnantha,* characterised by smaller leaves, and *A.* *cyanophylla*, which bears longer, more slender leaves; both of which are frequently confused with, or treated as synonyms or a complex of *A.* *saligna*. Recent removal efforts by the central authorities, following the designation of *A.* *saligna* as an invasive alien species of EU concern, led to the destruction of approximately 80% of the Comino population between 2021 and 2023. Nevertheless, evidence of regeneration and the presence of young saplings were recorded in 2024.
2. *Adiantum capillus-veneris.* This fern is confined to shaded caves and old wells, and hence it is quite rare on the archipelago. It is the only native fern to be found on Comino.
3. *Adonis microcarpa.* First recorded by Sommier and Caruana Gatto (1915) and confirmed as a variety of *A. annuus* by Borg (1927), *A. microcarpus* was reported as a frequent annual in Comino, typically found in irrigated or fallow fields, along footpaths, and field margins. Factors such as the abandonment of agriculture, prolonged drought, and overharvesting are likely to have led to its local extinction on Comino, paralleling its rarity over Malta and Gozo.
4. *Aegilops geniculata.* This annual grass is relatively widespread in Malta but has a limited presence on Comino, where it appears sporadically as isolated plants. Like many other annual species, it faces challenges to survival due to drought and shallow, non-clayey soils.
5. *Aeonium arboreum.* Large plants at the old hospital are likely neglected ornamental species that have become wild, forming sizable specimens. They remain locally restricted and do not spread to natural habitats.
6. *Ajuga iva.* Both purple and yellow flower forms were observed, often treated distinct taxa as *A. iva* var. *iva* and *A. iva* var. *pseudoiva,* respectively. However, they are considered conspecific according to global classifications such as WFO (2025) and POWO (2025).
7. *Allium lojaconoi.* It was described in 1982 as endemic to the Maltese Islands, although earlier Maltese floristic works had recorded it under the misapplied name *A.* *parciflorum* Viv. (Sommier & Caruana Gatto 1915; Borg 1927; Haslam et al. 1977). The species prefers shallow, sparsely vegetated rocky coastlines, and it is not rare on Comino, which offers extensive stretches of suitable habitat along its low, rocky shores.
8. *Allium polyanthum.* This wild leek was formally incorporated into the Maltese flora by Mifsud and Mifsud (2018), who demonstrated that for more than a century it had been consistently misidentified as *Allium melitense* (or *A. ampeloprasum* var. *melitense* Sommier & Caruana Gatto), *A. ampeloprasum* L., or *A. commutatum* Guss. On Comino, it was cited as *A. ampeloprasum* s.l. (Sommier & Caruana Gatto 1915; Haslam et al. 1977) and as *A. commutatum* (Borg 1927), both of which likely refer to *A. polyanthum*. The species is rare on Comino, occurring only as isolated individuals, whereas it is common and widespread on the main islands, particularly in steppe, valley banks, and degraded garrigue; habitats that are also well represented on Comino.
9. *Anacamptis pyramidalis* subsp. *urvilleana*. Unlike subsp. *pyramidalis*, this subspecies is very rare on Comino, and the first records are relatively recent (Stevens 2000). One of us saw stunted plants at the eastern cliffs of Comino (Stephen Mifsud, pers. obs. 23 Mar 2007) and has not seen them elsewhere in subsequent visits. No records have been reported on iNaturalist (2025).
10. *Arthemis urvilleana*. This endemic species is common on shallow rocky shores of the Maltese Islands but rare in the Comino archipelago, where it mainly forms dense mats on il‑Ħaġra il‑Kbira ta’ bejn il‑Kmiemen and, to a lesser extent, on parts of Cominotto. Its scattered and severely fragmented distribution may be due to rabbit grazing.
11. *Arundo donax*. Restricted to small pockets along Wied l‑Aħmar, the upper reaches of Wied Skalanova, and other pockets of semidry or artificial wetlands. It is considered an aggressive, invasive species and is being eradicated by Maltese authorities from many valleys. However, on Comino, the problem is less pronounced due to water scarcity and the absence of clayey soil.
12. *Asphodelus fistulosus*. Joe Attard reported this species to one of us [SM] from a recent afforested area. The plants manifested in three dense clumps near irrigation lines, with several scattered younger individuals, possibly offspring of the larger (introduced) specimens. Its introduction was later confirmed to have occurred around 2019–2020 (Ben Camilleri, pers. comm., Nov 2025). Given that the species has now undergone several generations of naturalisation, it is reasonable to consider it part of the flora of Comino.
13. *Asteriscus aquaticus*. This species represents the most common therophyte (annual) in the Comino archipelago, whereas it is scarce to rare in Malta and Gozo. It is encountered ubiquitously on exposed ground, displaying considerable morphological variation, where individuals may be reduced to only a few centimetres in shallow, nutrient‑poor soils, while in deeper loamy substrates they can develop into sizeable bushes.
14. *Bellis annua*. A single widespread population was found near the hospital and the storehouses to its west. It may have originated from a single accidental introduction, such as agricultural contamination of trough livestock animals from the mainland, and has become naturalised over time.
15. *Blackstonia perfoliata.* This species was first recorded as *Chlora perfoliata* (L.) L. (Duthie 1874), and Sommier and Caruana Gatto (1915) remarked it to be remarkably variable. Later, the var. *serotina* (W.D.J. Koch ex Rchb.) Bolzon was reported from arid ground (Borg 1927). Haslam et al. (1977), who based most of their taxonomy on Borg (op. cit.), updated Borg’s taxon to the genus *Blackstonia* and assigned all Maltese plants to *B.* *perfoliata* var. *serotina*. This variety occurs in the southern part of Europe, whereas *B.* *perfoliata* s. str. (L.) Huds. has a northern distribution. The variety *serotina* was then combined under *Blackstonia acuminata* (W.D.J. Koch & Ziz) Domin; however, in all subsequent Maltese floristic literature, the species was reported as *B.* *perfoliata* instead of *B.* *acuminata*. During the first preliminary visit in 2008, we studied several *Blackstonia* samples from Comino, and later from mainland Malta, which exhibited flowers measuring 6–9 mm across, narrowly lanceolate calyx lobes, and a distinct constriction at the base of the leaves, all characters of *B.* *acuminata*. Thus, the species occurring on Comino, and most likely the rest of the Maltese Islands, must be attributed to *B.* *acuminata*, not *B.* *perfoliata*.
16. *Brachypodium retusum.* A significant population was recorded on the north side of Cominotto, but then, it is almost absent from mainland Comino, except for two small patches, one at the eastern cliffs and one about 200m above (north of) Blue Lagoon.
17. *Carlina involucrata.* Historical Maltese literature treated *C. corymbosa* as comprising two varieties: var. *involucrata* Poir. and var. *globosa* Arc. (Borg 1927). However, current evidence and field observations indicate that only a single species is present in the Maltese Islands, and the earlier records by Sommier and Caruana Gatto (1915) and Borg (1927) most likely all correspond to *C. involucrata* s.str.
18. *Catapodium zwierleini.* It was originally recorded by Haslam et al. (1977), but under the misapplied name *C. hemipoa*. The species is very closely related to the widespread *C. rigidum*, and some individuals in Malta, and also on Comino, may correspond to *C. zwierleini*. However, the morphological separation between these taxa relies on subtle differences in spikelet size and their spacing on the rachis. Evidence from Malta indicates a continuum of intermediate forms, suggesting that *C. rigidum* may exhibit considerable phenotypic plasticity in habit, stature, and spikelet morphology in response to soil depth and aridity. Nonetheless, the possible presence of *C. zwierleini* cannot be excluded without a comprehensive, targeted taxonomic investigation.
19. *Centaurea melitensis.* Comino represents one of the few extant localities for *C. melitensis* within the Maltese Islands, and it also supports the largest known population in the country. Ongoing attempts to afforest Comino with Aleppo pine through poorly planned tree-planting initiatives are expected to negatively affect this population, primarily by increasing shading and accumulating leaf litter beneath the developing canopy.
20. *Centaurium pulchellum* and *C. tenuiflorum.* These two species are morphologically separated by the amount of branching, size and number of internodes along the main stem, and dimensions of the flower tube *C. pulchellum* is a smaller plant with few internodes and unpronounced branching. Sommier and Caruana Gatto (1915) have already noted the difficulty of identifying plants in Malta based on these size-related characters, because stunted plants of C. tenuiflorum in shallow, nutrient-deprived soil, as on Comino, can resemble *C. pulchellum.* Although our surveys indicate that we recorded both species, further genetic studies are needed to confirm whether there is a single variable species or both. Until such confirmations are ascertained, the two species are currently recorded from Comino.
21. *Ceratonia siliqua.* A significant population of mature trees is situated along the valley bank of Wied l-Aħmar, creating a small area of natural maquis alongside several olive and almond trees. One specimen is notably ancient, evidenced by its extensive canopy and multiple robust ascending branches. Additional large specimens are found in fields near the bay of San Niklaw and may constitute some of the oldest carob tree cultivations on Comino. Other smaller trees dispersed across the steppe and abandoned fields are likely either remnants of previously cultivated carob trees or have become naturalised in these environments.
22. *Chamaerops humilis.* One mature tree was found at the back of the hospital, and it appears to be of an old cultivation due to its large size.
23. *Chiliadenus bocconei.* Frequent in mainland Malta and Gozo but rare on Comino, with many plants observed on the eastern part of Comino.
24. *Convolvulus doneatus.* Borg (1927) mentions this taxon as a common perennial bindweed with elongated silvery leaves, however, it does not correspond to any described taxon (e.g. WFO, Euro+Med, POWO, etc.). This may be a misprint or an error by the author, possibly intended to refer to *C. lineatus*, which shares the characteristics described, is common in the Maltese Islands, and whose orthography of the epithet “*doneatus”* closely resembles *lineatus.*
25. *Convolvulus* spp. All species recorded are rare in the Comino archipelago, unlike in Malta and Gozo, where they are widespread and often form large, established populations. Encounters of *C. althaeoides* s.l. (including *C. althaeoides* subsp. *tenuissimus*) and *C. lineatus* were restricted to very small plants. It is assumed that these species prefer loamy and clayey soils, which are scarce on Comino. More established is *C. oleifolius* with a sizeable and widespread population located at Bejn il-Kmiemem area, including Cominotto and the islets.
26. *Crucianella maritima.* Similar to *Anthemis secundiramea*, this coastal species, frequently found in the Maltese Islands, is restricted to the islet of Cominotto. It was recorded as *C. rupestris,* but modern classifications put this in synonymy with *C. maritima* (WFO, POWO).
27. *Daucus rupestris / D. gingidium* complex. Dense populations of *Daucus cf.* *rupestris*, with some individuals showing affinities to *D.* *gingidium*, occur along several coastal stretches, particularly at Wied Scalanova, l‑Għar ta’ Bla Saqaf, the eastern shore of Cominotto, and the islets of Ħaġra Taħt il‑Mazz and Ħaġra l‑Kbira bejn il‑Kmiemen. In Malta, coastal wild carrots are typically frequent in coastal valleys and at valley mouths. The area of Comino known as bejn il‑Kmiemen is thought to represent a submerged rocky valley, a hypothesis further supported by the presence of this wild carrot assemblage. This complex of closely related and poorly understood taxa requires a fresh study, not only in Comino but also in the entire Maltese Islands. Studying the whole community of the *Daucus carota* complex on Comino was beyond the scope of this study, but a distinct population within this *D.* *rupestris* complex was identified by one of us [SM] during some visits, and when studied thoroughly, it was found to correspond to a new taxon for Comino and the Maltese Islands (see below).
28. *Daucus carota* subsp. *commutatus* var. *tenuisectus* (Degen ex Palyi) Reduron.
    During one visit to Cominotto (11 October 2020), populations of coastal wild carrots appeared different from *D.* *rupestris* s.l., by being smaller plants with leaves arching downward, leaflets somewhat more open and branching perpendicularly, narrow segments, and achenes with short spines. A detailed morphological analysis was taken and consulted with a *Daucus* expert, Jean‑Pierre Reduron. He confirmed the species as *Daucus carota* subsp. *commutatus* (Paol.) Thell. var. *tenuisectus* (Degen ex Palyi) Reduron, described in Reduron (2007)* (Jean‑Pierre Reduron, pers. comm., 5 Nov 2020). A comprehensive study detailing this discovery is currently in preparation and is expected to be published in 2026.

* Reduron, J‑P. (2007). *Ombellifères de France* Vol. 2: monographie des Ombellifères (Apiaceae) et plantes alliées, indigènes, naturalisées, subspontanées, adventices ou cultivées de la flore française. *Bulletin de la Société Botanique du Centre‑Ouest*, nouv. sér., num. spéc. 27: 565–1142 (578 pp).

1. *Desmazeria sicula / D. pignatti.* *Brullo* was described as a smaller plant with diminutive flowering parts than its very close relative *D.* *sicula*, endemic to southern Sicily, the Pelagian Islands and Malta (Brullo and Pavone 1985)*. However, several authors and classifications (WFO, POWO, GBIF) do not consider it distinct and therefore treat it as conspecific with *D.* *sicula*. This small grass is common in arid coastal areas of the Maltese Islands, often growing in a thin film of soil in rock basins, as similarly found on Comino and Cominotto (e.g., at the Għemmieri peninsula). It was recorded in historic literature as *Catapodium siculum* (Jacq.) Link (Duthie 1874).

* Brullo, S. & Pavone, P. (1985). Taxonomic considerations on the genus Desmazeria (Gramineae) with description of a new species: Desmazeria pignattii. Willdenowia 15: 99–106.

1. *Erica multiflora.* Despite being found on Comino in several sizeable populations (e.g., a large population 300 m south of the chapel), it has not been recorded in the previously cited literature. Its omission is rather unexplainable, and it does not seem to be a recent introduction.
2. *Erodium malacoides.* Mostly found as extremely stunted and small plants with few leaves, formed by plants that were not more than 12 to 15 cm across. This is another good example in which poor, shallow soils generate ecotypes that are much smaller and less prolific than typical forms.
3. *Erodium salzmannii.* Compared to *E.* *cicutarium* (L.) L’Hér., *E.* *salzmannii* is more xerophytic, adapted to rocky Mediterranean habitats, and less common in anthropogenic environments such as fields or footpaths. A few specimens with a woody stem at the base and poorly segmented leaves were observed on arid ground on Comino and were attributed to E. salzmannii; however, further study may be required to confirm this identification.
4. *Filago pyramidata.* We are reporting this group of closely related taxa as *F.* *pyramidata* s.l., knowing that some authors split this complex into several distinct species such as *F.* *cossyrensis*, *F.* *gussonei*, *F.* *lojaconoi*, *F.* *prostrata*, etc.
5. *Frankenia* spp. Comino, and most probably the entire Maltese archipelago, harbours two species of *Frankenia* which are well distinct when observed carefully. *Frankenia pulverulenta*, cited in many local works, is easily distinguished by its flattened leaves and glabrous to subglabrous bracts. The other species, with linear (= deeply folded) leaves and hirsute bracts, is attributed to *F.* *hirsuta*. Some authors state that *F.* *laevis* / *F.* *pulverulenta* and *F.* *hirsuta* co‑occur in intermixed populations (Borg 1927; Haslam et al. 1977), but at least the plants on Comino corresponded only to isolated and distinct populations, albeit sometimes in close vicinity. In addition, *F.* *pulverulenta* is by far more abundant than *F.* *hirsuta* on Comino, unlike in Gozo and mainland Malta.
6. *Gladiolus communis* s.l. In Comino, *G.* *segetum* (= *G.* *italicus*) has been continuously recorded in the past (Sommier and Caruana Gatto 1915; Borg 1915; Haslam et al. 1977), with some authors stating that it is frequent in or near cultivated fields (Haslam et al. 1977). Current surveys show that the species is now very rare, and the few individuals checked were all *G.* *communis* s.l. In the past, *G.* *segetum*, *G.* *dubius* and *G.* *byzantinus* have been confused, and by the beginning of the 2000s only *G.* *italicus* was being reported from the Maltese Islands, with the other two species considered doubtful occurrences or very rare. However, a preliminary study revealed that most *Gladiolus* populations in Malta corresponded to *G.* *communis* s.l., and only a few populations were actually *G.* *italicus* (Mifsud and Hamilton 2013). Thus, although *G.* *italicus* was reported from Comino, it is probable that it should refer to *G.* *communis*, in line with findings in Malta and Gozo.
7. *Hyoseris lucida.* This species, first reported from Malta and Comino by Borg (1927) may refer to the endemic species *H. frutescens*, as both plants resemble each other in both being succulent, shiny-leaved perennials. However, Borg (1927) reports *H. lucida* as common on Malta and Comino, whereas *H. frutescens* is restricted to Gozo (and very rare in northern Malta). It has never been recorded from Comino, including our surveys. The precise identity of *H. lucida* sensu Borg thus remains unclear.
8. *Juncus hybridus / J. bufonius.* Several old floras record *J. bufonius* s.l. from the Maltese Islands, including the closely related *J.* *sorrentinoi* Parl. (= *J.* *bufonius* var. *sorrentinii* (Parl.) Husn.) (Sommier and Caruana Gatto 1915; Borg 1927; Haslam et al. 1977). All specimens of this aggregate examined from Comino proved to be *J.* *hybridus*. A large population was found in the marsh behind Santa Marija Bay, but was destroyed by attempts to recreate the marsh behind the bay using heavy machinery and bulldozers.
9. *Linaria pseudolaxiflora*. This species was known from two locations in Comino (Mifsud 2013*), but these surveys confirmed it from at least six locations, including one in Cominotto. One population is negatively affected by recent afforestation with *Pinus halepensis*.

* Mifsud, S. (2013). Distribution of some rare or endemic chasmophytic and rupestral species growing along the coastal cliffs of the Maltese Islands. *Webbia* 68. 10.1080/00837792.2013.807451.

1. *Lygeum spartium.* The single population recorded from the Comino archipelago was from il‑Ħaġra ż‑żgħira ta’ bejn il‑Kmiemen. It was not observed on Comino and Cominotto.
2. *Lysimachia* (=*Anagallis*) spp. Despite Comino's arid conditions, *Lysimachia arvensis* and *L.* *loeflingii* are common and form a mixed population with flowers of varying colours. Comino provides an environment suitable for observing these related species and investigating potential hybridisation.
3. *Malva setigera (= Althaea hirsuta).* One of the few species that can be encountered on Comino, but is probably extirpated or extremely rare in Malta and Gozo. It occurs as sporadic small individuals and is never observed as a population or in small clumps. Two plants geomarked in March 2021 were subsequently found in April to have been dug up or partially consumed, presumably by rabbits. Notably, other *Malva* species that are widespread in Malta, such as *M. cretica* (L.), Pau, *M.* *sylvestris* L., and *M.* *parviflora* Sm., are likewise rare on Comino.
4. *Matthiola incana subsp. melitensis***.** The record of this plant from il‑Ħaġra ta’ Taħt il‑Mazz, first reported by Sciberras and Sciberras (2010), is corroborated by the present study. Its apparent restriction to this remote islet is intriguing. A plausible explanation is that the species may once have been more widespread around the Kmiemen area but was subjected to uncontrolled harvesting in the more accessible parts of Comino due to its attractive flowers and rarity, leaving the islet of Taħt il‑Mazz and nearby sea boulders (which are very difficult to reach and largely undisturbed) as its final refugium on the Comino archipelago.
5. *Medicago* spp*.* Of the seven *Medicago* species recorded in the past, only two still occur on Comino – *M.* *polymorpha* and *M.* *littoralis*, both rare. In contrast, they are very common on Malta and Gozo. A sizeable population of *M.* *polymorpha* was found in fenced fields near Art Hażina, which are still managed for crop production by one of the farmers living on Comino. Like *Sonchus*, *Malva*, *Foeniculum*, and other common species in mainland Malta but rare or absent on Comino, *Medicago* is assumed to have become extirpated or severely declined due to long-term rabbit grazing.
6. *Mesembryanthemum crystallinum.* This was recorded only by Duthie (1874–75) from Comino and Cominotto and later cited by Haslam et al. (1977). The absence of records by Sommier and Caruana Gatto (1915) and Borg (1927) suggests Duthie (op. cit.) may have confused *M.* *nodiflorum* (not recorded in his work) with *M.* *crystallinum*.
7. *Narcissus tazetta* s.l. One of the most common plants on Comino, particularly abundant along the eastern rocky slopes, where a dense population extending for over 1 km likely represents the largest continuous stand within the Maltese archipelago. It is also frequent in abandoned fields and steppe, indicating that farmers valued this charismatic species and deliberately introduced it into their fields. Later, when agriculture was abandoned, this wild daffodil subsequently spread and became well established in these neglected field plots and steppe.
8. *Opuntia* spp*.* Present in limited numbers and virtually absent from the eastern half of Comino and the surrounding satellite islands and islets. The scarcity of prickly pears, together with the notable rarity of other invasive alien species such as *Oxalis*, *Ricinus*, and *Arundo*, as well as many common weeds and ruderal plants that are widespread in Malta and Gozo, contributes significantly to the pristine character of Comino’s landscape and the general impression that the island’s vegetation is distinct from that of Malta and Gozo.
9. *Orobanche* spp**.** Another genus that is generally rare on Comino, with the exception of *O.* *ramosa* s.l., which is occasionally met parasitising *Oxalis pes‑caprae*. This scarcity is most likely due to the absence of suitable host species such as *Galactites*, *Sonchus*, *Glebionis*, and various Fabaceae members, which are common on mainland Malta and serve as primary hosts for many broomrapes. Another species observed in our surveys is *O.* *balsensis* (J.A. Guim.) Carlón, M. Laínz, Moreno Mor. & Ó. Sánchez, and represents another new record for the Maltese Islands. A comprehensive study of this taxon is currently in preparation (unpublished study on Orobanchaceae in Malta by Stephen Mifsud). *Orobanche cernua* is rare, as in the rest of the Maltese Islands, and was found growing on *Limbarda crithmoides* (L.) Dumort. Historical records of *O.* *picridis* F. Schulz and *O.* *minor* Sm. from the archipelago most likely correspond to *O.* *balsensis* due to their close resemblance.
10. *Oxalis pes-caprae*. One ecological relief in the vegetation of the Comino archipelago is the scarcity of the invasive species *Oxalis pes-caprae*. Its presence is confined to abandoned fields and steppe habitats, with no further naturalisation into rocky habitats. However, a significant incursion was observed in the Santa Marija sand dune, where the species occurred in large numbers. On Cominotto, its distribution is highly restricted, and it is absent from the other islets. The limited spread of *O. pes-caprae* may be attributed to the predominance of compact, shallow, clay-poor soils and, undoubtedly, the low level of anthropogenic disturbance across the archipelago.
11. *Phoenix dactylifera.* Haslam et al. (1977) recorded this species, but our surveys found only *P.* *canariensis*. It remains unclear whether Haslam et al. (1977) misidentified *P.* *canariensis* for *P.* *dactylifera*, or if *P.* *dactylifera* once existed and perished, for example, due to palm‑weevil infestations that have affected date palms in Malta (Mizzi et al. 2009).
12. *Phragmites australis.* This species was once widespread at the mouth of Wied l-Aħmar valley near the Santa Marija dune. However, tourism development and agricultural changes led to the destruction of this population. Fortunately, some of these plants have been introduced at the perimeter of a water catchment area at Art Ħażina, which is currently the only population surviving on Comino.
13. *Pistacia lentiscus.* The most common shrub on Comino Island (less so on Cominotto and the islets), where it is found all over the island except at the coast. Curiously, it was not mentioned in the historical floristic literature, with the first record for the archipelago provided only by Sciberras and Sciberras (2010) from Cominotto and Taħt il‑Mazz. Its omission from earlier inventories may reflect an assumption that such a conspicuous and widespread species had already been documented by others earlier, rather than an actual absence in the past. Nevertheless, one would expect that Duthie (1874, 1875a, 1875b) or Sommier and Caruana Gatto (1915) would include this species in the checklist of vascular plants from Comino. The absence of records of *P.* *lentiscus* by these authors remains obscure.
14. *Plantago* spp. The species of *Plantago* occurring in Malta requires renewed taxonomic investigation. According to classical checklists and records, Comino hosts several forms of the variable *P. coronopus*, *P. serraria*, *P. afra*, and the rare *P. weldenii*, typically restricted to footpaths and unpaved tracks. The conversion of these old footpaths into asphalted roads would pose a significant threat to such sensitive taxa, as well as to other species adapted to these microhabitats (e.g. *Catapodium* spp., *Filago* spp., *Vulpia* spp., *Romulea* spp., *Galium murale,* etc.)
15. *Plocama*  (=*Putoria*) *calabrica*. This rare, red-listed and legally protected species was first recorded from Comino by Haslam et al. (1977). Subsequent observations confirmed its continued presence through the 1990s and 2000s, with Edwin Lanfranco (pers. comm., 2020) reporting sightings in the vicinity of the chapel and the tennis courts near the old hotel. Its current confirmation from Comino would have been welcome; however, many attempts to locate it during these surveys were unsuccessful, and it may now be extirpated from Comino.
16. Poaceae. On Comino, grasses are rare, and their diversity is limited to 32 occurring species (a total of 58 species recorded in the past). In comparison, over 120 species occur in mainland Malta and Gozo, represented by several genera, mostly of annual grasses, namely *Aira*, *Avena*, *Bromus*, *Hordeum*, *Lagurus*, *Lolium*, *Poa*, *Parapholis*, *Phalaris*, *Oryzopsis* (= *Piptatherum*), *Trisetaria*, *Vulpia*, and others. The most common grass species on Comino are restricted to *Catapodium* spp. near the coast and *Polypogon maritimus* (and the closely related *P.* *subspathaceus*) throughout the archipelago. Other species that are neither common nor rare include *Bromus fasciculatus*, *B.* *madritensis*, *Lagurus ovatus* and *Vulpia* spp. The remaining species were found to be rare on Comino. The exact reasons for this difference remain unclear, but may include rabbits selectively grazing on most grasses while avoiding *Polypogon* species, as well as low soil clay content that limits water retention and makes survival difficult for some annual grasses. The absence (or almost) of grasses commonly found in Malta, such as *Avena* spp., *Cynodon dactylon*, *Dactylis* spp., *Hordeum murinum* s.l., *Lolium* spp., *Oryzopsis* spp., *Phalaris* spp., *Poa annua*, and *Stipa capensis*, constitutes another distinctive vegetative feature that differentiates Comino from the larger islands of Malta.
17. *Romulea variicolor.* The commonly occurring Maltese Sand Crocus, recorded as *R.* *ramiflora*, was reclassified as a new subendemic species, *R. variicolor* (Mifsud 2015); hence, past records of *R. ramiflora* on Comino similarly correspond to *R. variicolor*, although its presence is rare and sporadic. *R.* *columnae* was also previously recorded from Comino, but it was not observed during our surveys. The rare occurrence of *Romulea* spp. is another distinct difference between Comino's vegetation and that of the larger islands.
18. *Ruppia maritima.* This species was first recorded on Comino in May 2022 from an artificial water pond located in the area known as Art Hażina. The means of introduction remain unclear, with possibilities including natural avian dispersal or human introduction. Subsequently, the species was introduced into an artificial brackish pond behind the Santa Marija sand dune. During the last survey in April 2025, this species had become dominant in this pond.
19. *Salsola melitensis***.** An endemic of rupestral habitats, frequently occurring in Malta and Gozo as well as in Comino’s south and eastern cliffs, Cominotto and the islets. Interestingly, its first record dates back only a few decades, under the widely used taxon *Darniella melitensis* (Botsch) Brullo (Sciberras and Sciberras 2010). Earlier, it had been reported from Malta and Gozo as *Salsola vermiculata* L. (Sommier and Caruana Gatto 1915), but not from Comino. Examination of the historical literature suggests that, prior to its formal description as an endemic, the species was often confused with *Suaeda vera* Forssk., which was cited as *S.* *fruticosa* (L.) Delile (e.g., Sommier and Caruana Gatto 1915) from numerous rupestral localities, including Comino, so it is quite possible that records of *Suaeda* from Comino refer to *Salsola melitensis.*
20. *Schoenus nigricans.* One of the most remarkable findings in our surveys was the presence of a single tuft of *S. nigricans*, a legally protected, red-listed, threatened, and very rare species in Malta. It was found growing on Cominotto, possibly introduced from the established population at Marfa on the opposite shore of mainland Malta, which likewise grows on exposed rocky land near the coast.
21. *Sedum* spp. The four species recorded occur in low numbers on the Comino archipelago, unlike on the larger islands of Malta and Gozo, where *S. caeruleum* and *Petrosedum* (=*Sedum*) *sediforme* are widespread. On a positive note, Comino, Cominotto and il-Ħaġra l-Kbira harbours established populations of the rare and red-listed *S. littoreum*, as expected, recorded from shallow coastal areas.
22. *Senecio leucanthemifolius.* This red-listed species was found in small populations on the rupestral areas of the eastern cliffs of Comino Island, exhibiting the same habitat and morphology as populations in Malta and Gozo. Like other Asteraceae species, it is assumed to be grazed by rabbits, and on Comino, only some refuge populations remain.
23. *Senecio pygmaeus.* Difficult to spot due to its small size, but recorded in seven locations, two of which are on Cominotto. It prefers to grow on partly shaded, somewhat humid, rocky banks or rock basins near the shallow coast. It is a rare, subendemic, red-listed species.
24. *Sonchus* spp. An intriguing aspect of the flora of the Comino archipelago is the marked rarity of *Sonchus* spp., a genus otherwise widespread across the other islands of the Maltese archipelago. Presences are often interpreted as surviving individuals persisting in remote pockets or sheltered within stands of thyme (*Thymbra capitata*) or kidney vetch (*Anthyllis hermanniae*). However, a sizeable population was found close to the chapel and police station near Santa Marija, and consists of a different race characterised by dark green leaves, large plants, and abundant yellow glandular hairs on the stems at the inflorescences. The most plausible explanation for the species’ decline is heavy grazing pressure from the rabbit population, which was abundant on Comino for several decades and even centuries. Although reduced, rabbits can still occasionally be observed on the island today, suggesting that their impact on the vegetation has been long‑standing. More interesting, however, are the records of *Sonchus asper*, which is very rare on the Maltese islands and where it is erroneously recorded by everyone as the white-flowering *Sonchus oleraceus*.
25. *Sporobolus pungens.* The single, relatively large population was observed only on Cominotto but not on Comino Island or the other islets.
26. *Tetraclinis articulata.* A few individuals were planted in fields near the BirdLife premises in the mid-2000s and have now developed into large, mature trees. No autochthonous records or populations are known from Comino archipelago.
27. *Typha domingensis.* An interesting new record composed of a small and localised population growing around a small water reservoir. This record on Comino further supports the effective seed dispersal of this species, with seeds pioneering on Comino via wind dispersal from Gozo or mainland Malta. In the second half of the 20th century, it was considered threatened and had a restricted distribution, to the point that it was listed in the Red Data Book (Lanfranco 1989).
28. *Verbascum creticum.* This species was recorded and photographed by Jeffrey Sciberras in 2005, but has not been encountered since and is presumed extinct on Comino. It was located in the vicinity of the old hospital, and several attempts to locate it during these surveys were unsuccessful.
29. *Vitex agnus-castus.* A small copse of trees is extant close to Santa Marija sand dune. A photo of this bay, taken in the 1920s/1930s, shows that this species was widespread from the mouth of the Wied l-Aħmar to the sandy beach. Nowadays, most of this native population has been removed to make way for a tourist/leisure development (namely, a caravan park some 40 years ago). In contrast, some other *Vitex* trees were replaced by introduced *Tamarix* trees stretching to the shore, which slowly naturalised and extended to most of the beach area.
30. *Zannichellia melitensis/palustris.* Two populations in small rock pools have been observed, one on the northern side near the coast (Stephen Mifsud, pers. obs. 2007) and another discovered during our surveys close to an area known as Bajdet il-Fenek. This toponym is a direct Maltese translation of “orchids”, but it may possibly be a corruption of *Bejtet il-fenek*, which means “the rabbit’s nest”.
